# Theoretical insight of reactive oxygen species scavenging mechanism in lignin waste depolymerization products†

**DOI:** 10.1039/d3ra08346b

**Published:** 2024-02-20

**Authors:** Rahmanto Aryabraga Rusdipoetra, Hery Suwito, Ni Nyoman Tri Puspaningsih, Kautsar Ul Haq

**Affiliations:** a Bioinformatic Research Group, Research Centre of Bio-Molecule Engineering (BIOME), Airlangga University Jl. Ir. H. Soekarno Mulyorejo Surabaya Indonesia; b Department of Chemistry, Faculty of Science and Technology, Airlangga University Jl. Ir. H. Soekarno Mulyorejo Surabaya Indonesia kautsar.ul.haq@fst.unair.ac.id; c Proteomic Research Group, Research Centre of Bio-Molecule Engineering (BIOME), Airlangga University Jl. Ir. H. Soekarno Mulyorejo Surabaya Indonesia

## Abstract

Apart from natural products and synthesis, phenolic compounds can be produced from the depolymerization of lignin, a major waste in biofuel and paper production. This process yields a plethora of aryl propanoid phenolic derivatives with broad biological activities, especially antioxidant properties. Due to its versatility, our study focuses on investigating the antioxidant mechanisms of several phenolic compounds obtained from renewable and abundant resources, namely, syringol (Hs), 4-allylsyringol (HAs), 4-propenylsyringol (HPns), and 4-propylsyringol (HPs). Employing the density functional theory (DFT) approach in conjunction with the QM-ORSA protocol, we aim to explore the reactivity of these compounds in neutralizing hydroperoxyl radicals in physiological and non-polar media. Kinetic and thermodynamic parameter calculations on the antioxidant activity of these compounds were also included in this study. Additionally, our research utilizes the activation strain model (ASM) for the first time to explain the reactivity of the HT and RAF mechanisms in the peroxyl radical scavenging process. It is predicted that HPs has the best rate constant in both media (1.13 × 10^8^ M^−1^ s^−1^ and 1.75 × 10^8^ M^−1^ s^−1^, respectively). Through ASM analysis, it is observed that the increase in the interaction energy due to the formation of intermolecular hydrogen bonds during the reaction is an important feature for accelerating the hydrogen transfer process. Furthermore, by examining the physicochemical and toxicity parameters, only Hs is not suitable for further investigation as a therapeutic agent because of potential toxicity and mutagenicity. However, overall, all compounds are considered potent HOO˙ scavengers in lipid-rich environments compared to previously studied antioxidants.

## Abstract 2

Syringol derivatives outperform phenolic antioxidants in non-polar solvents. HT mechanism is the favoured mechanism to scavenge hydroperoxyl radical in substituted syringols. Based on ASM analysis, the hydrogen bond is the key factor in increasing both strain and interaction energy.

## Graphical abstract

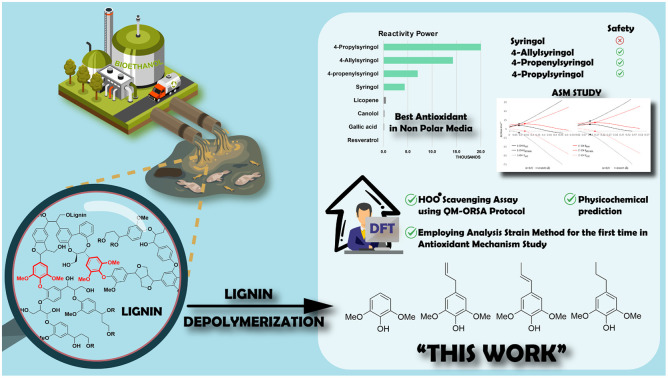

## Introduction

Lignin, the second most abundant biopolymer, is a by-product of biofuel and paper production, with only minimal reuse as a fuel source.^1–5^ The high production volume and the limited reusability options lead to lignin ending up as a waste, causing adverse effects on the aquatic ecosystem.^6–8^ Consequently, there has been a focus on developing various lignin-based materials, including bioplastics, supercapacitors, and batteries, to harness the technical lignin derived from pre-treatment steps.^9,10^ Lignin depolymerization is a crucial step for maximizing the economic value of the valorization process of lignin into value-added products.^11^ The depolymerization process involves the cleavage of β-aryl ether linkages, followed by subsequent reactions that give rise to several aromatic compounds, which are phenol, guaiacol, and syringol derivatives, that exhibit broad-spectrum application in human health and industry.^12,13^ Some are known to be used in liquor and perfumery due to unique and pleasant aroma characteristics.^14–17^ In addition, many of them have been proven to possess many biological activities such as antifungal, antibacterial, and antioxidant.^18,19^ The versatile antioxidant nature of these compounds enables their potential application in the pharmaceutical industry and other sectors. It could be employed as a preservative in dried cereal to prevent polyunsaturated fatty acid oxidation.^20,21^ Additionally, this concept proves advantageous in enhancing the lifespan and quality of non-edible industrial products, such as in plastic, engine, and biodiesel manufacturing.^22–25^

From three major structures of lignin units, syringol is reported to have potent antioxidant activity comparable with vitamin C and E.^26^ Furthermore, its derivatives exhibit better radical scavenging capabilities compared to other groups, as demonstrated through *in vitro* or *in silico* analyses.^27,28^ In addition, certain derivatives, such as canolol, syringic acid, and sinapinic acid, have been investigated further using density functional theory calculations.^29–32^ Among other derivatives, 4-allysyringol and 4-propenylsyringol are two of the many derivatives with under-explored antioxidant activities. Furthermore, these are included as staple derivatives of syringol produced from lignin depolymerization. They could be obtained using many methods, such as pyrolysis, oxidative, and reductive depolymerization, with various yields.^33–36^ Besides that, the presence of an unsaturated side chain is the main reason these compounds are selected for this work. A previous study claimed that it could increase the antioxidant potential of phenolic derivatives more than the parent structure.^37^ Moreover, the conjugative effect on this group results in good peroxyl radical scavenging activity in the lipid environment.^29,38^ This could lead to the development of neuroprotective drugs in the future.^39^

**Fig. 1:**
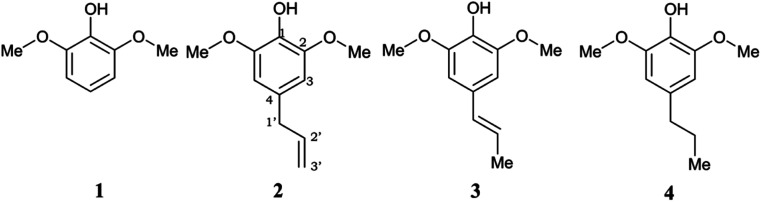
Chemical structures of the studied syringol derivatives and their atom numbering. (1) Syringol/Hs, (2) 4–allylsyringol/HAs, (3) 4-propenylsyringol/HPns, (4) 4-propylsyringol/HPs.

However, a deep investigation of the antioxidant mechanism of these two compounds is still not conducted. Thus, this work mainly consists of elucidating the radical scavenging of these compounds using computational studies. Along with 4-allylsyringol (HAs) and 4-propenylsyringol (HPns), syringol (Hs) and 4-propylsyringol (HPs) are also investigated to serve as the comparison structures (Fig. 1). We choose the hydroperoxyl radical (HOO˙) in this study as a target of scavenging for several considerations. This radical is the simplest form of the peroxyl radical (ROO˙) group, which is involved in oxidative damage, such as lipid peroxidation and brain tumor build-up.^40,41^ In addition, HOO˙ is basically the protonated form of the superoxide radical and is proposed to be more reactive than its conjugated base in living systems despite its low availability.^42^ However, the main reason is its mild reactivity, which can be seen from its half-life value.^43^ Thus, it can give a clear difference in the antioxidant efficiency of the tested compounds. Meanwhile, highly reactive radicals, such as hydroxyl radicals, tend to give diffusion-limited rates for any compounds, which biases the result. Thus, by modeling the scavenging mechanism of syringol derivatives with HOO˙, we will provide reliable kinetics data to gain insight into the structure–activity relationship for designing potent antioxidant compounds in specific environments.

In scavenging free radicals, antioxidant compounds provide various reaction mechanisms with different reactivity. From our previous research, one observed phenomenon is the predominance of the HT (hydrogen transfer) mechanism by phenolic groups in antioxidant compound.^44^ Meanwhile, other fragments, such as benzyl groups, exhibit higher activation energy. Thermodynamic and kinetic assessments are still insufficient to rationally explain the different required energy during the reaction.^45^ Thus, the activation strain model (ASM) was employed to study the properties and behavior of reactants throughout the reaction process.^46^ The aim is to provide a comprehensive explanation for uncovering the mechanism of free radical scavenging reactions by antioxidant compounds. Besides studying the antioxidant capacity of syringol compound derivatives, drug-likeness predictions were conducted to evaluate their suitability as bioactive compounds upon consumption.^47^ This kind of method is also essential preliminary research in drug development, aiming to lower the costs and minimize the risk of failure at later stages.^48–50^

## Theoretical methods

Gaussian16 software package was used for all density functional theory (DFT) calculations.^51^ The Minnesota function theory level, M06-2X, was utilized for every calculation performed during this study. This particular approach was chosen due to its ability to offer more precise and reliable geometric and energetic data compared to alternative DFT functions, notably the B3LYP function.^52,53^ The Pople basis set with diffusion and polarization functions, 6-311++G(d,p), was employed in this study due to its common application among researchers studying free radical scavenging activity.^54^ The density-based implicit solvation model (SMD) was performed in the calculations to mimic the physiological media for the free radical scavenging process.^55^ Water and pentyl ethanoate were used as representative media for body fluids and lipid bilayer membranes of cells, respectively.^56,57^

In open-shell systems, unrestricted calculations have been conducted. Local minima and transition states (TS) are distinguished by the presence of imaginary frequency (^i^*f* = 0 and ^i^*f* = 1, respectively). Moreover, TS structures are also validated with intrinsic reaction coordinate (IRC) calculation to assure that pre- and post-complex are connected. Lastly, thermodynamic correction at 298.15 K was applied for all relative energies. Also, the standard state conversion into solution (eqn (1)) was included for the bimolecular reaction.^58^ Moreover, Okuno's correction (eqn (2)) is utilized likewise to consider entropy loss caused by the solvent cage effect.^30,59^ Those equations are described as follows.1
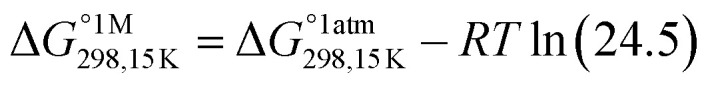
2
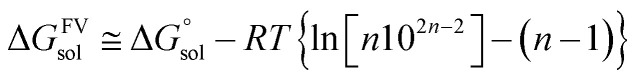


The molar fraction of neutral and ionic species for each compound was determined by modifying the simple Henderson–Hasselbach equation into the following equations (eqn (3) and (4)) described below. For any compound whose experimental p*K*_a_ values are still unknown, the parameter fitting (PF) method was conducted. This method is proven to give similar results with the experimental data for phenolic compounds at any theoretical level.^60^3
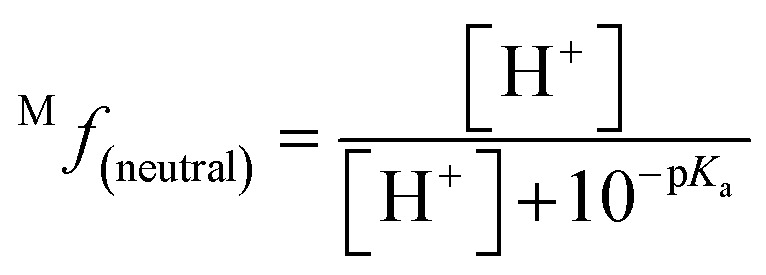
4
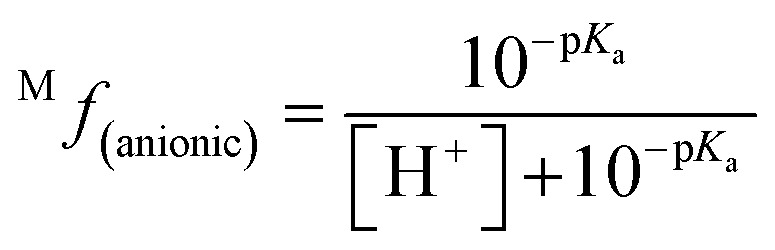


In the thermodynamic and kinetic study, the QM-ORSA protocol was performed.^61^ In both the media, two main radical scavenging mechanisms were studied, namely, HT (Hydrogen Transfer) and RAF (Radical Adduct Formation). Only oxygen atoms in phenolic rings, benzylic, and allylic C sp^3^ atoms, which could serve as hydrogen donors, were considered in the HT mechanism. Meanwhile, all unsaturated carbons were considered in modeling the RAF mechanism. Besides, Single Electron Transfer (SET) and Sequential Proton Loss Electron Transfer (SPLET) mechanisms were also included in polar media because of the presence of anionic species. On the other hand, these species could not be formed spontaneously in the lipid media and have been proven by prior work.^37^ Thus, only neutral species of syringol derivatives were studied in the lipid environment.

Only sites undergoing exergonic reaction (Δ*G* < 0) in each compound were followed by kinetic calculation. Eyring equation (eqn (5)) was used to calculate the rate constant (*k*^TST^), which also included Eckart tunneling correction and the number of symmetrical reaction paths (*σ*).^62–67^5
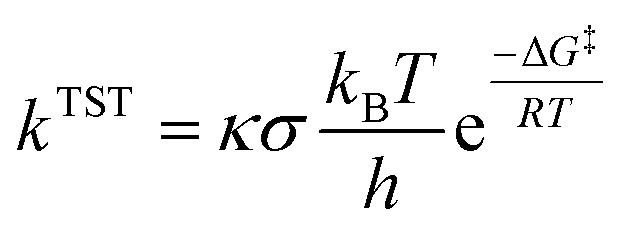
where *T* is the temperature in Kelvin, *R* is the gas constant in kcal K^−1^ mol^−1^, *h* is Planck's constant, *k*_B_ is Boltzmann's distribution constant, and Δ*G*^‡^ is the activation energy. The Eyringpy program was utilized to acquire the tunneling transmission coefficient (*κ*), as done by prior works.^44,68–71^ For any scavenging mechanism that involved electron transfer, such as SET and SPLET, Marcus theory (eqn (6)) was performed to find their activation energy 
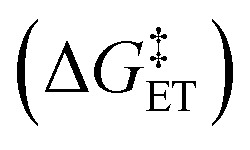
.^72,73^6
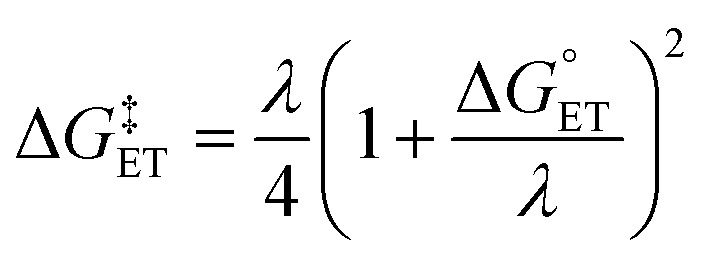
7
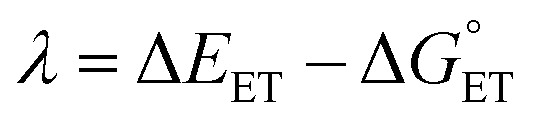


The value of reorganization energy (*λ*) could be approximated using 
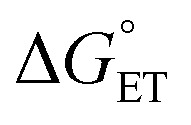
 and Δ*E*_ET_ (eqn (7)) values, where 
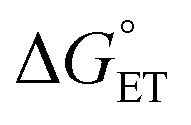
 is the Gibbs energy of reaction, whereas Δ*E*_ET_ describes the energy difference between the reactant and vertical product. Diffusion control was considered for any rate constant that exceeds 10^8^ M^−1^ s^−1^.^54^ For that, Collins–Kimball theory (eqn (8)), Smoluchowski's rule (eqn (9)), and the Stokes–Einstein equation (eqn (10)) were employed to obtain a realistic rate constant (*k*_app_).^74–77^ All equations that have been mentioned before are described as follows.8
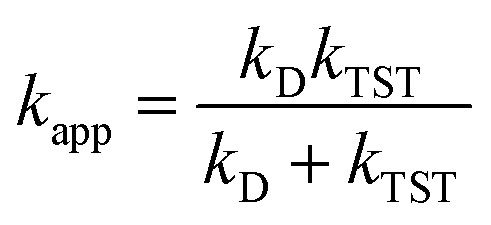

9 *k*_D_ = 4π*R*_AB_*D*_AB_*N*_A_

10
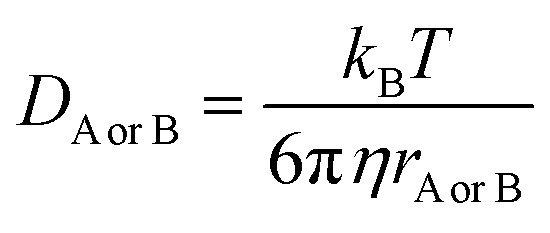
where *k*_D_ is the steady state Smoluchowski rate constant, *R*_AB_ is the reaction distance, *D*_AB_ is the total mutual diffusion coefficient of free radical (A) and syringol derivatives (B), *N*_A_ is Avogadro's number, *η* is the viscosity of the solvent, and *r* is the radius of the solute. For HT and RAF mechanisms, the reaction distance was measured from two heavy atoms in transition states, which were involved in the transfer process. Meanwhile, the reaction distance in SET and SPLET mechanisms was simply the total radius of solutes.

The IRCs obtained were then used in activation strain analysis. Each potential energy (Δ*E*(ζ)) at the reaction coordinate point *ζ* was decomposed into strain destabilization energy (Δ*E*_strain_(*ζ*)) and interaction energy (Δ*E*_int_(*ζ*)), as detailed in eqn (11). The interaction energy was obtained from the difference between the complex structure energy and strain energy relative to the reactant complex.^78^

11 Δ*E*(*ζ*) = Δ*E*_strain_(*ζ*) + Δ*E*_int_(*ζ*)

Strain energy is the energy required to deform the reactant structure to adopt the structure present at each reaction coordinate point. Therefore, this energy value is always positive. According to eqn (12), strain energy was obtained by summing the relative single-point energies of the syringol derivative structure (Δ*E*_strain syd_(*ζ*)) and the radical (Δ*E*_strain rad_(*ζ*)) at each point on the IRC diagram.^79^ When performing single-point calculations, the syringol derivative structure was considered to have singlet spin multiplicity, while the HOO˙ radical has doublet spin multiplicity.

12 Δ*E*_strain_(*ζ*) = Δ*E*_strain syd_(*ζ*) + Δ*E*_strain rad_(*ζ*)

The three types of obtained energy are represented in a 2D diagram, with the distance of the key bond in the reaction as the abscissa. In the HAT mechanism, these are the broken bonds of O⋯H or C⋯H, while the formation of the C⋯O bond was observed in the RAF mechanism.

## Results and discussion

### Acid–base equilibria

Based on the prediction results, the parameter fitting method demonstrated reasonably good accuracy, although it is slightly lower than the actual values.^80^ The predictions also indicate that syringol and its derivatives are classified as weak acids. This can be related to the formation of intramolecular hydrogen bond between 1-OH with *O*-methoxy, which increases the deprotonation energy.^81,82^ At the physiological pH, syringol derivatives are highly available in a neutral form. As shown in Table 1, all of them exist only in the range from 0.23 to 0.48% as anions. Nonetheless, this amount is still considered in the subsequent analysis because it tends to react within the diffusion rate limits.^83,84^

**Table 1:** p*K*_a_ values and molar fraction of syringol derivatives at physiological pH. HA: neutral species, A^−^: anionic species^a^

Compound	p*K*_a_ calc.	p*K*_a_ exp.^a^	^M^ *f* HA (%)	^M^ *f* A^−^ (%)
Hs	9.86	9.98	99.73	0.27
HAs	9.89	10.05	99.77	0.23
HPns	9.71	—	99.52	0.48
HPs	10.03	—	99.77	0.23

a Obtained from ref. 80.

### Thermodynamic study

From the data in Table 2, all sites in the HAT mechanism are exergonic. The presence of substituents at the para (C-4) position appears to enhance the spontaneity of the reaction. Similar results have been found in syringol derivatives reported in other studies, such as canolol,^29^ sinapinic acid,^31^ and syringic acid.^32^

**Table 2:** Gibbs free energy of the reaction (Δ*G*) in kcal mol^−1^ at 298.15 K for the modeled reaction pathways. HA: neutral species, A^−^: anionic species, PE: pentyl ethanoate solution, W: aqueous solution

Mechanism, site	HA (PE)	HA (W)	A^−^ (W)	HA (PE)	HA (W)	A^−^ (W)
	Hs			HPs		
HT, 1-OH	−6.91	−10.29		−8.47	−12.61	−6.49
HT, 1′-CH				−1.03	−3.38	
SET		24.45			20.44	
SPLET			−3.63			−9.05
RAF, C-1	4.64	7.42	6.87	7.66	5.29	5.00
RAF, C-2	14.33	13.1	3.14	13.36	12.18	1.58
RAF, C-3	16.18	14.91	14.11	13.7	13.4	10.67
RAF, C-4	12.66	11.68	4.36	12.23	11.07	2.97

	HAs			HPns		
HT, 1-OH	−8.23	−12.38		−9.17	−13.41	
HT, 1′-CH	−10.63	−12.90	−16.50			
HT, 3′-CH				−4.94	−7.65	−10.63
SET		21.80			21.17	
SPLET			−8.37			−8.87
RAF, C-1	7.31	5.88	5.81	5.00	2.82	2.08
RAF, C-2	13.5	12.17	1.4	15.34	13.81	2.75
RAF, C-3	12.89	12.1	10.95	11.35	9.69	7.57
RAF, C-4	10.43	9.09	1.04	15.4	13.33	6.32
RAF, C-1′				6.03	3.6	2.4
RAF, C-2′	1.63	−0.15	−0.58	−5.67	−6.8	−10.72
RAF, C-3′	0.75	−0.18	0.01			

Hydrogen abstraction from the phenolic sites reveals that the stability of the formed radical compounds can be ranked as follows: HPns > HPs ≈ HAs > Hs. The position of the double bond in the propanoid group plays a crucial role to enhance product stability as the size of electron delocalization contributes to lowering the free energy of the reaction.

For carbon-centered radicals, the stability order is HAs > HPs > HPns. Unlike phenolic radical stability, an increased number of groups involved in resonance effects is an important feature to make the reaction more spontaneous. This is particularly noticeable in the HAs, which has both allylic and benzylic groups, allowing for greater electron delocalization. In contrast, the carbon radical in HPns is delocalized by only one group.

In polar solvents, antioxidant compounds may scavenge free radical activity through electron transfer mechanisms, specifically SPLET and SET. However, we found that SPLET mechanism showed thermodynamic viability, whereas the SET reaction did not. These results differ slightly from other lignin derivative groups, such as guaiacol, where both mechanisms are less favored.^20^ Among the four studied compounds, electron transfer reaction in HPs is the most spontaneous. It appeared that the electron-donating power at the C-4 position influences this reaction.

In contrast to the RAF mechanism, it is difficult for all syringol derivatives to form products in all studied solvents. The loss of aromaticity of the adduct formed leads to unfavorable scavenging activity by the phenolic ring. This effect was not observed in the propanoid group, where several reaction sites showed various ranges of viability. To gain an in-depth explanation of the ring's impact on the spontaneity of RAF mechanism, activation strain analysis was conducted and discussed in later section.

The position of the double bond in the propanoid group demonstrated a significant influence on the spontaneity of the RAF mechanism. For instance, in HAs, the isolated sp^2^ carbon atom separated from the resonance system leads to a decrease in the stability of the radical products. This results in a negligible impact of solvent polarity and charge on enhancing the delocalization effects, making an insignificant trend of spontaneity.

### Kinetic study

Based on thermodynamic studies, it can be observed that the hydroperoxyl radical scavenging reaction of the syringol derivative occurred more spontaneously through the HT mechanism in all the solvents. However, kinetic analysis must still be carried out to quantify the contribution of each mechanism based on kinetic parameters, namely, activation energy and reaction rate constant. The transition structures at the HT and RAF sites, which are exergonic, are sought, and their activation energies are determined. The processed kinetic data has been summarized and can be seen in Table 3.

**Table 3:** Gibbs free energy of activation (Δ*G*^‡^) in kcal mol^−1^ at 298.15 K, quantum tunneling coefficient (*κ*), and realistic rate constant (*k*_app_) for viable reaction pathways. HA: neutral species, A^−^: anionic species, PE: pentyl ethanoate solution, W: aqueous solution^a^

Mechanism, site	HA (PE)			HA (W)			A^−^ (W)		
	Δ*G*^‡^ (kcal mol^−1^)	*κ*	*k*_app_ (M^−1^ s^−1^)	Δ*G*^‡^ (kcal mol^−1^)	*κ*	*k*_app_ (M^−1^ s^−1^)	Δ*G*^‡^ (kcal mol^−1^)	*κ*	*k*_app_ (M^−1^ s^−1^)
Hs									
HT, 1-OH	12.82	565.4	1.48 × 10^7^	12.72	511.8	1.60 × 10^7^			
LET							2.15	19.0^a^	7.90 × 10^9^

HAs									
HT, 1-OH	11.89	383.7	4.85 × 10^7^	11.23	305.0	1.12 × 10^8^			
HT, 1′-CH	16.66	134.8	1.09 × 10^4^	15.48	56.7	3.37 × 10^4^	3.68	4.2	2.95 × 10^9^
SPLET							1.54	19.3^*a*^	8.09 × 10^9^
RAF, C-2′				17.83	1.6	18.0	18.37	1.6	7.18
RAF, C-3′				16.10	1.6	3.30 × 10^2^			

HPns									
HT, 1-OH	12.38	437.1	2.40 × 10^7^	10.96	74.1	4.44 × 10^7^			
HT, 3′-CH	18.61	186	8.38 × 10^2^	16.36	76	1.52 × 10^4^	5.76	11.9	2.86 × 10^9^
SPLET							1.20	18.2^a^	8.16 × 10^9^
RAF, C-2′	15.74	1.4	5.33 × 10^2^	13.40	1.4	2.77 × 10^4^	3.54	1.1	2.40 × 10^9^

HPs									
HT, 1-OH	11.45	294.3	1.13 × 10^8^	11.03	442.7	1.50 × 10^8^			
HT, 1′-CH	17.30	41.1	1.27 × 10^3^	16.47	93.1	2.27 × 10^3^	5.69	8.2	2.81 × 10^9^
SPLET							1.32	19.1^a^	8.14 × 10^9^

a Reorganization energy.

Consistent with the thermodynamic data, substituents also enhance the reactivity of syringol derivatives in all available reaction pathways. Apart from being examined through thermochemical analysis, this trend can also be characterized by the topological structure of transition states based on Hammond's postulate. For HT in the 1-OH mechanism, a higher order of the phenolic OH bond depicts an “earlier” transition state (TS), correlating with the lower activation energy.

**Fig. 2:**
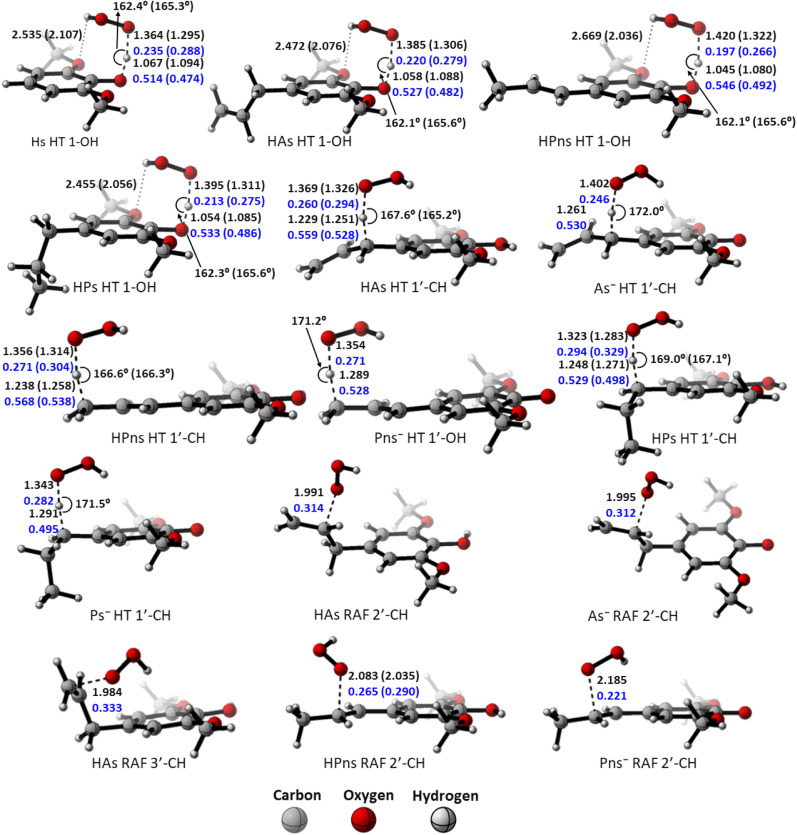
Transition state structures of the HT mechanism at the O-phenolic site in each syringol derivatives, along with bond lengths (in black), bond angles (in black), and Wiberg bond orders (in blue) in water and pentyl ethanoate (in bracket).

However, compared to the propanoid group, this relationship could not be applied. As seen in Fig. 2, all carbon-centered reaction sites in all the derivatives have higher activation energy despite possessing an “earlier” TS structure. This is related to the formation of the hydrogen bond between the *O*-methoxy and the HOO˙ radical in the 1-OH transition states, which assists in stabilizing the structure, thus reducing the energy barrier.^85^ Therefore, the hydrogen transfer by 1-OH proceeds faster, despite its “later” TS structure.

The “earlier” TS characteristics also show a fairly good correlation with increased reactivity in the RAF mechanism. HPns, which has a smaller C–O bond order compared to HAs, possess an ‘earlier’ TS structure, resulting in a higher reaction rate.

However, despite having a higher activation energy, HPns actually exhibits a better radical scavenging rate through the RAF site compared to canolol in all solvents.^29^ It seems that the difference in the quantum tunneling effect on these two compounds underlies this phenomenon.

The polar environment also influences the energy required for each site to react *via* the HT and RAF mechanisms. In an aqueous environment, all energy barriers for each reaction site are slightly lower than in a pentyl ethanoate environment. Decreasing energy barrier can also be observed from the ‘earlier’ TS at all sites in an aqueous solvent.

Among the two available forms, the anion species is the most reactive form in scavenging hydroperoxyl radicals in all the existing mechanism pathways. This species has lower energy barriers by 11 kcal mol^−1^ at all the sites and reacts within the diffusion limit range. However, it may not necessarily be dominant due to its availability, which affects its reactivity.

To obtain the overall rate constant (*k*_overall_) for each reaction site in all the pathways in a non-polar solvent, the values of *k*_app_ are summed following eqn (13). Meanwhile, in an aqueous solvent, these values are first multiplied by the molar fraction of each syringol derivative species at physiological pH to obtain the molar fraction-weighted rate constant (*k*_f_), which is then summed to obtain the overall rate constant in hydroperoxyl radical scavenging, as indicated in eqn (14)–(16).13
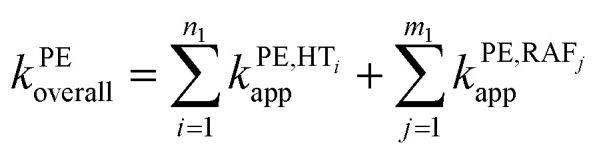
14
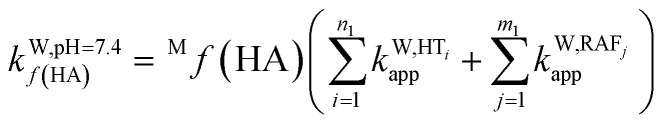
15
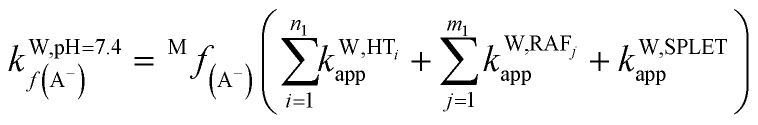
16
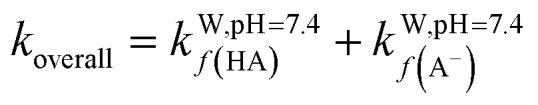


From the overall rate constant values presented in Table 4, the presence of aliphatic groups in syringol derivatives resulted in the highest reaction rate constants according to the following order: HPs > HAs > HPns > Hs. The enhanced antioxidant capacity of the substituted syringol derivatives over syringol compounds can be attributed to the increased electron-donating power at the para position. These results aligned with the study conducted by Michalík *et al.* (2019) on phenolic compound derivatives.^86^

**Table 4:** Realistic rate constant (*k*_app_), molar fraction-weighted rate constant (*k*_f_), pathways, Branching ratio (*Γ* (%)), and overall rate constant (*k*_overall_) of the studied syringol derivatives. HA: neutral species, A^−^: anionic species, PE: pentyl ethanoate solution, W: aqueous solution

Mechanism, site		HA (PE)		(W)				
				HA		A^−^		*Γ* (%)
		*k*_app_ (M^−1^ s^−1^)	*Γ* (%)	*k*_app_ (M^−1^ s^−1^)	*k*_f_ (M^−1^ s^−1^)	*k*_app_ (M^−1^ s^−1^)	*k*_f_(M^−1^ s^−1^)	
Hs	HT, 1-OH	1.48 × 10^7^	100.0	1.60 × 10^7^	1.59 × 10^7^			43.4
	SPLET					7.90 × 10^9^	2.07 × 10^7^	56.6
	*k*_overall_	**1.48 × 10** ^**7**^		**3.66 × 10** ^**7**^				
HAs	HT, 1-OH	4.85 × 10^7^	100.0	1.12 × 10^8^	1.12 × 10^8^			81.9
	HT, 1′-CH	1.09 × 10^4^	∼0.0	3.37 × 10^4^	3.36 × 10^4^	2.95 × 10^9^	6.59 × 10^6^	4.8
	SPLET					8.09 × 10^9^	1.81 × 10^7^	13.3
	RAF, 2′-C			18.0	17.9	7.18	1.60 × 10^−2^	∼0.0
	RAF, 3′-C			3.30 × 10^2^	3.29 × 10^2^			∼0.0
	*k*_overall_	**4.86 × 10** ^**7**^		**1.36 × 10** ^**8**^				
HPns	HT, 1-OH	2.40 × 10^7^	100.0	4.44 × 10^7^	4.41 × 10^7^			40.7
	HT, 3′-CH	8.38 × 10^2^	∼0.0	1.52 × 10^4^	1.51 × 10^4^	2.86 × 10^9^	1.37 × 10^7^	12.6
	SPLET					8.16 × 10^9^	3.91 × 10^7^	36.1
	RAF, 2′-C	5.33 × 10^2^	∼0.0	2.77 × 10^4^	2.65 × 10^4^	2.40 × 10^9^	1.15 × 10^7^	10.6
	*k*_overall_	**2.40 × 10** ^**7**^		**1.09 × 10** ^**8**^				
HPs	HT, 1-OH	1.13 × 10^8^	100.0	1.50 × 10^8^	1.50 × 10^8^			85.4
	HT, 1′-CH	1.27 × 10^3^	∼0.0	2.27 × 10^3^	2.26 × 10^3^	2.81 × 10^9^	6.55 × 10^6^	3.7
	SPLET					8.14 × 10^9^	1.90 × 10^7^	10.9
	*k*_overall_	**1.13 × 10** ^**8**^		**1.75 × 10** ^**8**^				

**Fig. 3:**
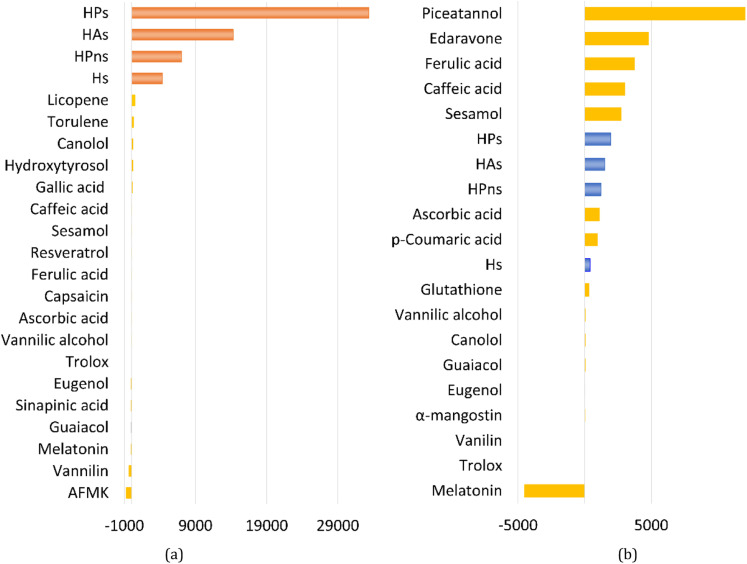
Reactivity power of the studied syringol and other known antioxidants in (a) non-polar media and (b) polar media relative to Trolox.

Overall, all four syringol derivatives react approximately five times faster in scavenging peroxyl radicals compared to lipid peroxidation reactions.^42^ As a result, all of them can be considered as active antioxidant compounds. Under hydrophobic conditions, we found that the reaction rate constants of these compounds surpass those of the current best natural antioxidants in the scavenging HOO˙ radical, namely, lycopene (BPW91/6-31G(d,p), *k*_overall_ = 1.69 × 10^6^ M^−1^ s^−1^) and torulene (BPW91/6-31G(d,p), *k*_overall_ = 9.47 × 10^5^ M^−1^ s^−1^).^38^ Moreover, some potent antioxidants, namely, resveratrol (M05-2X/6-31++G(d,p), *k*_overall_ = 1.31 × 10^4^ M^−1^ s^−1^),^87^ ascorbic acid (M05-2X/6-31+G(d,p), *k*_overall_ = 5.71 × 10^3^ M^−1^ s^−1^),^61^ and trolox (M05-2X/6-31+G(d,p), *k*_overall_ = 3.40 × 10^3^ M^−1^ s^−1^),^88^ lie far below these four compounds with a significant difference of up to 30 000 times. On aqueous solvent, Hs as the parent structure has comparable reactivity with glutathione (M05-2X/6-311+G(d,p), *k*_overall_ = 2.69 × 10^7^ M^−1^ s^−1^) and ascorbic acid (M05-2X/6-31+G(d,p), *k*_overall_ = 9.97 × 10^7^ M^−1^ s^−1^) in line with *in vitro* observation.^26,89^ However, all derivatives react more slowly compared to sesamol (M05-2X/6-311+G(d,p), *k*_overall_ = 2.44 × 10^8^ M^−1^ s^−1^),^90^ caffeic acid (M05-2X/6-311+G(d,p), *k*_overall_ = 2.69 × 10^8^ M^−1^ s^−1^),^91^ and piceatannol (M05-2X/6-31++G(d,p), *k*_overall_ = 1.13 × 10^9^ M^−1^ s^−1^).^87^ Nevertheless, based on Fig. 3, all four studied syringol derivatives can be categorized as promising novel antioxidants in scavenging hydroperoxyl radicals in an aqueous environment.

In the case of Hs, its scavenging ability in a non-polar environment is solely influenced by the HT mechanism at the 1-OH site because there are no other reaction pathways available. For the other three compounds that possess multiple available reaction pathways, the branching ratio (*Γ*_i_) calculations were carried out to determine the influence of each pathway on the scavenging reactivity using the equation below.17
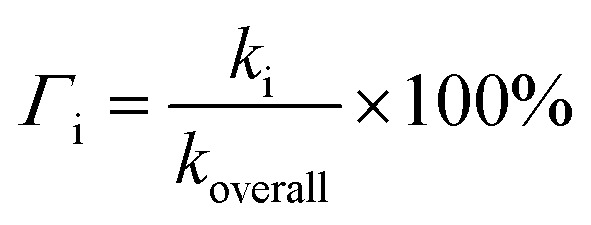


These values indicate that hydrogen transfer from the 1-OH site remains the sole pathway affecting the total rate constant, while other site contributions are negligible. Thus, the scavenging process of the HOO˙ radical through hydrogen abstraction from the phenolic site is a key factor in designing derivatives of phenolic-based antioxidants in a non-polar environment. This argument is supported by similar research on amino pyridinol derivatives,^92^ sinapinic acid,^31^ ferulic acid,^91^ eugenol, guaiacol, and vanillin,^37^ which show similar findings.

**Fig. 4:**
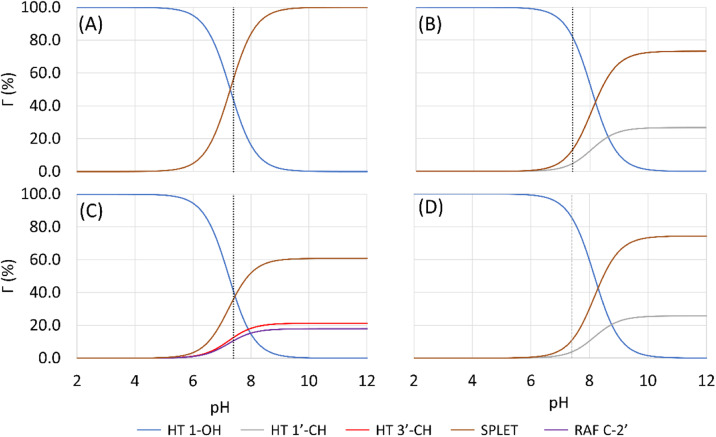
Influence of pH on the branching ratio of available reaction pathways in (A) Hs, (B) HAs, (C) HPns, and (D) HPs.

In an aqueous environment, the HT mechanism at the 1-OH site also remains as the dominant pathway for nearly all the studied syringol derivatives in scavenging hydroperoxyl radicals. This domination was applied only within the acidic to physiological pH range, as seen in Fig. 4. Under basic conditions, SPLET becomes the key pathway in providing hydroperoxyl radical scavenging reactivity for syringol derivatives due to the increased availability of anionic species within that pH range.

**Fig. 5:**
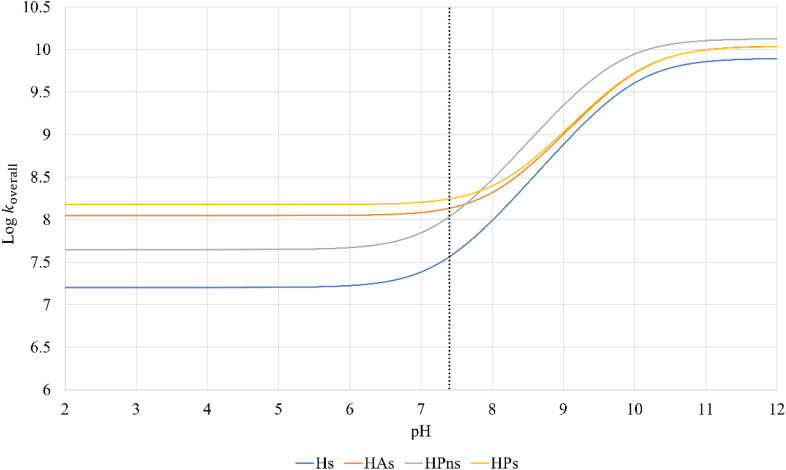
pH influences in the overall rate constant of syringol derivatives.

The reactivity of syringol derivatives was also observed within a specific pH range, which is 2–12. In an acidic environment (pH = 2–6), all derivatives exhibited good peroxyl radical scavenging capacity, as seen in Fig. 5. Within this range, the reactivity order of the studied syringol derivatives was the same as that at the physiological pH, with HPs being the most reactive one. However, in an alkaline environment, there was a significant reactivity increase and change in the order among the four syringol derivatives. HPns becomes the most reactive derivative one among the others at pH = 8-12. Meanwhile, HAs and HPs react with hydroperoxyl radicals at nearly the same rate. Only syringol consistently exhibited less reactive scavenging ability compared to the other three compounds in every observed pH range.

### Elucidating HT and PCET pathways

In antioxidant mechanisms, the hydrogen atom can be abstracted simultaneously in the form of protons together with electron, a process known as PCET (proton-coupled electron transfer). Therefore, the process yields the same products as HT, making it difficult to distinguish between them. Several methods have been proposed to differentiate these two mechanisms, although such a determination is not a straightforward matter.

The charge (*Q*) and spin density of transferred hydrogen atom (ASD) analysis at the transition state (TS) were performed according to the method reported by Galano and are summarized in Table 5. In the molecular form, the spin population on the migrating hydrogen atom at all the reaction sites indicated small negative values, consistent with the HT process.^93^ On the other hand, hydrogen transfer by the CH site in anionic species had a positive population value much greater than that reported previously. In connection with the charge analysis, these species can proceed through the HT mechanism due to their smaller charge value. However, this analysis method did not provide a satisfactory explanation as stated by Galano.

**Table 5:** Atomic spin densities (ASD) and charge (*Q*) of migrating H atom from natural orbital population analyses. HA: neutral species, A^−^: anionic species, PE: pentyl ethanoate solution, W: aqueous solution

Compounds, site		HA (PE)		(W)			
				HA		A^−^	
		ASD	Q	ASD	Q	ASD	Q
Hs	1-OH	−0.034	0.466	−0.033	0.478		
HAs	1-OH	−0.033	0.467	−0.032	0.480		
	1′-CH	−0.041	0.325	−0.032	0.321	0.021	0.385
HPns	1-OH	−0.024	0.492	−0.031	0.483		
	3′-CH	−0.038	0.316	−0.027	0.313	0.019	0.374
HPs	1-OH	−0.032	0.468	−0.032	0.480		
	1′-CH	−0.034	0.328	−0.024	0.324	0.026	0.386

For this reason, SOMO (Single Occupied Molecular Orbital) topology analysis was then performed as it provides a reasonable description of the PCET mechanism. The observation indicated that the orbital densities at all the sites form nodes aligned to the hydrogen abstraction pathway, implying the HT mechanism. However, the cisoid form of TS at 1-OH obtained from the experiment showed that this analysis is less significant for deducing it as the HT mechanism because the previous study reported that the HT mechanism occurs at those sites possessing planar or transoid TS structure, which affects our findings.^94,95^

Therefore, the observation was continued using HOMO (Highest Occupied Molecular Orbital) shape as recommended by DiLabio.^96^ As seen in Fig. S5,† all sites exhibited overlapping interactions between the π-orbital of the phenolic ring and the lone pair electron of the radical. This enables the transfer of electrons through different orbitals, leading to the PCET mechanism. The presence of this interaction also resulted in the orbital density of the SOMO not being the same as the topology of the conventional PCET mechanism.^97^

However, the presence of similar overlap orbitals occurring at the benzylic site made the determination of the PCET mechanism a little doubtful as previous research found that those sites undergo the HT mechanism.^93,98^ Also, if HOMO analysis is ignored, SOMO analysis on 1-OH site gives different results from the previous study.^69^ Hence, we still could not give a final hydrogen transfer mechanism for the radical scavenging activity of syringol derivatives.

### Decomposition energy analyses by activation strain model (ASM)

Based on the previous and current studies, the reactivity of the HT mechanism on benzylic C–H is consistently lower compared to the phenolic OH, even though the reaction is generally thermodynamically favoured due to the high activation energy. However, there is no in-depth study to explain this phenomenon. Therefore, we attempt to use the activation strain model to quantify the factors influencing this value.^78^

**Fig. 6:**
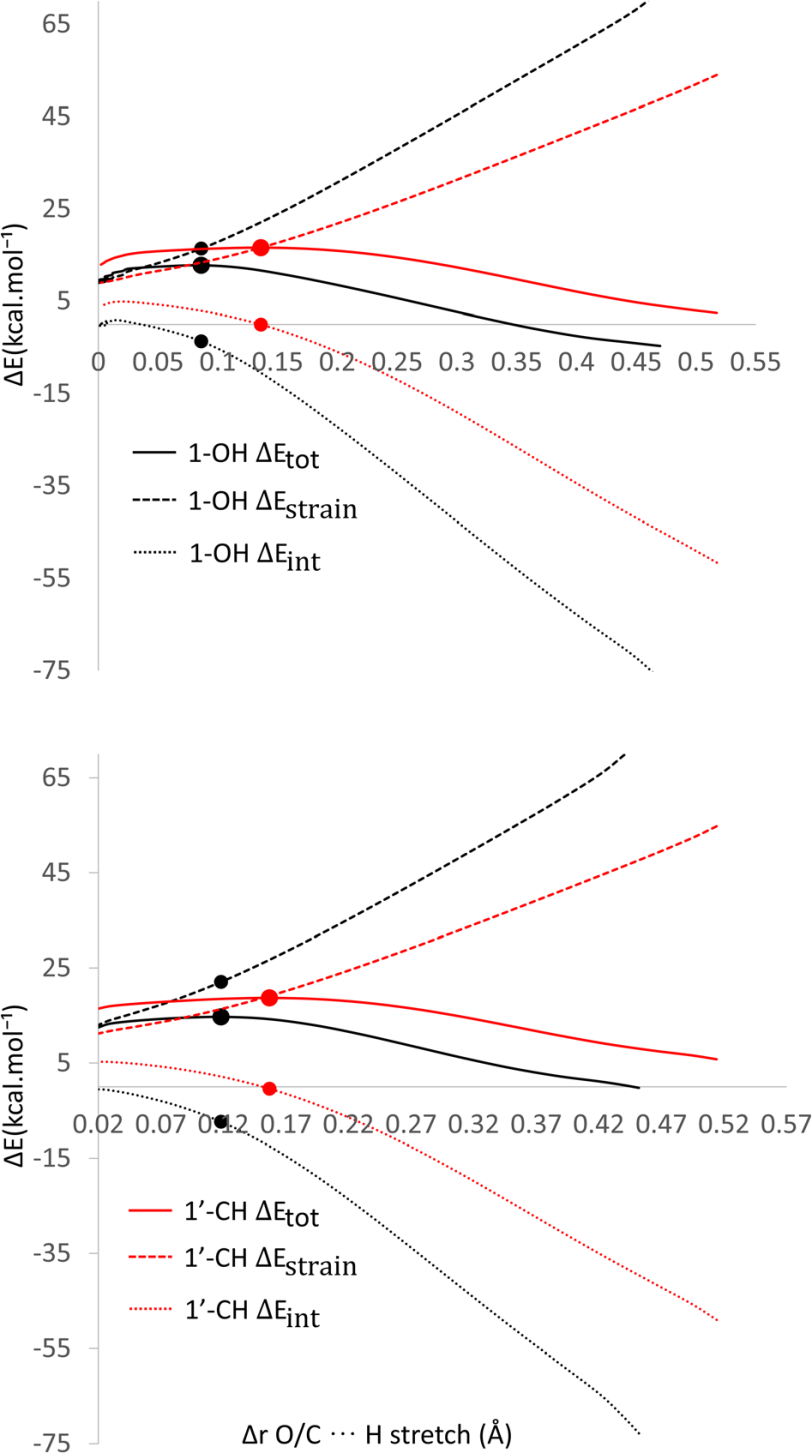
Activation strain model of the HT mechanism of 1-OH (black) and 1′-CH (red) in (a) water and (b) pentyl ethanoate using HAs as the model.

**Fig. 7:**
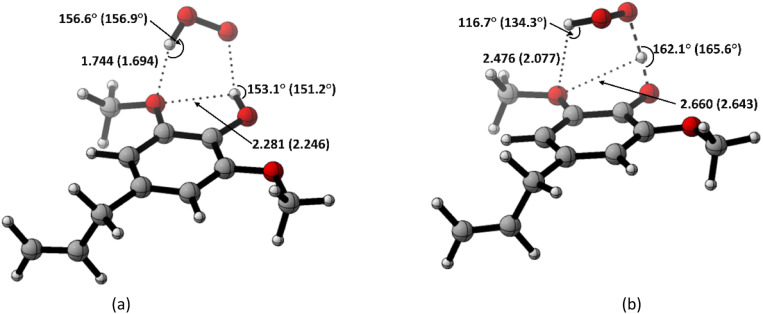
Hydrogen bond in (a) the reactant complex and (b) the transition structure in HT mechanism of HAs. Bond length in the studied media is shown as *water (pentyl ethanoate)*.

As observed in Fig. 6, the destabilization strain energy at 1-OH is significantly higher during the reaction compared to 1′-CH in all solvents. This is because of the intramolecular hydrogen bond that suppresses the movement of the phenolic H-atom to form the transition structure, as depicted in Fig. 7. This does not occur at the 1′-CH site, allowing the bond to transform freely with lower energy into the TS geometry.

However, during the reaction, the formation of a hydrogen bond between the HOO˙ radical and the syringol derivatives at the 1-OH site becomes a key factor in increasing the interaction energy, thus lowering the activation energy. The absence of this interaction at the 1′-CH site resulted in insufficient interaction energy to stabilize the strain energy, leading to a high activation energy.

**Fig. 8:**
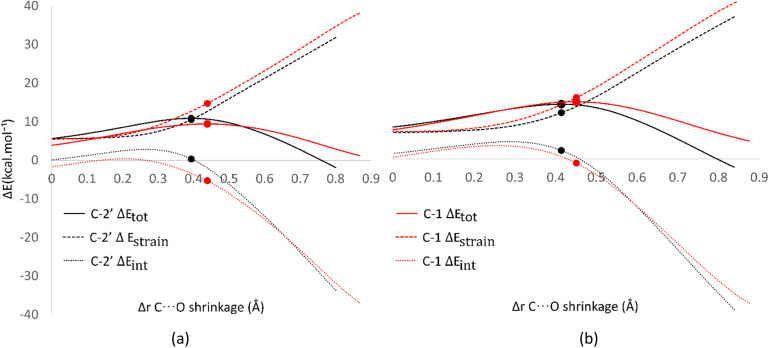
Activation strain model of the RAF mechanism between C-2′ (black) and C-1 (red) in (a) water and (b) pentyl ethanoate using HPns as the model.

Analysis was also conducted on the RAF mechanism to determine the factors causing the aromatic sites to react non-spontaneously with the HOO˙ radical. For this purpose, the potential energy during the reaction at the C-1 and C-2′ sites were decomposed and compared. As presented in Fig. 8, the deformation of the alkene structure requires lower energy compared to the aromatic ring. Besides the loss of π electron delocalization, the high deformation energy at the C-1 site is also due to the additional energy required to disrupt the relatively rigid planarity of the aromatic ring.

Surprisingly, the formation of radical adducts by aromatic sites has higher interaction energy. It appears that there are non-covalent interactions between the HOO˙ radical and the aromatic ring during the transition structure formation. The solvent polarity also seems to assist in enhancing these interactions, making the formation of transition structures in aqueous solvent more favorable.

However, the loss of ring aromaticity after the TS formation resulted in a decrease in the interaction energy, which is insufficient to stabilize the increasingly higher strain energy. It is also shown that the interaction energy plot also flattens, leading to an intersection with the interaction energy plot of C-2′. This resulted in the formation of an unstable product that can be readily dissociated back into reactants.

### Biocompatibility prediction

Although all the studied compounds have been proven to be effective antioxidants, the physicochemical and toxicity parameters need to be considered to determine whether these four compounds are suitable for consumption. Therefore, the SwissADME program was employed to calculate the physicochemical and pharmacokinetic properties that are assessed in the ADMET analysis.^99^ The parameters used refer to the Lipinski,^100^ Ghose,^101^ and Veber^102^ rules, as detailed in Table 6. Meanwhile, the LD_50_ values and mutagenic potential (*M*) are used to assess the toxicity of syringol derivatives, calculated using the T.E.S.T (Toxicity Estimation Software Tool) program.^103,104^

**Table 6:** Physicochemical analysis parameters and their values to satisfy Lipinski's, Ghose's, and Veber's criteria

Parameter	Symbol	Threshold	Unit
Partition coefficient octanol/water	log *P*	−0.4–5.0	
Polar surface area	PSA	≤140	Å^2^
Number of heavy atoms	^X^At	20–70	
Molecular weight	MW	160–480	g mol^−1^
Number of H-bond acceptors	HB^A^	≤10	
Number of H-bond donors	HB^B^	≤5	
Number of rotatable bonds	RB	≤10	
Molar refractivity	^M^ *R*	40–130	m^3^ mol^−1^

All the obtained values are then summarized in Table 7. From the ADME analysis, all syringol derivatives have met the necessary criteria. These syringol compounds have sufficient molecular masses to easily pass through the cell membranes. Although the molecular mass of Hs slightly violates Ghose's rule (MW = 154.16), it can be tolerated, as many commercial drugs, such as isoniazid, allopurinol, and metformin, have molecular masses < 150 g mol^−1^.^101^

**Table 7:** Physicochemical parameter of investigated syringol derivatives and relevant drugs

	Hs	HAs	HPns	HPs	Ascorbic acid	Melatonin
log *P*	1.32	2.28	2.30	2.43	−1.42	1.83
PSA	38.69	38.69	38.69	38.69	107.22	54.12
^X^At	11	14	14	14	12	17
MW	154.16	194.23	194.23	196.24	176.12	232.28
HB^A^	3	3	3	3	6	2
HB^B^	1	1	1	1	4	2
RB	2	4	3	4	2	5
^M^ *R*	41.45	55.56	56.35	56.03	35.12	67.18
LD_50_ (mg kg^−1^)	549.58	3102.38	2849.01	2653.71	11 908.53	2393.69
*M*	0.54 (+)	0.11 (−)	0.41 (−)	0.45 (−)	−0.01 (−)	0.08 (−)

The cell permeability of syringol derivatives can also be predicted from the values of lipophilicity, polar surface area, and molar refractivity. All syringol derivatives have log *P* values in the range of −0.4 to 5.0, indicating good solubility in both polar and non-polar environments. The number of hydrogen donors and acceptors, as well as the total number of elements in the compounds, also affect these properties.

The interesting part is the moderately lipophilic nature and the polar surface area of syringol derivatives (0.4–0.6; <79 Å^2^), which allow them to pass the blood–brain barrier (BBB), as indicated by the BOILED-EGG model.^105^ This ability was also validated by making predictions using another program called LightBBB, which yielded similar results.^106^ The ability to pass the membrane is known as a requirement for the development of neuroprotective compounds.^107^

To gain an understanding of the potential hazards of syringol derivatives on the body, the LD_50_ and *M* values of commercial antioxidants (ascorbic acid and melatonin) were used as comparative data. Substituted syringols are known to have higher LD_50_ values than melatonin, while Hs has an LD_50_ value of 549.58 mg kg^−1^, which is significantly below the reference values. Other studies have also reported that syringol shows toxicity effects *in vitro* and *in vivo* in the concentration range of 0.5–2 ppm.^108^ Additionally, Hs exhibits potential to induce mutations in the body (*M* = 0.53), which is not observed in other syringol derivatives. Considering all these factors, apart from their potential as safe antioxidants, compounds like HAs, HPns, and HPs can be further investigated as specific neuroprotective agents.

## Conclusions

Kinetic and thermodynamic studies using the M06-2X/6-311++G(d,p) level of theory of compounds Hs, HAs, HPns, and HPs have demonstrated good antioxidant capacity in both polar and non-polar environments. Among these four compounds, HPs is the most effective as HOO˙ radical scavenger with *k*_overall_ values of 1.75 × 10^8^ M^−1^ s^−1^ in water and 1.13 × 10^8^ M^−1^ s^−1^ in pentyl ethanoate. In pentyl ethanoate, hydrogen transfer at the 1-OH site is the most contributing pathway in all syringol derivatives. Observations of the transition structure at the 1-OH site showed the presence of intermolecular hydrogen bonding, which influences the low activation energy. This interaction is quantified using the Activation Strain Model (ASM), which has been successfully employed for the first time in antioxidant activity research. Furthermore, the ASM analysis can also explain the disfavor of the RAF mechanism by the aromatic ring, as evidenced by the decrease in the interaction energy after the formation of the transition structure. Hydrogen transfer mechanism in syringol derivatives is still a challenge to ensure whether through HT or PCET reactions. Under physiological conditions, HT competes with SPLET reactions (43.4% *vs.* 56.6%) in Hs, with SPLET being the dominant pathway. Ionization effects and p*K*_a_ values contribute to the high radical scavenging capacity of HPns in basic environments. Based on physiochemistry and toxicity parameters prediction, the four compounds exhibited good bioavailability and low toxicity. Only HAs, HPns, and HPs had the potential to be used as bioactive compounds in regard of health. The BBB-permeable properties of these three compounds also provide insights for further research in the therapy of neurodegenerative diseases. Although Hs is toxic to humans, its antioxidant abilities may find applications in other industries.

## Author contributions

Rahmanto Aryabraga Rusdipoetra: investigation, formal analysis, visualization, writing – original draft, data curation. Hery Suwito: validation, writing – review & editing. Ni Nyoman Tri Puspaningsih: validation, writing – review & editing. Kautsar Ul Haq: conceptualization, methodology, investigation, formal analysis, supervision, project administration.

## Conflicts of interest

The authors declare that they have no known competing financial interests or personal relationships that could have appeared to influence the work reported in this paper.

## Supplementary Material

RA-014-D3RA08346B-s001
