# Supplementary material for: Theoretical insight of reactive oxygen species scavenging mechanism in lignin waste depolymerization products
Source: RSC Adv. 2024 Feb 20;14(9):6310–23. doi: 10.1039/d3ra08346b (PMC10877321; doi:10.1039/d3ra08346b)
Supplement: RA-014-D3RA08346B-s001 [file RA-014-D3RA08346B-s001.pdf]

Electronic Supplementary Material (ESI) for RSC Advances.
This journal is © The Royal Society of Chemistry 2024

Suplementary Information

Theoretical Insight of Reactive Oxygen Species Scavenging Mechanism in Lignin Waste Depolymerization Products

Rahmanto Aryabraga Rusdipoetra[1,2], Hery Suwito[2], Ni Nyoman Tripuspaningsih[2,3], and Kautsar Ul Haq[1,2, a)]

Author Affiliations
[1]*Bioinformatic Research Group, Research Centre of Bio-Molecule Engineering (BIOME), Airlangga University, Jl. Ir. H. Soekarno Mulyorejo, Surabaya, Indonesia*
[2]*Department of Chemistry, Faculty of Science and Technology, Airlangga University, Jl. Ir. H. Soekarno Mulyorejo, Surabaya, Indonesia*
[3]*Proteomic Research Group, Research Centre of Bio-Molecule Engineering (BIOME), Airlangga University, Jl. Ir. H. Soekarno Mulyorejo, Surabaya, Indonesia*

Author Emails
[a)] *Corresponding author: kautsar.ul.haq@fst.unair.ac.id*

Table of Contents

1. Figure S1: Intrinsic Reaction Coordinate of HT and RAF Mechanism in Water........1
2. Figure S2: Intrinsic Reaction Coordinate of HT and RAF Mechanism in Pentyl Ethanoate........6
3. Figure S3: Cartesian Coordinate and Thermochemical Values of all Optimized Stationary Points in Water........9
4. Figure S4: Cartesian Coordinate and Thermochemical Values of all Optimized Stationary Points in Pentyl Ethanoate........49
5. Figure S5: SOMO and HOMO density surfaces of the HT/PCET transition states, in aqueous solution, computed with isodensity value of 0.02 au........72
6. Figure S6. Boiled-Egg model and LightBBB prediction of studied syringol derivatives........73

1. Figure S1: Intrinsic Reaction Coordinate of HT and RAF Mechanism in Water

1a. IRC plot of HT TS 1-OH···•OOH in Hs

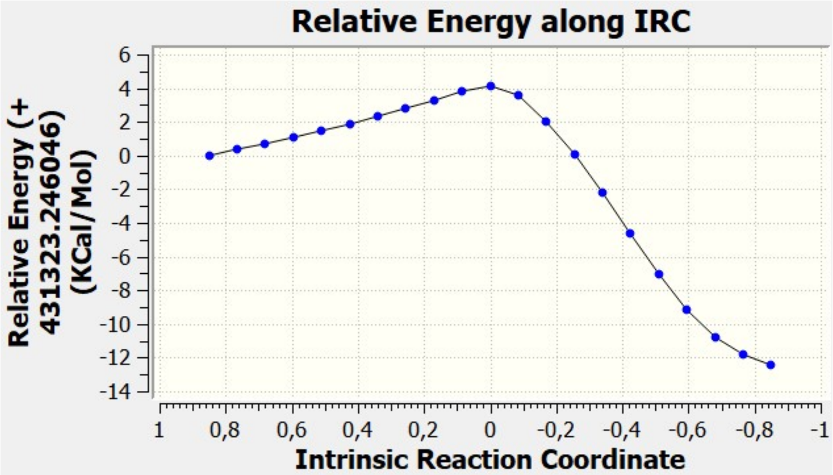

1b. IRC plot of HT TS 1-OH···•OOH in HAs

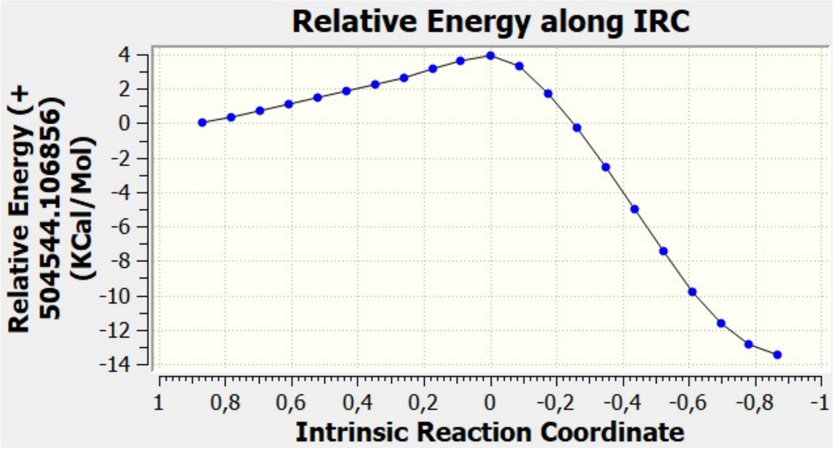

1c. IRC plot of HT TS 1’-CH···•OOH in HAs

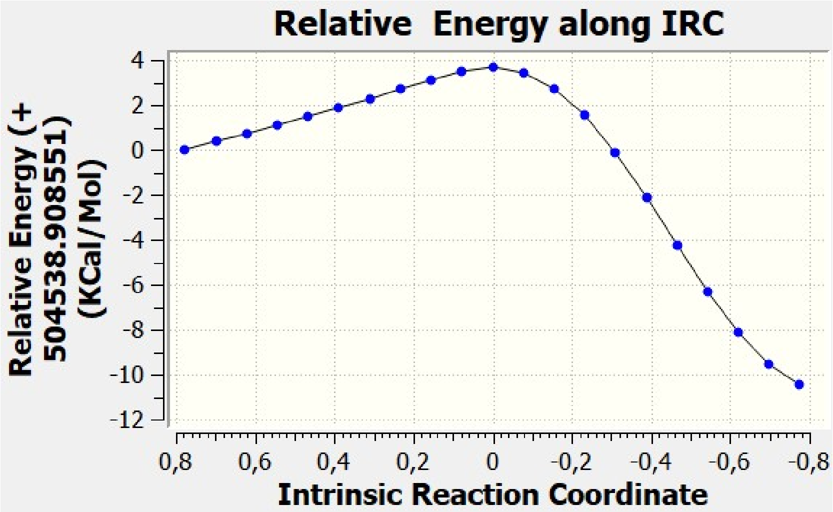

1d. IRC plot of HT TS 1’-CH···•OOH in anionic HAs

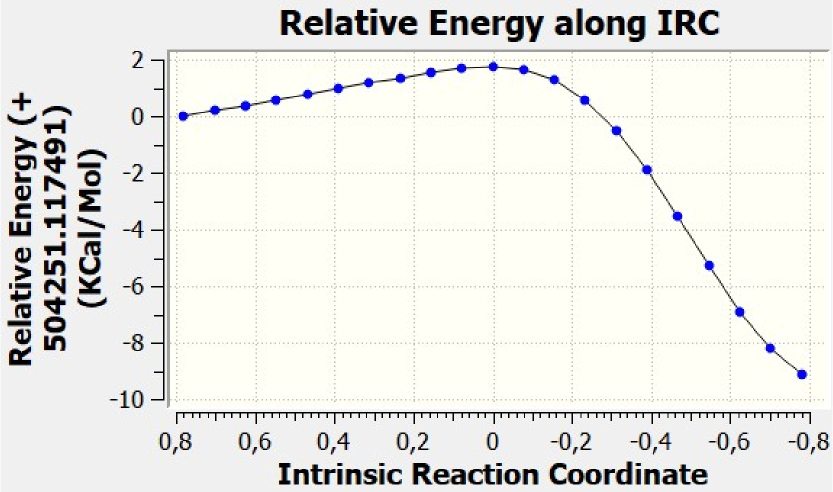

1e. IRC plot of RAF TS 2’-C···•OOH in HAs

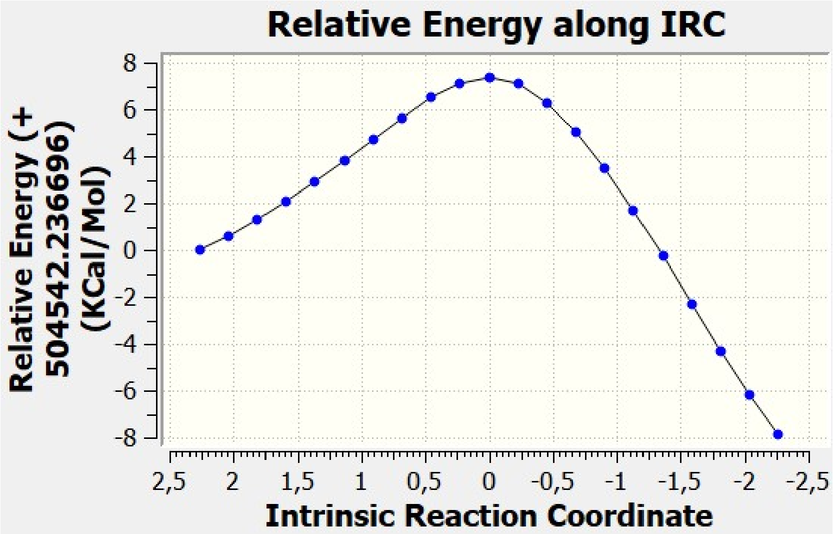

1f. IRC plot of RAF TS 2'-C···•OOH in anionic HAs

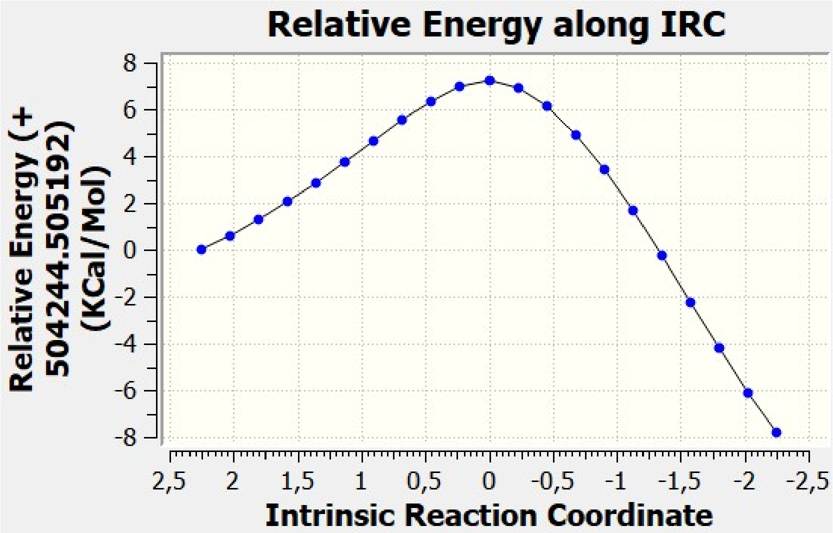

1g. IRC plot of RAF TS 3'-C···•OOH in HAs

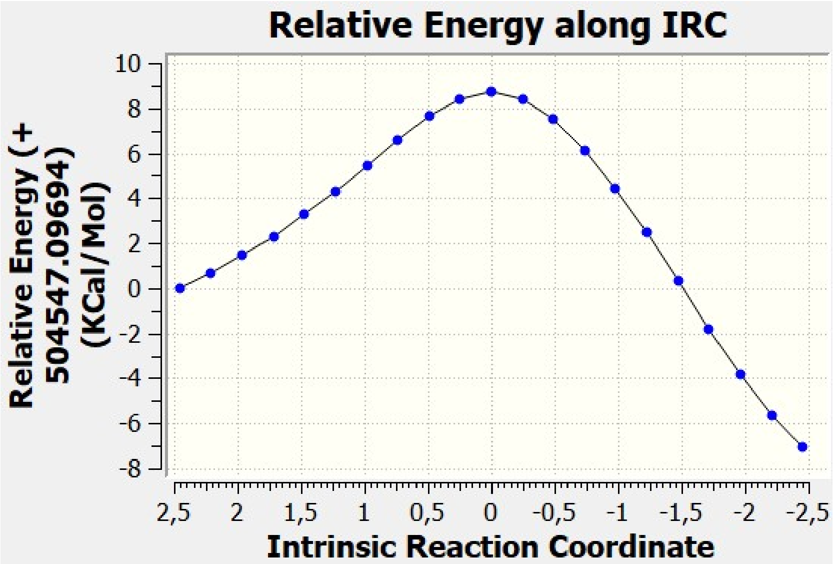

1h. IRC plot of HT TS 1-OH···•OOH in HPns

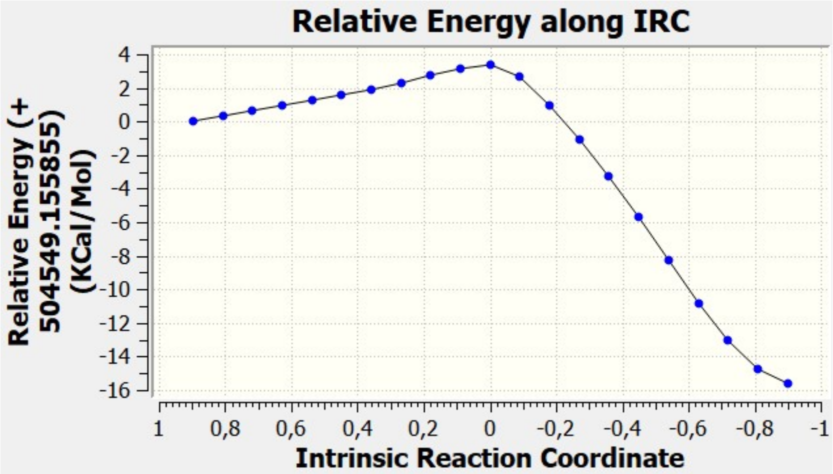

1i. IRC plot of HT TS 3’-CH···•OOH in HPns

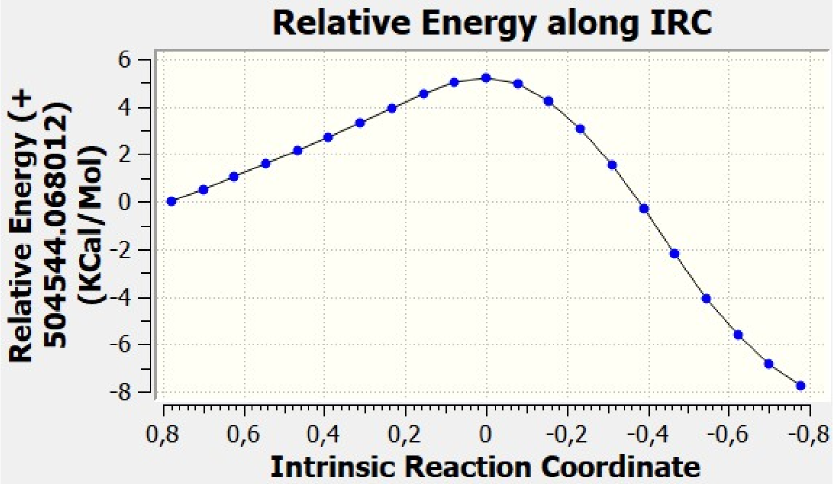

1j. IRC plot of HT TS 3’-CH···•OOH in anionic HPns

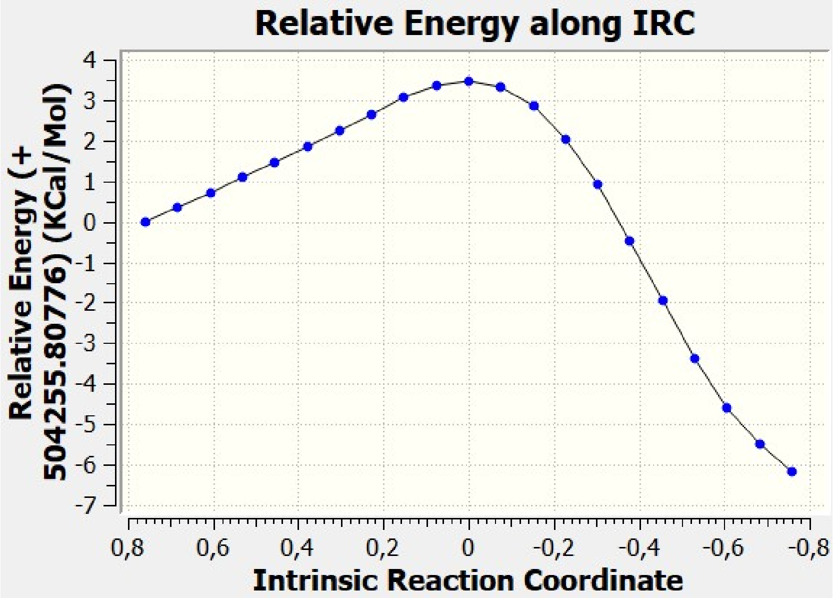

1k. IRC plot of RAF TS 2’-C···•OOH in HPns

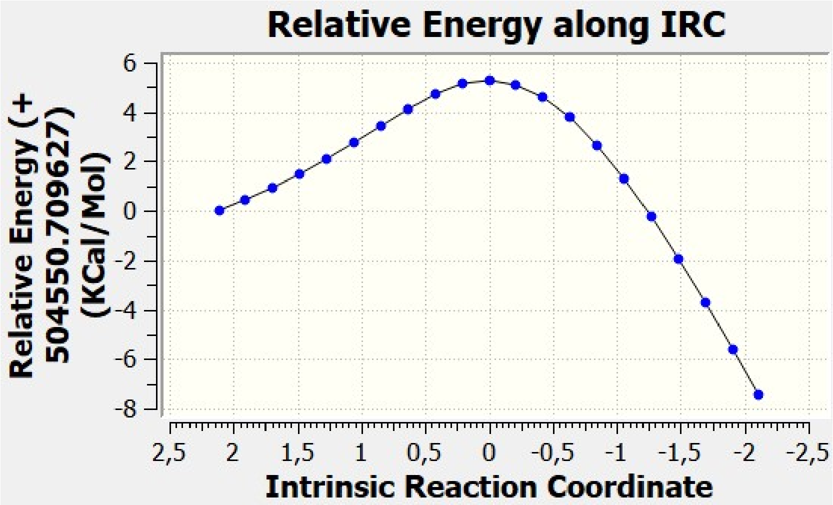

1l. IRC plot of RAF TS 2’-C···•OOH in anionic HPns

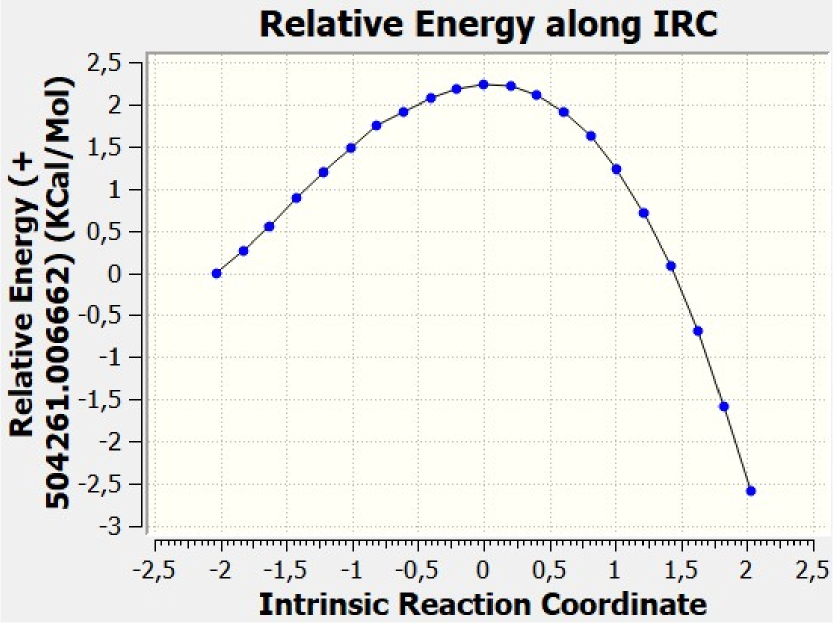

1m. IRC plot of HT TS 1-OH···•OOH in HPs

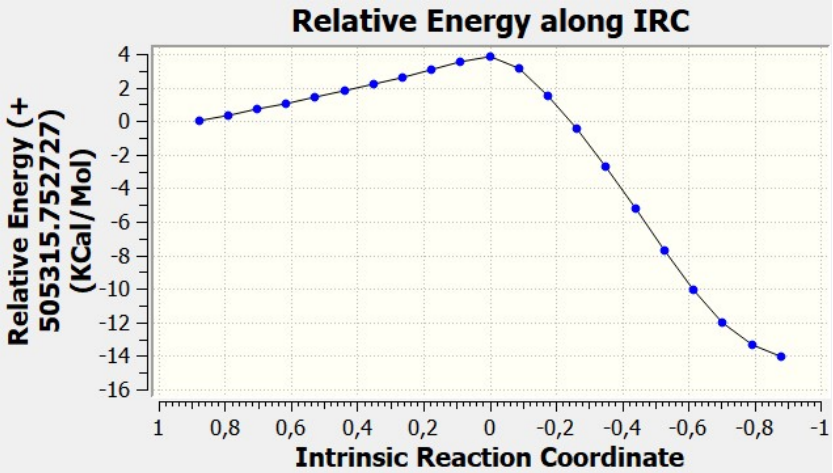

1n. IRC plot of HT TS 1’-CH···•OOH in HPs

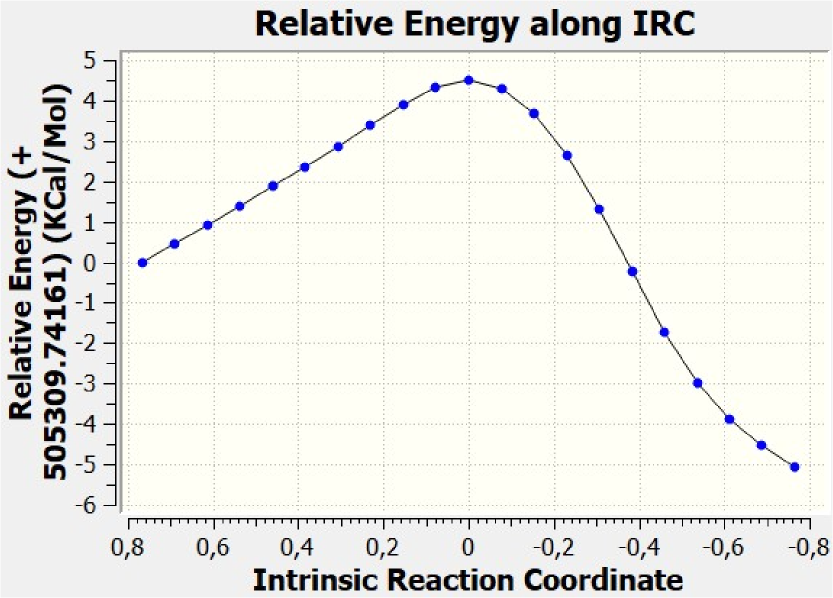

1o. IRC plot of HT TS 1’-CH⋯•OOH in anionic HPs

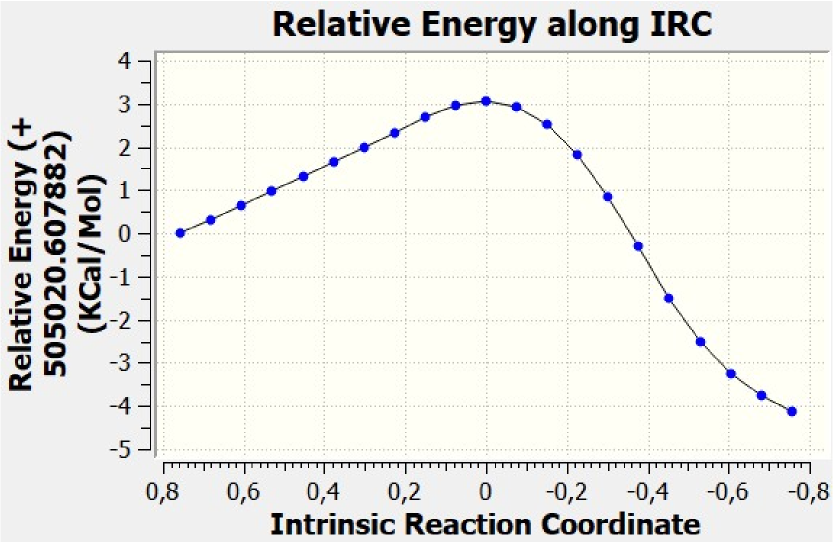

2. **Figure S2: Intrinsic Reaction Coordinate of HT and RAF Mechanism in Pentyl Ethanoate**

2a. IRC plot of HT TS 1-OH⋯•OOH in Hs

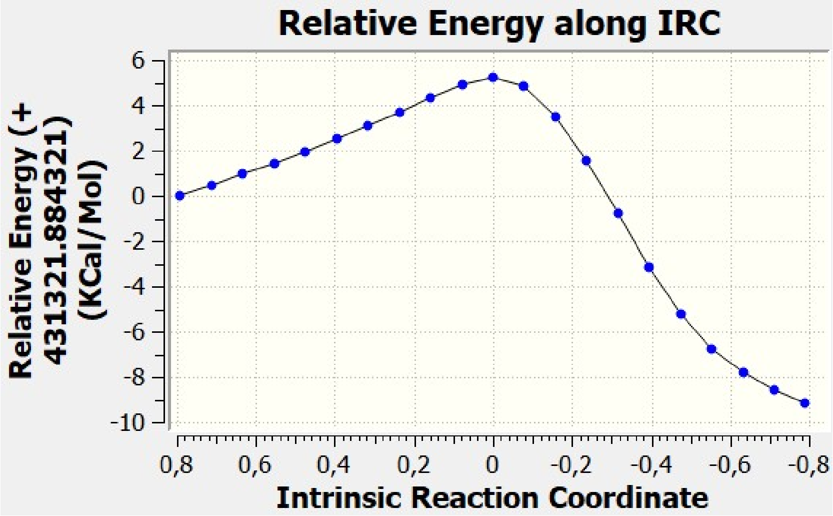

2b. IRC plot of HT TS 1-OH⋯•OOH in HAs

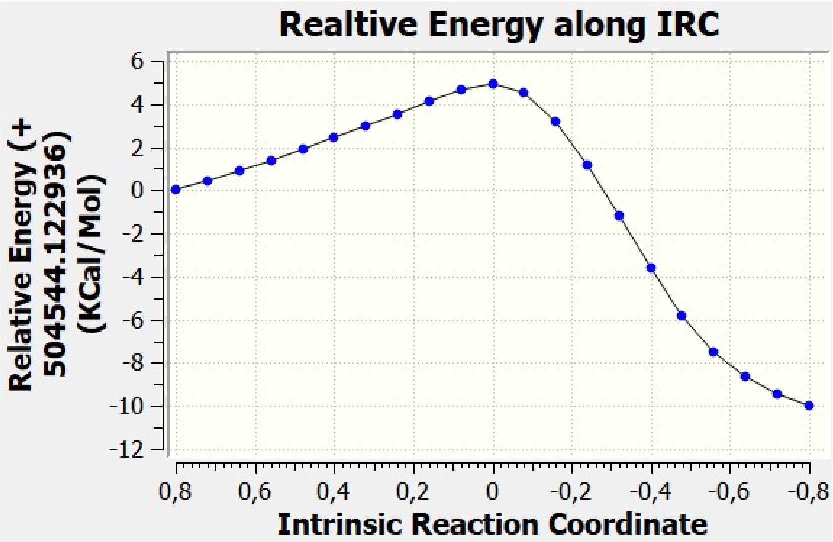

2c. IRC plot of HT TS 1’-CH···•OOH in HAs

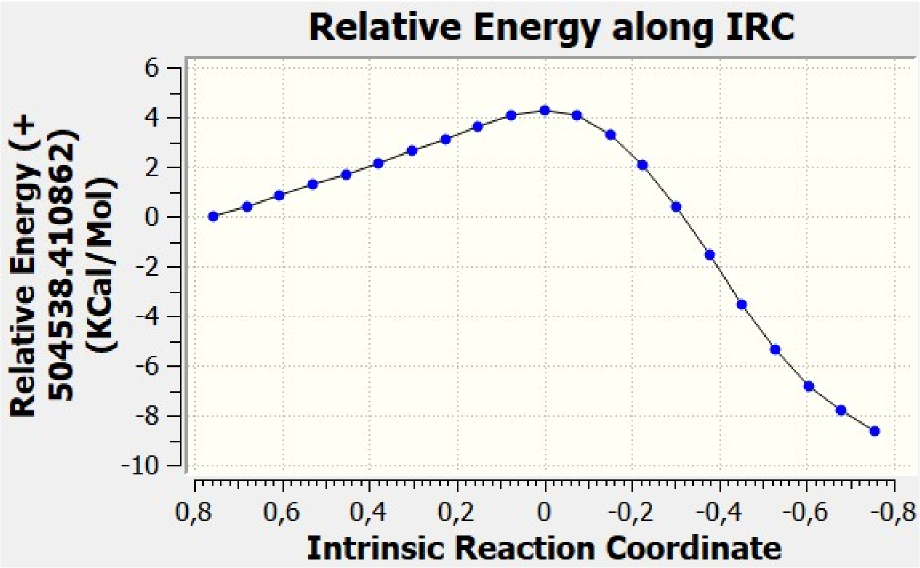

2d. IRC plot of HT TS 1-OH···•OOH in HPns

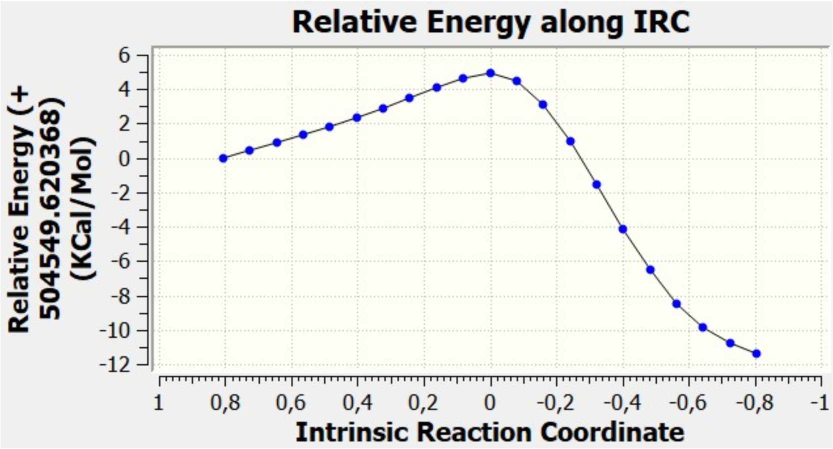

2e. IRC plot of HT TS 3’-CH···•OOH in HPns

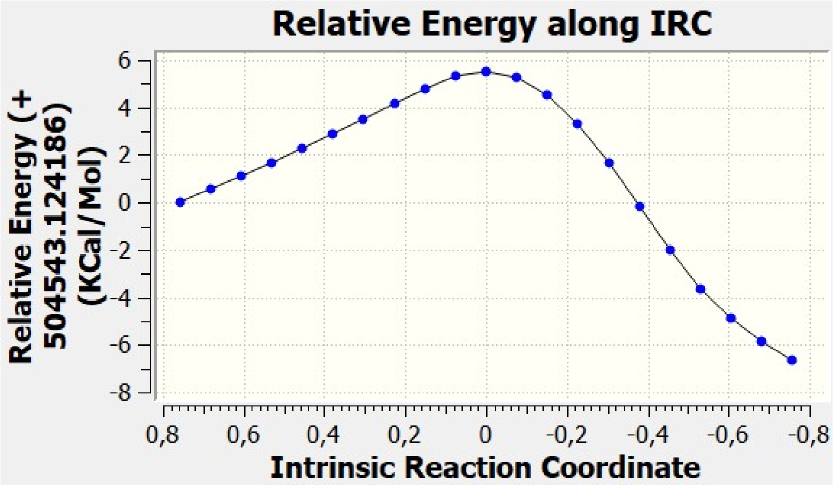

2f. IRC plot of RAF TS 2’-C···•OOH in HPns

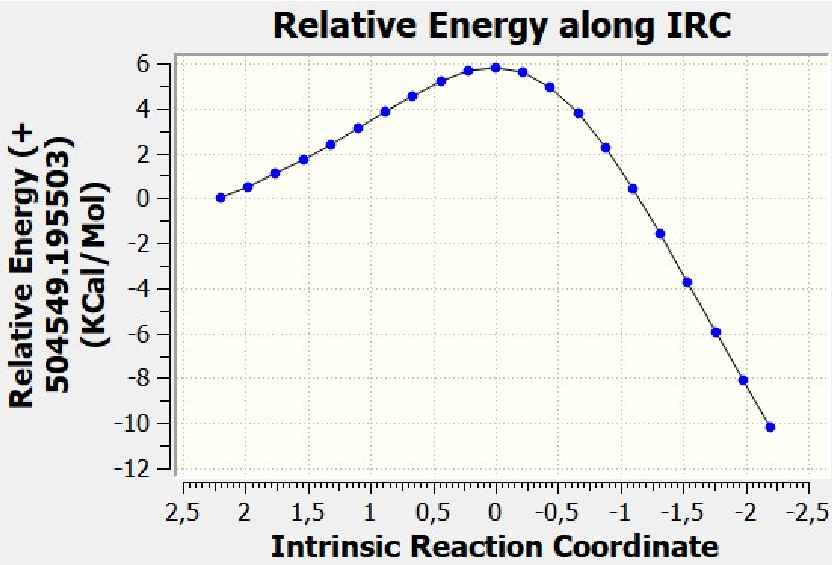

2g. IRC plot of HT TS 1-OH···•OOH in HPs

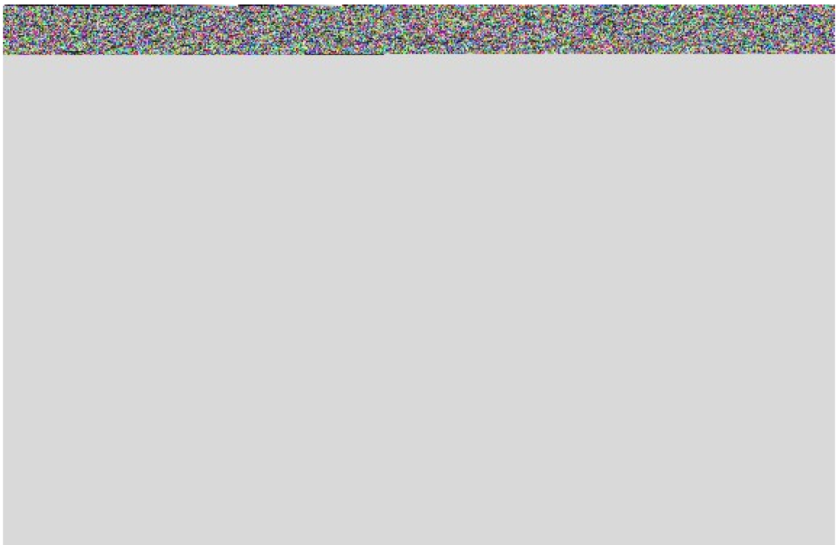

2h. IRC plot of HT TS 1’-CH···•OOH in HPs

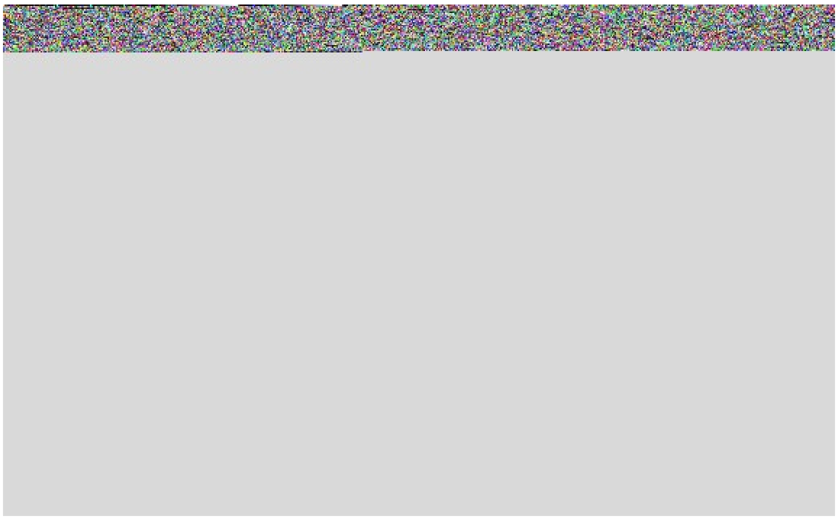

3. Figure S3: Cartesian Coordinate and Thermochemical Values of all Optimized Stationary Points in Water

Name	Hs (Syringol)
Cartesian Coordinate:	Thermochemical Values:
O 2.32777200 -0.79878600 -0.00030400<br>O -2.31602600 -0.75685100 -0.00053900<br>O 0.00640000 -2.05358500 -0.00069000<br>C 0.02993100 2.08702600 -0.00031400<br>C 1.21856400 -0.00499500 -0.00043000<br>C -1.19980300 0.02960100 -0.00051800<br>C -1.19257300 1.42267800 -0.00037700<br>C 1.23431500 1.39010200 -0.00033700<br>C -0.00038900 -0.68418600 -0.00059500<br>C 3.59304500 -0.14409800 0.00143100<br>C -3.57965500 -0.09738700 0.00146900<br>H -2.11832600 1.98152900 -0.00029900<br>H 2.17090300 1.93013300 -0.00026200<br>H -0.91159800 -2.35969300 -0.00048700<br>H 3.71005500 0.47156700 0.89627900<br>H 3.71213200 0.47243900 -0.89253800<br>H 4.33798200 -0.93652600 0.00188600<br>H -3.69290200 0.51957400 -0.89267900<br>H -3.69063500 0.51832700 0.89676500<br>H -4.32832100 -0.88603700 0.00184200<br>H 0.04493000 3.17001800 -0.00021800	• Electronic Energy<br>= -536.458220<br>• Zero Point Energy Coorection<br>= 0.170771<br>• Thermal Correction to Energy<br>= 0.181536<br>• Thermal Correction to Enthalpy<br>= 0.182480<br>• Thermal Correction to Free Energy<br>= 0.133217

Name	Hs 1-OH radical
Cartesian Coordinate:	Thermochemical Values:
O 2.33973300 -0.78896300 0.00001200<br>O -2.33974500 -0.78895400 0.00000200<br>O 0.00000800 -2.03614600 0.00002000<br>C 0.00002600 2.02547300 0.00000000<br>C 1.24384100 -0.02265800 0.00001100<br>C -1.24384300 -0.02265200 0.00000500<br>C -1.23003800 1.35755800 -0.00000200<br>C 1.23008700 1.35755700 0.00000600<br>C -0.00004600 -0.78866100 0.00001300<br>C 3.60906400 -0.13154200 -0.00002000<br>C -3.60907400 -0.13153200 -0.00002700<br>H -2.14759700 1.92898000 -0.00000800<br>H 2.14765900 1.92894900 0.00000600<br>H 3.71712700 0.48342300 0.89542400<br>H 3.71709000 0.48340200 -0.89548300<br>H 4.35204100 -0.92458700 -0.00002500<br>H -3.71709400 0.48341700 -0.89548800<br>H -3.71712600 0.48343600 0.89541600<br>H -4.35205800 -0.92457000 -0.00003200<br>H -0.00010900 3.10879600 -0.00000400	• Electronic Energy<br>= -535.822999<br>• Zero Point Energy Coorection<br>= 0.158473<br>• Thermal Correction to Energy<br>= 0.168814<br>• Thermal Correction to Enthalpy<br>= 0.169758<br>• Thermal Correction to Free Energy<br>= 0.121775

Name	Hs HT 1-OH···•OOH Transition State
Cartesian Coordinate:	Thermochemical Values:

O -2.15687000 -0.13055600 -0.69042700<br>O 2.42551700 0.64660400 -0.43123500<br>C 0.49251600 -2.28652400 0.63358400<br>C -0.97276700 -0.65395100 -0.32057800<br>C 1.43699300 -0.23318600 -0.17566400<br>C 1.60932700 -1.47868500 0.41330300<br>C -0.79542500 -1.89470500 0.28284000<br>C 0.14117800 0.19228800 -0.56717600<br>C -3.33630500 -0.86384800 -0.35413400<br>C 3.75142900 0.26991400 -0.05965900<br>H 2.59008000 -1.82459100 0.70793800<br>H -1.63543100 -2.54534900 0.48095500<br>H -3.39422600 -1.01496800 0.72609800<br>H -3.34917200 -1.82574600 -0.87010200<br>H -4.16952500 -0.25222000 -0.69026000<br>H 4.05959000 -0.62809300 -0.59916800<br>H 3.81451100 0.10132300 1.01738500<br>H 4.38551600 1.10683500 -0.34083900<br>H 0.63532900 -3.25442400 1.09916800<br>O -0.02526700 1.36241200 -1.17451500<br>H -0.57725300 2.01088500 -0.53190600<br>O -1.12157800 2.58635100 0.57904700<br>O -0.94273000 1.62679800 1.51568200<br>H -1.75366700 1.08566500 1.49722100	• Electronic Energy<br>= -687.350732<br>• Zero Point Energy Coorection<br>= 0.183186<br>• Thermal Correction to Energy<br>= 0.196561<br>• Thermal Correction to Enthalpy<br>= 0.197505<br>• Thermal Correction to Free Energy<br>= 0.142245

Name	Hs HT 1-OH···•OOH Reactant Complex
Cartesian Coordinate:	Thermochemical Values:
O -1.81551200 -0.59761300 -0.39242100<br>O 2.44742600 1.29096100 -0.19494800<br>C 1.39435000 -2.17382900 0.49568800<br>C -0.48606700 -0.82605800 -0.12925200<br>C 1.71656700 0.15335200 -0.02180000<br>C 2.24271400 -1.07812300 0.36126600<br>C 0.03130300 -2.06122800 0.25398400<br>C 0.34451500 0.28542200 -0.26886000<br>C -2.64203700 -1.74342900 -0.63631300<br>C 3.85259600 1.19869800 0.02136900<br>H 3.30097300 -1.18762600 0.55390700<br>H -0.61709400 -2.92026300 0.36031600<br>H -2.77540700 -2.32376500 0.27793700<br>H -2.19991400 -2.36150000 -1.41905300<br>H -3.60068600 -1.34953000 -0.96652700<br>H 4.30447200 0.48067400 -0.66695900<br>H 4.06843900 0.91168600 1.05315600<br>H 4.24904000 2.19258700 -0.17299200<br>H 1.80495800 -3.13028100 0.79460400<br>O -0.11939100 1.50729600 -0.66524200<br>H -1.08959400 1.54290500 -0.61180700<br>O -2.75648500 2.19634100 0.21267400<br>O -3.18722300 1.25138400 0.99579000<br>H -2.71935300 0.42933800 0.69410200	• Electronic Energy<br>= -687.372444<br>• Zero Point Energy Coorection<br>= 0.188629<br>• Thermal Correction to Energy<br>= 0.202623<br>• Thermal Correction to Enthalpy<br>= 0.203567<br>• Thermal Correction to Free Energy<br>= 0.146700

Name	Hs HT 1-OH···•OOH Product Complex
Cartesian Coordinate:	Thermochemical Values:
O -1.80199700 -0.71541300 -0.27306600<br>O 2.39332800 1.34998100 -0.10531500<br>C 1.51109000 -2.17970600 0.25569800<br>C -0.48706300 -0.90996600 -0.11038500<br>C 1.74362700 0.18745200 -0.02047500<br>C 2.32609900 -1.04566000 0.19748500<br>C 0.11888100 -2.12856500 0.10415700<br>C 0.29849200 0.31691500 -0.18632900<br>C -2.65537000 -1.86610700 -0.27003300<br>C 3.81636400 1.33085900 0.03951100<br>H 3.39503300 -1.14743800 0.32166400<br>H -0.45890500 -3.04056100 0.15858800<br>H -2.59685300 -2.37743400 0.69231000<br>H -2.37479400 -2.54258100 -1.07891400<br>H -3.66032600 -1.48428300 -0.43172100<br>H 4.26718700 0.71717000 -0.74266400<br>H 4.09046600 0.95025300 1.02512500<br>H 4.13623800 2.36402100 -0.06441900<br>H 1.97648100 -3.14293800 0.42613500<br>O -0.22860400 1.43313500 -0.38636600<br>H -1.94388100 1.89055900 -0.53014000<br>O -2.89573000 2.02003900 -0.33164600<br>O -2.97931000 1.53580500 1.00471100<br>H -2.76486900 0.59351700 0.89970200	• Electronic Energy<br>= -687.386322<br>• Zero Point Energy Coorection<br>= 0.188283<br>• Thermal Correction to Energy<br>= 0.202466<br>• Thermal Correction to Enthalpy<br>= 0.203410<br>• Thermal Correction to Free Energy<br>= 0.145989

Name	Hs RAF 1-C···•OOH Product
Cartesian Coordinate:	Thermochemical Values:
O 2.34088600 -0.52654900 -0.37260100<br>O -2.34533300 -0.51581400 -0.30927800<br>O 0.00418200 -1.30956100 -1.48517000<br>C 0.00989000 2.26558000 0.24049600<br>C 1.24828600 0.24608600 -0.19664100<br>C -1.25027600 0.25628300 -0.14300700<br>C -1.22626200 1.59416800 0.11870100<br>C 1.23498800 1.58413800 0.07545400<br>C 3.61261800 0.11234400 -0.28134600<br>C -3.61693100 0.12806700 -0.24545600<br>H -2.14868500 2.14985800 0.22567600<br>H 2.16302800 2.13415300 0.16001000<br>H -0.88241800 -1.67573200 -1.61927900<br>H 3.74894100 0.54896100 0.71065700<br>H 3.70631300 0.88643000 -1.04634200<br>H 4.35160100 -0.66710300 -0.44871500<br>H -3.69281900 0.89481500 -1.01969300<br>H -3.76635700 0.57455400 0.73988200<br>H -4.35598100 -0.65028200 -0.41689600<br>H 0.01867500 3.32574700 0.45453200<br>C -0.00595100 -0.57142200 -0.29406900<br>O 0.01033600 -1.63133000 0.71297300<br>O -0.02215100 -1.04466500 2.00072400<br>H 0.91616700 -0.98950300 2.24220000	• Electronic Energy<br>= -687.365682<br>• Zero Point Energy Coorection<br>= 0.188927<br>• Thermal Correction to Energy<br>= 0.202302<br>• Thermal Correction to Enthalpy<br>= 0.203246<br>• Thermal Correction to Free Energy<br>= 0.148747

Name	Anionic Hs RAF 1-C···•OOH Product
Cartesian Coordinate:	Thermochemical Values:
O 2.35583100 -0.50508300 -0.35734800<br>O -2.37517400 -0.52700400 -0.32300700<br>O -0.01850100 -1.21525300 -1.62005600<br>C -0.01847300 2.24147100 0.29062900<br>C 1.23683700 0.24346600 -0.19414500<br>C -1.26114200 0.22917400 -0.17088800<br>C -1.24790900 1.55905700 0.14362600<br>C 1.21577600 1.57265900 0.12283300<br>C 3.61036900 0.14658100 -0.19688500<br>C -3.63323600 0.11598400 -0.15548800<br>H -2.17233700 2.10581500 0.28089000<br>H 2.13673600 2.12869000 0.24561400<br>H 3.70599700 0.55718300 0.81147900<br>H 3.72637900 0.94535300 -0.93362300<br>H 4.36970200 -0.61549600 -0.35696100<br>H -3.75850500 0.91402900 -0.89153400<br>H -3.72664900 0.52569400 0.85331600<br>H -4.38812000 -0.65125700 -0.31182900<br>H -0.02202000 3.29537900 0.53600400<br>C -0.01026100 -0.58614200 -0.44887800<br>O 0.00857800 -1.71105700 0.56861600<br>O 0.00761700 -1.19546100 1.89651300<br>H 0.95025000 -1.10803500 2.10407000	• Electronic Energy<br>= -686.891328<br>• Zero Point Energy Coorection<br>= 0.175413<br>• Thermal Correction to Energy<br>= 0.188421<br>• Thermal Correction to Enthalpy<br>= 0.189365<br>• Thermal Correction to Free Energy<br>= 0.135614

Name	Hs RAF 2-C···•OOH Product
Cartesian Coordinate:	Thermochemical Values:
O 1.83012500 -0.59648500 -1.10243300<br>O -2.59203600 -0.71419400 -0.19697500<br>O -0.28011100 -2.02187700 -0.13051200<br>C -0.16816500 2.10802500 -0.04627700<br>C -1.46344900 0.05560900 -0.14252200<br>C -1.43307800 1.44238400 -0.11131500<br>C 1.00864400 1.44475000 -0.02349900<br>C -0.28420700 -0.67711600 -0.11564800<br>C 3.08166000 0.04134900 -1.38130600<br>C -3.84134300 -0.03088500 -0.26419600<br>H -2.34406900 2.02332900 -0.12987700<br>H 1.95234300 1.97140000 0.05184700<br>H -1.19479300 -2.34156500 -0.17763700<br>H 3.66062000 0.19307700 -0.46893300<br>H 2.92505500 0.99463900 -1.88917100<br>H 3.61798700 -0.63685700 -2.04192600<br>H -3.88821200 0.59665800 -1.15707800<br>H -3.99548900 0.57808600 0.62928700<br>H -4.60278700 -0.80532800 -0.31743600<br>H -0.16785600 3.19156100 -0.01202500<br>C 1.07061600 -0.04743200 -0.05571400<br>O 1.78680100 -0.55650000 1.11904600<br>O 1.09477800 -0.16947800 2.29039700<br>H 1.49667300 0.68316800 2.51961600	• Electronic Energy<br>= -687.355834<br>• Zero Point Energy Coorection<br>= 0.188331<br>• Thermal Correction to Energy<br>= 0.201888<br>• Thermal Correction to Enthalpy<br>= 0.202832<br>• Thermal Correction to Free Energy<br>= 0.147965

Name	Anionic Hs RAF 2-C···•OOH Product
Cartesian Coordinate:	Thermochemical Values:
O 1.88078900 -0.64560500 -1.05582000<br>O -2.61610500 -0.77073400 -0.20430200<br>O -0.27594400 -2.05366200 -0.10477500<br>C -0.22076900 2.06150400 -0.10564800<br>C -1.47281900 -0.00679600 -0.15969400<br>C -1.46952800 1.37763000 -0.14876800<br>C 0.97644200 1.41843900 -0.08333200<br>C -0.27912000 -0.77853200 -0.11847900<br>C 3.10478800 0.03030500 -1.34272200<br>C -3.85363200 -0.07644800 -0.25607000<br>H -2.38737600 1.94722600 -0.17328300<br>H 1.90665700 1.97267200 -0.03248400<br>H 3.66344700 0.25016100 -0.43068800<br>H 2.92171100 0.95464000 -1.89509600<br>H 3.68541400 -0.64935600 -1.96437000<br>H -3.91542100 0.54948700 -1.15038200<br>H -3.99446600 0.54256400 0.63402800<br>H -4.62771600 -0.84056700 -0.29303600<br>H -0.23513500 3.14670700 -0.09609100<br>C 1.05905100 -0.06408500 -0.06467700<br>O 1.78262700 -0.50921200 1.15692100<br>O 1.06583700 -0.08567500 2.30203600<br>H 1.21876800 0.87346900 2.32525800	• Electronic Energy<br>= -686.897953<br>• Zero Point Energy Coorection<br>= 0.176219<br>• Thermal Correction to Energy<br>= 0.189254<br>• Thermal Correction to Enthalpy<br>= 0.190198<br>• Thermal Correction to Free Energy<br>= 0.136287

Name	Hs RAF 3-C···•OOH Product
Cartesian Coordinate:	Thermochemical Values:
O -1.91073500 1.38912000 0.12358800<br>O 2.68786600 0.63352700 0.18443000<br>O 0.59245000 2.21624100 0.57553200<br>C 0.03772500 -1.64035200 -0.95668000<br>C -0.88633800 0.53702400 -0.17481000<br>C 1.49283700 0.08843800 -0.13483800<br>C 1.29771300 -1.19652100 -0.67935000<br>C 0.38162200 0.95263000 0.08809600<br>C -3.10482300 1.26247200 -0.65428000<br>C 3.86292700 -0.14569300 -0.04460900<br>H 2.15243200 -1.82145500 -0.90517900<br>H 1.54596700 2.36220300 0.64804600<br>H -3.61825200 0.32397100 -0.43778500<br>H -2.87503000 1.32920600 -1.72017400<br>H -3.73795100 2.09782200 -0.36368800<br>H 3.96419500 -0.37735500 -1.10656000<br>H 3.82802800 -1.06596600 0.54149000<br>H 4.69602800 0.47122100 0.28205100<br>H -0.11766000 -2.61653700 -1.39947100<br>C -1.18184500 -0.85797300 -0.61162000<br>O -1.97325000 -1.54978700 0.41768400<br>O -1.22855300 -1.61659800 1.62380100<br>H -0.67281900 -2.40281200 1.50338200<br>H -1.90606500 -0.88047800 -1.43383800	• Electronic Energy<br>= -687.353355<br>• Zero Point Energy Coorection<br>= 0.188731<br>• Thermal Correction to Energy<br>= 0.202344<br>• Thermal Correction to Enthalpy<br>= 0.203288<br>• Thermal Correction to Free Energy<br>= 0.148376

Name	Anionic Hs RAF 3-C···•OOH Product
Cartesian Coordinate:	Thermochemical Values:
O -1.96858700 1.32925100 0.20261100<br>O 2.70900800 0.69543100 0.19112700<br>O 0.58473000 2.22845700 0.67945000<br>C 0.08413200 -1.59030000 -0.97636900<br>C -0.87442900 0.54621300 -0.14492400<br>C 1.49994600 0.16532500 -0.12097500<br>C 1.33326400 -1.10929000 -0.69285800<br>C 0.37357500 1.04682400 0.16442600<br>C -3.06028300 1.31126200 -0.71128700<br>C 3.87059500 -0.09114700 -0.05384300<br>H 2.19985000 -1.71117400 -0.93754300<br>H -3.59996400 0.36110500 -0.67253100<br>H -2.71324800 1.49435400 -1.73261200<br>H -3.73188000 2.11311500 -0.40763800<br>H 3.97406100 -0.30966200 -1.11922300<br>H 3.83416700 -1.02315300 0.51499300<br>H 4.71378600 0.50935300 0.27992100<br>H -0.04477700 -2.56267600 -1.43649100<br>C -1.14336600 -0.83338700 -0.61712100<br>O -1.94169900 -1.59137700 0.38244200<br>O -1.21793100 -1.69586000 1.59952200	• Electronic Energy<br>= -686.879799<br>• Zero Point Energy Coorection<br>= 0.175859<br>• Thermal Correction to Energy<br>= 0.188989<br>• Thermal Correction to Enthalpy<br>= 0.189933<br>• Thermal Correction to Free Energy<br>= 0.135619

H -0.58569800 -2.41104900 1.42607900<br>H -1.87107600 -0.86042200 -1.43845500	

Name	Hs RAF 4-C···•OOH Product
Cartesian Coordinate:	Thermochemical Values:
O 2.34010900 -1.26558800 0.09315100<br>O -2.30333200 -1.25361600 0.05081100<br>O 0.02020600 -2.38535100 0.60118200<br>C 1.25461900 -0.48258600 -0.15260500<br>C -1.21954000 -0.46409300 -0.18103900<br>C -1.23728800 0.80275600 -0.66413300<br>C 1.28296100 0.78748000 -0.63015400<br>C 0.01004400 -1.12695300 0.11634200<br>C 3.61694700 -0.68339400 -0.15257500<br>C -3.58626500 -0.67783500 -0.18342500<br>H -2.16288000 1.30563800 -0.91074700<br>H 2.21545300 1.28818400 -0.85224800<br>H -0.89530000 -2.68697700 0.70926900<br>H 3.76470400 0.19977000 0.47346600<br>H 3.72075600 -0.41307100 -1.20614200<br>H 4.34756700 -1.44611200 0.10594200<br>H -3.69538500 -0.40368200 -1.23501900<br>H -3.73020600 0.20132100 0.44875700<br>H -4.31089800 -1.44547300 0.07643500<br>C 0.02986100 1.57678800 -0.80563700<br>O 0.01947400 2.72926600 0.10813700<br>H 0.04678300 2.11800100 -1.75797000<br>O 0.01331600 2.27236400 1.45147300<br>H -0.92682600 2.11282000 1.62957900	• Electronic Energy = -687.358296<br>• Zero Point Energy Coorection = 0.188770<br>• Thermal Correction to Energy = 0.202303<br>• Thermal Correction to Enthalpy = 0.203247<br>• Thermal Correction to Free Energy = 0.148158

Name	Anionic Hs RAF 4-C···•OOH Product
Cartesian Coordinate:	Thermochemical Values:
O 2.37299000 -1.22003200 0.08321700<br>O -2.31356300 -1.28469900 0.06100900<br>O 0.04217100 -2.35914300 0.72099800<br>C 1.24652800 -0.47713200 -0.15181100<br>C -1.20589900 -0.51233800 -0.16113200<br>C -1.24365200 0.74068700 -0.69595500<br>C 1.25463000 0.77486300 -0.68324200<br>C 0.02804000 -1.18478000 0.19395000<br>C 3.62238100 -0.61140400 -0.21113600<br>C -3.57586900 -0.71148600 -0.25041200<br>H -2.17461400 1.20783400 -0.98824700<br>H 2.17586400 1.27213100 -0.95558300<br>H 3.75972800 0.30083900 0.37541700<br>H 3.70225500 -0.37718500 -1.27593300<br>H 4.38447200 -1.33920400 0.06011200<br>H -3.64805900 -0.48013300 -1.31627900<br>H -3.74637400 0.19667200 0.33386600<br>H -4.32033800 -1.46065500 0.01117500<br>C -0.00367700 1.54983200 -0.81110200<br>O -0.04023400 2.66491600 0.19355000<br>H -0.00737900 2.17182000 -1.71171000<br>O -0.05437300 2.12626100 1.50816300<br>H -0.91636600 1.68000100 1.55673300	• Electronic Energy = -686.897190<br>• Zero Point Energy Coorection = 0.176820<br>• Thermal Correction to Energy = 0.189623<br>• Thermal Correction to Enthalpy = 0.190568<br>• Thermal Correction to Free Energy = 0.137451

Name	HAs (4-allylsyringol)
Cartesian Coordinate:	Thermochemical Values:

O -0.15653000 2.73924500 0.03093200<br>O -2.81752100 -1.06494900 0.14164200<br>O -2.51545100 1.57916200 0.29042400<br>C 0.83213900 -0.80119300 -0.37078900<br>C 2.07074700 -1.64437700 -0.60106600<br>C -0.41750700 -1.39987300 -0.23180600<br>C 0.95754200 0.58675100 -0.28450400<br>C -0.16705400 1.37873600 -0.06549700<br>C -1.54093200 -0.60327900 -0.01014000<br>C -1.42157900 0.78198500 0.07325900<br>C 3.03604700 -1.53827800 0.54731600<br>C 1.10461000 3.38921500 -0.09790700<br>C -3.01955500 -2.47356300 0.06198600<br>C 4.26959800 -1.05226300 0.44665400<br>H 1.76639800 -2.68696600 -0.72886900<br>H 2.57046900 -1.32736500 -1.52104400<br>H -0.50965300 -2.47627600 -0.29889300<br>H 1.93658600 1.03819500 -0.38873800<br>H 2.66459600 -1.87432100 1.51390600<br>H -3.29419300 1.00926600 0.36099200<br>H 1.54352000 3.19358200 -1.07910700<br>H 1.79077100 3.06369700 0.68752500<br>H 0.90331400 4.45271400 0.00825600<br>H -2.46464300 -2.98762600 0.84998100<br>H -2.71539000 -2.85156000 -0.91659100<br>H -4.08651000 -2.63040400 0.20170600<br>H 4.92593500 -0.98841500 1.30776900<br>H 4.66049400 -0.70534400 -0.50591600	• Electronic Energy<br>= -653.142002<br>• Zero Point Energy Coorection<br>= 0.232194<br>• Thermal Correction to Energy<br>= 0.246727<br>• Thermal Correction to Enthalpy<br>= 0.247671<br>• Thermal Correction to Free Energy<br>= 0.190002

Name	HAs 1-OH radical
Cartesian Coordinate:	Thermochemical Values:
O -0.01170600 2.76651200 0.05383400<br>O -2.86668100 -0.93657400 0.16933700<br>O -2.40902800 1.66967200 0.34722700<br>C 0.74913100 -0.79596400 -0.43072700<br>C 1.93690100 -1.69024700 -0.68561000<br>C -0.52775600 -1.35118900 -0.27057100<br>C 0.96695300 0.59143800 -0.32329200<br>C -0.08854500 1.43381100 -0.06401600<br>C -1.60471200 -0.52846700 -0.00412700<br>C -1.44198900 0.91603200 0.11120300<br>C 2.82462400 -1.75188200 0.53102400<br>C 1.27378400 3.37089600 -0.09829000<br>C -3.13368600 -2.33741100 0.07208300<br>C 4.07688300 -1.30777200 0.56072500<br>H 1.58465600 -2.69285600 -0.93945200<br>H 2.51103800 -1.30027400 -1.53068300<br>H -0.65119800 -2.42250800 -0.36054100<br>H 1.97350600 0.97191000 -0.44674500<br>H 2.37584500 -2.17835500 1.42578800<br>H 1.67097000 3.17274600 -1.09588800<br>H 1.96212800 2.99589300 0.66183200<br>H 1.11645000 4.43793500 0.03505100<br>H -2.57552900 -2.88395600 0.83468200<br>H -2.87365800 -2.70497400 -0.92241600<br>H -4.20144700 -2.44627600 0.24238300<br>H 4.67582100 -1.36739400 1.46287700<br>H 4.54121200 -0.87424100 -0.32048500	• Electronic Energy<br>= -652.509369<br>• Zero Point Energy Coorection<br>= 0.220225<br>• Thermal Correction to Energy<br>= 0.234252<br>• Thermal Correction to Enthalpy<br>= 0.235196<br>• Thermal Correction to Free Energy<br>= 0.177810

Name	HAs 1’-CH radical
Cartesian Coordinate:	Thermochemical Values:

O 2.51664300 1.68209500 0.00005200<br>O 0.85655500 -2.65370100 -0.00003300<br>O 2.88373300 -0.92968900 0.00002000<br>C -1.01707100 0.54277600 -0.00000300<br>C -2.33394900 1.11151300 -0.00002500<br>C -0.78996600 -0.85087400 0.00001000<br>C 0.09106100 1.41771400 0.00000100<br>C 1.38615300 0.92013700 0.00001700<br>C 0.50721100 -1.33487300 0.00000100<br>C 1.60232100 -0.46130200 -0.00000400<br>C -3.55078200 0.39946500 -0.00004100<br>C 2.34601200 3.09666900 -0.00001800<br>C -0.19710200 -3.61343900 0.00003200<br>C -4.77228800 1.00309200 0.00002300<br>H -2.39138600 2.19685100 -0.00006300<br>H -1.62156700 -1.54086500 0.00004600<br>H -0.08857100 2.48489800 0.00001400<br>H -3.52014500 -0.68683300 -0.00005300<br>H 2.85698200 -1.89724900 -0.00036500<br>H 1.80996100 3.42246500 0.89452800<br>H 1.81002400 3.42238200 -0.89463300<br>H 3.34909800 3.51680500 -0.00000200<br>H -0.81440700 -3.50652400 -0.89476900<br>H -0.81434500 -3.50647300 0.89486900<br>H 0.28659900 -4.58723200 0.00004200<br>H -5.68598800 0.42217100 0.00008700<br>H -4.86129900 2.08469400 0.00002200	• Electronic Energy<br>= -652.509606<br>• Zero Point Energy Coorection<br>= 0.218858<br>• Thermal Correction to Energy<br>= 0.233159<br>• Thermal Correction to Enthalpy<br>= 0.234103<br>• Thermal Correction to Free Energy<br>= 0.177222

Name	HAs 1'-CH radical anion
Cartesian Coordinate:	Thermochemical Values:
O 2.63188000 -1.55452900 -0.00023300<br>O 0.72163800 2.73325000 0.00053000<br>O 2.84946100 1.11097600 0.00015600<br>C -0.96166800 -0.58179700 -0.00010200<br>C -2.23355600 -1.20770000 -0.00023800<br>C -0.78485900 0.82929500 0.00015400<br>C 0.20674900 -1.39234400 -0.00021500<br>C 1.46379800 -0.82924700 -0.00012400<br>C 0.47777400 1.38061300 0.00025500<br>C 1.67395000 0.58794500 0.00009500<br>C -3.49417100 -0.55987400 -0.00025100<br>C 2.51600800 -2.96956500 -0.00044500<br>C -0.40591500 3.59635000 0.00071100<br>C -4.69205700 -1.20490700 -0.00033900<br>H -2.23954200 -2.29564000 -0.00035600<br>H -1.65339800 1.47344800 0.00028800<br>H 0.08181200 -2.46836600 -0.00039000<br>H -3.50928500 0.52775600 -0.00019500<br>H 1.99200800 -3.32059300 -0.89372200<br>H 1.99201100 -3.32086100 0.89272800<br>H 3.53327600 -3.35611700 -0.00050500<br>H -1.01770500 3.44280100 0.89392200<br>H -1.01767500 3.44321800 -0.89259200<br>H -0.01025500 4.61008100 0.00095600<br>H -5.62596700 -0.65649400 -0.00035600<br>H -4.74541900 -2.28942100 -0.00039800	• Electronic Energy<br>= -652.041649<br>• Zero Point Energy Coorection<br>= 0.206686<br>• Thermal Correction to Energy<br>= 0.220355<br>• Thermal Correction to Enthalpy<br>= 0.221299<br>• Thermal Correction to Free Energy<br>= 0.165761

Name	HAs HT 1-OH···•OOH Transition State
Cartesian Coordinate:	Thermochemical Values:
O -0.79000400 -2.18364100 -0.71695500<br>O -1.60013200 2.39203800 -0.43087600<br>C 1.46514300 0.51429800 0.35312700<br>C 2.87990000 0.72332400 0.84611000<br>C 0.60870500 1.60794800 0.19918700<br>C 1.04039800 -0.78240000 0.04952900<br>C -0.25058800 -0.98494000 -0.41597100<br>C -0.68979600 1.41302800 -0.25561700<br>C -1.13781700 0.11096400 -0.58503500<br>C 3.89013300 0.33958500 -0.20158600<br>C 0.00385900 -3.34448800 -0.46644300<br>C -1.20202200 3.72606800 -0.11414600<br>C 4.78914100 -0.62795700 -0.05142800<br>H 3.00829800 1.77448700 1.11651000<br>H 3.04454800 0.11997000 1.74361000<br>H 0.96585300 2.59903200 0.44491300<br>H 1.72470600 -1.61101900 0.18211700<br>H 3.84746600 0.90171300 -1.13245400<br>H 0.27552100 -3.39830100 0.59020100<br>H 0.90266100 -3.33489800 -1.08599000<br>H -0.62101200 -4.19276900 -0.73367100<br>H -0.36549000 4.03618000 -0.74368500	• Electronic Energy<br>= -804.035965<br>• Zero Point Energy Coorection<br>= 0.244561<br>• Thermal Correction to Energy<br>= 0.261750<br>• Thermal Correction to Enthalpy<br>= 0.262694<br>• Thermal Correction to Free Energy<br>= 0.197994

H -0.92843300 3.80465400 0.94010100<br>H -2.06946700 4.34836800 -0.31886500<br>H 5.49552700 -0.87036500 -0.83785600<br>H 4.84650400 -1.20472600 0.86738100<br>O -2.36291000 -0.07693200 -1.07184800<br>H -2.92881600 -0.64022400 -0.37780700<br>O -3.38913900 -1.19812200 0.80282700<br>O -2.33314100 -1.01973100 1.62687000<br>H -1.77759700 -1.81358300 1.51898900	

Name	HAs HT 1'-CH···•OOH Transition State
Cartesian Coordinate:	Thermochemical Values:
O 1.41535200 2.70319400 0.19673000<br>O 2.59054000 -1.74492400 -0.41412100<br>O 3.23036300 0.79805100 0.04249800<br>C -0.77437300 -0.20648600 -0.44466400<br>C -2.19012400 -0.62992800 -0.53968800<br>C 0.20883400 -1.20521900 -0.52560600<br>C -0.40002200 1.11953500 -0.19379500<br>C 0.94410400 1.44716700 -0.03794700<br>C 1.54540000 -0.87254300 -0.35963000<br>C 1.92117100 0.45269300 -0.11682600<br>C -3.26226100 0.36983300 -0.72007800<br>C 0.45543100 3.75239500 0.29164200<br>C 2.28303300 -3.12233100 -0.61799600<br>C -4.33892400 0.15187500 -1.47803200<br>H -2.32095100 -1.50926900 -1.17368600<br>H -0.08829200 -2.22983000 -0.71166300<br>H -1.15006100 1.89422200 -0.12528800<br>H -3.18095400 1.30393500 -0.17086500<br>H 3.76948300 -0.00321900 -0.02633000<br>H -0.23459400 3.57240700 1.11920200<br>H -0.10129000 3.85380300 -0.64279400<br>H 1.02514300 4.65938700 0.47968700<br>H 1.78657200 -3.26664000 -1.58002000<br>H 1.65012000 -3.49676900 0.18983900<br>H 3.23649500 -3.64474000 -0.61286900<br>H -5.12450000 0.89357900 -1.56714700<br>H -4.45886800 -0.77436100 -2.03232100<br>H -2.37649900 -1.11001100 0.57659400<br>O -2.29437200 -1.60007800 1.85186900<br>O -1.23390300 -0.93557700 2.39707300<br>H -0.44925300 -1.44978000 2.14098700	• Electronic Energy = -804.028047<br>• Zero Point Energy Coorection = 0.243903<br>• Thermal Correction to Energy = 0.261473<br>• Thermal Correction to Enthalpy = 0.262417<br>• Thermal Correction to Free Energy = 0.196961

Name	Anionic HAs HT 1'-CH···•OOH Transition State
Cartesian Coordinate:	Thermochemical Values:
O 1.42007900 2.72409600 0.20556000<br>O 2.61956100 -1.76249600 -0.38708700<br>O 3.22737900 0.77985100 0.10133700<br>C -0.73025100 -0.19529500 -0.49000600<br>C -2.11803400 -0.63122800 -0.58500000<br>C 0.27647000 -1.19697100 -0.55516600<br>C -0.35752500 1.14616400 -0.22851400<br>C 0.96288400 1.47545500 -0.03590600<br>C 1.59533300 -0.88126800 -0.35415500<br>C 2.01928100 0.48102500 -0.08061100<br>C -3.20965400 0.35730200 -0.70225500<br>C 0.45966900 3.77504200 0.27265100<br>C 2.29862100 -3.12953600 -0.63115900<br>C -4.31760300 0.17741000 -1.42607200<br>H -2.23865400 -1.48297300 -1.25984300<br>H -0.02964000 -2.21682100 -0.75264400<br>H -1.11816400 1.91281200 -0.18845600<br>H -3.12745000 1.26120200 -0.10187000<br>H -0.25255000 3.59416300 1.08089000<br>H -0.07119800 3.87220300 -0.67710100<br>H 1.02442300 4.68198100 0.47445900<br>H 1.82915100 -3.24562500 -1.61072100<br>H 1.63472800 -3.51274400 0.14736400<br>H 3.24375600 -3.66647500 -0.60857100<br>H -5.11683600 0.90995700 -1.42624900<br>H -4.45550500 -0.71194400 -2.03480400<br>H -2.25237500 -1.19518200 0.53538200<br>O -2.20874600 -1.79031800 1.80386200<br>O -1.29615700 -0.87427400 2.43699700<br>H -0.44177300 -1.08402300 2.03397300	• Electronic Energy = -803.572532<br>• Zero Point Energy Coorection = 0.231430<br>• Thermal Correction to Energy = 0.248660<br>• Thermal Correction to Enthalpy = 0.249604<br>• Thermal Correction to Free Energy = 0.184873

Name	HAs RAF 2'-C···•OOH Transition State

Cartesian Coordinate:	Thermochemical Values:
O -2.14735000 2.36014300 0.31102700<br>O -2.21292000 -2.27771300 0.05900600<br>O -3.37612500 0.04565800 0.65865500<br>C 0.49602800 0.05773500 -0.86468200<br>C 1.90443000 0.08789000 -1.40897100<br>C -0.16293200 -1.15493800 -0.67716100<br>C -0.14068500 1.25753000 -0.54397400<br>C -1.43675100 1.24726700 -0.03148000<br>C -1.46039900 -1.16070400 -0.16772800<br>C -2.09969300 0.03424200 0.15803400<br>C -1.50992500 3.62019700 0.12255000<br>C -1.61885700 -3.53920300 -0.23625700<br>C 4.27357000 0.42325900 -0.64454800<br>H 2.19801900 -0.90259100 -1.76576400<br>H 1.93882000 0.76131000 -2.27240000<br>H 0.33676800 -2.08273300 -0.92432000<br>H 0.37845100 2.19480800 -0.70119000<br>H -3.68021000 -0.86998200 0.73291900<br>H -1.26585200 3.77773700 -0.93071800<br>H -0.60391100 3.69133900 0.72889500<br>H -2.22850200 4.36874800 0.44827000<br>H -0.72547100 -3.69594800 0.37244500<br>H -1.36494200 -3.60675600 -1.29643100<br>H -2.36850900 -4.28712100 0.01085600<br>H 4.63164100 -0.22579300 -1.43668200<br>H 5.00634200 0.88774700 0.00362100<br>C 2.93201700 0.57645100 -0.41834300<br>O 2.70787300 -0.88972900 0.91060200<br>H 2.60507000 1.33163700 0.29168200<br>O 3.21804800 -0.50759800 2.12662400<br>H 2.56525300 0.11315200 2.48686200	• Electronic Energy<br>= -804.027436<br>• Zero Point Energy Coorection<br>= 0.247743<br>• Thermal Correction to Energy<br>= 0.265220<br>• Thermal Correction to Enthalpy<br>= 0.266165<br>• Thermal Correction to Free Energy<br>= 0.200097

Name	Anionic HAs RAF 2'-C···•OOH Transition State
Cartesian Coordinate:	Thermochemical Values:
O 2.27860200 -2.29426000 0.33417700<br>O 2.12005300 2.39019900 0.05482800<br>O 3.40113200 0.11714300 0.70110500<br>C -0.45735200 -0.10450600 -0.86668300<br>C -1.86852000 -0.19791500 -1.39579700<br>C 0.15869600 1.13143400 -0.67451400<br>C 0.24246100 -1.26704700 -0.53492400<br>C 1.53072500 -1.18834600 -0.01366100<br>C 1.45086300 1.20158700 -0.15641000<br>C 2.19500000 0.04883900 0.20528900<br>C 1.68267800 -3.56725500 0.14019600<br>C 1.44075400 3.58776200 -0.28716300<br>C -4.22417600 -0.63443200 -0.61526800<br>H -2.19755700 0.76954600 -1.78599000<br>H -1.90052500 -0.90190400 -2.23536200<br>H -0.38317000 2.03420800 -0.92924900<br>H -0.23163400 -2.22965600 -0.68878600<br>H 1.44693200 -3.73630300 -0.91432000<br>H 0.77311000 -3.67295100 0.73828000<br>H 2.41826300 -4.29938400 0.46835000<br>H 0.52533800 3.70462400 0.29983000<br>H 1.19567400 3.61242600 -1.35274500<br>H 2.12548800 4.40144800 -0.05493700<br>H -4.62268400 -0.07118100 -1.45268800<br>H -4.92680500 -1.09856300 0.06594900<br>C -2.87587000 -0.67786000 -0.37878600<br>O -2.79233300 0.89271300 0.84892000<br>H -2.50471800 -1.35145300 0.38941500<br>O -3.13283900 0.50026700 2.12102400<br>H -2.36618400 -0.00291900 2.43814200	• Electronic Energy<br>= -803.553266<br>• Zero Point Energy Coorection<br>= 0.235445<br>• Thermal Correction to Energy<br>= 0.252278<br>• Thermal Correction to Enthalpy<br>= 0.253222<br>• Thermal Correction to Free Energy<br>= 0.189025

Name	HAs RAF 3'-C···•OOH Transition State
Cartesian Coordinate:	Thermochemical Values:
O -0.45136200 2.74390900 -0.24575300<br>O -3.17436700 -0.95902100 0.42113200<br>O -2.74924700 1.66946500 0.50422200<br>C 0.28607900 -0.82594000 -0.85615300<br>C 1.43627100 -1.72484400 -1.27240900<br>C -0.92505200 -1.38124700 -0.44956800<br>C 0.47893500 0.55591900 -0.80623200<br>C -0.54162400 1.38806300 -0.34400200<br>C -1.94426800 -0.54490100 0.00226600<br>C -1.75794400 0.83635800 0.05903100<br>C 2.26834500 -2.03856000 -0.06921800<br>C 0.80976400 3.33130600 -0.55696700	• Electronic Energy<br>= -804.033087<br>• Zero Point Energy Coorection<br>= 0.248993<br>• Thermal Correction to Energy<br>= 0.265961<br>• Thermal Correction to Enthalpy

C -3.43584400 -2.36045100 0.40317400<br>H 1.03382800 -2.65027200 -1.69509600<br>H 2.04741900 -1.23196500 -2.03133700<br>H -1.06636500 -2.45401300 -0.48758700<br>H 1.42508900 0.97163300 -1.12950800<br>H 1.76826900 -2.56303200 0.74084200<br>H -3.51227400 1.12821900 0.75192800<br>H 1.06597800 3.16905400 -1.60645600<br>H 1.59425000 2.92251000 0.08550100<br>H 0.69651600 4.39630600 -0.36824400<br>H -2.74019200 -2.88933600 1.05839600<br>H -3.36308400 -2.75184800 -0.61384600<br>H -4.45196400 -2.47882400 0.77164000<br>C 3.54274600 -1.57409700 0.12000800<br>O 3.29969400 0.29191500 0.74966900<br>H 4.10603300 -1.19133400 -0.72470100<br>H 4.11124600 -1.90538400 0.98024200<br>O 2.38141800 0.38177700 1.76728300<br>H 1.53172800 0.53430100 1.31622200	= 0.266906<br>• Thermal Correction to Free Energy<br>= 0.202999

Name	HAs HT 1-OH···•OOH Reactant Complex
Cartesian Coordinate:	Thermochemical Values:
O -1.52244400 -1.48668700 -0.28755600<br>O -0.30642700 3.02055300 -0.21291500<br>C 1.76623600 0.03168300 0.43307700<br>C 3.18568700 -0.37606100 0.77364400<br>C 1.42768200 1.37582800 0.28882700<br>C 0.79737500 -0.95212800 0.24452500<br>C -0.50432200 -0.58716900 -0.08336100<br>C 0.12206300 1.73722000 -0.04115900<br>C -0.85478300 0.75581100 -0.23177800<br>C 3.83465700 -1.11783000 -0.36255000<br>C -1.15834600 -2.85552300 -0.50488500<br>C 0.65554700 4.05694600 -0.04111900<br>C 4.25670100 -2.37670200 -0.29078900<br>H 3.76378000 0.52341900 1.00305700<br>H 3.18669100 -1.01046000 1.66454100<br>H 2.18636000 2.13395000 0.43378100<br>H 1.06654100 -1.99627400 0.34988800<br>H 3.93425500 -0.56630100 -1.29585200<br>H -0.74761600 -3.29427800 0.40555300<br>H -0.43645700 -2.92430800 -1.32014100<br>H -2.08012100 -3.36535300 -0.77705200<br>H 1.46946400 3.95493900 -0.76268800<br>H 1.05626900 4.05080100 0.97518100<br>H 0.12321500 4.98852200 -0.21907200<br>H 4.71037600 -2.87178000 -1.14251600<br>H 4.16311200 -2.94896000 0.62802900<br>O -2.11155200 1.16528300 -0.57885300<br>H -2.73868400 0.42290000 -0.56923400<br>O -4.35005300 -0.49311200 0.05961100<br>O -3.94112600 -1.42014400 0.87514500<br>H -2.97535300 -1.53640700 0.67647600	• Electronic Energy<br>= -804.056285<br>• Zero Point Energy Coorection<br>= 0.250026<br>• Thermal Correction to Energy<br>= 0.267879<br>• Thermal Correction to Enthalpy<br>= 0.268823<br>• Thermal Correction to Free Energy<br>= 0.201974

Name	HAs HT 1'-CH···•OOH Reactant Complex
Cartesian Coordinate:	Thermochemical Values:
O 0.38758700 2.87223500 0.05165700<br>O 2.79060700 -1.08553700 -0.28967600<br>O 2.64753500 1.52432200 0.24199500<br>C -0.79349800 -0.48833300 -0.94799600<br>C -2.07095400 -1.20084800 -1.34487700<br>C 0.40766400 -1.18656400 -0.84546100<br>C -0.83927300 0.87722400 -0.64702400<br>C 0.32094900 1.54851400 -0.25889400<br>C 1.56208000 -0.51409200 -0.44421500<br>C 1.52553600 0.84926200 -0.15573200<br>C -3.00154200 -1.34202400 -0.17067500<br>C -0.83279800 3.60824600 0.02542900<br>C 2.88704300 -2.49485600 -0.48463200<br>C -4.20003800 -0.77142700 -0.09239300<br>H -2.57384400 -0.64516200 -2.14091100<br>H 0.43465000 -2.24499400 -1.07040900<br>H -1.78213200 1.40493900 -0.72459600<br>H -2.62862500 -1.93767900 0.66140900<br>H 3.38172500 0.89557300 0.28771600<br>H -1.55210800 3.18738800 0.73210500<br>H -1.25928200 3.61701900 -0.98016100<br>H -0.57455300 4.62180600 0.32308700<br>H 2.63392300 -2.75947100 -1.51355400<br>H 2.23017800 -3.02295600 0.21062700<br>H 3.92365600 -2.75346000 -0.28268800	• Electronic Energy<br>= -804.054469<br>• Zero Point Energy Coorection<br>= 0.248937<br>• Thermal Correction to Energy<br>= 0.267390<br>• Thermal Correction to Enthalpy<br>= 0.268334<br>• Thermal Correction to Free Energy<br>= 0.199975

H -4.82848500 -0.89111700 0.78336700<br>H -4.58968000 -0.16899400 -0.90833600<br>H -1.81519300 -2.19042300 -1.73299900<br>O -0.35539400 -1.69646700 2.32092500<br>O -0.46611800 -0.39997000 2.31486300<br>H -0.82498300 -0.13974900 1.43604800	

Name	Anionic HAs HT 1'-CH···•OOH Reactant Complex
Cartesian Coordinate:	Thermochemical Values:
O 0.31001500 2.95726700 0.34799000<br>O 2.94323600 -0.84659800 -0.34659900<br>O 2.64251300 1.69212700 0.36626100<br>C -0.66701600 -0.38342900 -0.82849400<br>C -1.89552500 -1.14568600 -1.26163000<br>C 0.58138700 -1.02695300 -0.81652200<br>C -0.79907100 0.96328200 -0.44151000<br>C 0.30876100 1.67526700 -0.03962700<br>C 1.70749900 -0.33864700 -0.41270100<br>C 1.63144800 1.05876000 -0.00045700<br>C -3.03450000 -1.00184800 -0.28848500<br>C -0.94054900 3.64843200 0.35400600<br>C 3.11655400 -2.21891400 -0.70698000<br>C -4.21657600 -0.48023800 -0.60017900<br>H -2.21204400 -0.77761400 -2.24414300<br>H 0.63835400 -2.06576000 -1.11363000<br>H -1.78042500 1.41979400 -0.46111500<br>H -2.84145100 -1.35505700 0.72252100<br>H -1.63949000 3.16169500 1.03705800<br>H -1.36038700 3.68242700 -0.65332300<br>H -0.72039300 4.65481500 0.70007800<br>H 2.83022200 -2.37659900 -1.74861300<br>H 2.52391100 -2.85981700 -0.05100900<br>H 4.17511600 -2.42678100 -0.57575800<br>H -5.01131400 -0.40309200 0.13377700<br>H -4.42521300 -0.11773600 -1.60285000<br>H -1.63133600 -2.20074900 -1.37150300<br>O -0.72552200 -2.69691400 1.78323700<br>O -0.57939700 -1.34774400 2.32556700<br>H -0.02677900 -0.90080200 1.67233800	• Electronic Energy = -803.587364<br>• Zero Point Energy Coorection = 0.236041<br>• Thermal Correction to Energy = 0.254091<br>• Thermal Correction to Enthalpy = 0.255035<br>• Thermal Correction to Free Energy = 0.188422

Name	HAs RAF 2'-C···•OOH Reactant Complex
Cartesian Coordinate:	Thermochemical Values:
O -2.80967100 1.27411800 0.26433900<br>O -0.52659300 -2.72058000 -0.37215200<br>O -2.69472400 -1.35951400 0.38389600<br>C 0.61322200 0.73223700 -1.02218800<br>C 1.85536000 1.50061500 -1.42411700<br>C 0.65726900 -0.66088600 -0.96314000<br>C -0.53482800 1.41654900 -0.61659300<br>C -1.64329400 0.70707900 -0.15244600<br>C -0.45008500 -1.36419100 -0.49338600<br>C -1.60000500 -0.68635500 -0.08793500<br>C -2.89138700 2.69641000 0.22344900<br>C 0.64332400 -3.47318700 -0.68655400<br>C 3.77673000 1.26515300 0.17024700<br>H 2.47007900 0.88761700 -2.08648900<br>H 1.56774800 2.40909400 -1.95946400<br>H 1.55738700 -1.18212200 -1.26432500<br>H -0.55338800 2.49796300 -0.66360100<br>H -2.48949700 -2.30489000 0.39737800<br>H -2.77361500 3.06206000 -0.79916800<br>H -2.13450100 3.14660600 0.86992300<br>H -3.88377800 2.94741300 0.59061200<br>H 1.47642100 -3.17138500 -0.04750300<br>H 0.91205500 -3.34573900 -1.73742900<br>H 0.38894000 -4.51296900 -0.49562600<br>H 4.23278400 0.49986100 -0.45198700<br>H 4.28445800 1.52606000 1.09257500<br>C 2.64216800 1.85806100 -0.18939000<br>O 1.75101200 -0.95436300 2.07074100<br>H 2.19896800 2.61566600 0.45560200<br>O 0.95370500 0.06703500 2.18322500<br>H 0.94524700 0.52230100 1.31142600	• Electronic Energy = -804.054783<br>• Zero Point Energy Coorection = 0.248823<br>• Thermal Correction to Energy = 0.267378<br>• Thermal Correction to Enthalpy = 0.268322<br>• Thermal Correction to Free Energy = 0.199667

Name	Anionic HAs RAF 2'-C···•OOH Reactant Complex

Cartesian Coordinate:	Thermochemical Values:
O 2.97719300 -1.04990200 0.17022000<br>O 0.31721700 2.78127800 -0.34150400<br>O 2.65486400 1.61051300 0.26788000<br>C -0.54879000 -0.77199400 -0.93041800<br>C -1.73859000 -1.62730600 -1.31905800<br>C -0.68572200 0.61855400 -0.85285800<br>C 0.67706500 -1.34974200 -0.58824900<br>C 1.74508800 -0.54897400 -0.18527900<br>C 0.38583400 1.41124900 -0.45063500<br>C 1.65476000 0.86752600 -0.10457600<br>C 3.14305300 -2.45918200 0.13004600<br>C -0.93560500 3.39131200 -0.61490600<br>C -3.92122400 -1.29628600 -0.12346300<br>H -2.26978100 -1.17104600 -2.15898700<br>H -1.38004400 -2.60920000 -1.64159600<br>H -1.64287600 1.06369900 -1.09925000<br>H 0.78209500 -2.42675200 -0.63932000<br>H 2.99727900 -2.84480200 -0.88273700<br>H 2.44984800 -2.95655300 0.81426100<br>H 4.16618900 -2.65206600 0.44705900<br>H -1.70952600 3.01773000 0.06172900<br>H -1.24082800 3.21883500 -1.65069100<br>H -0.79426400 4.45806800 -0.45201200<br>H -4.33397700 -0.75010300 -0.96735700<br>H -4.55519600 -1.41979800 0.74801500<br>C -2.68801700 -1.79297200 -0.16300800<br>O -1.73482800 0.56152800 2.29561200<br>H -2.29471000 -2.32951200 0.69967800<br>O -0.73093000 -0.26012400 2.19573500<br>H -0.56944700 -0.39794400 1.23209200	• Electronic Energy<br>= -803.582623<br>• Zero Point Energy Coorection<br>= 0.236341<br>• Thermal Correction to Energy<br>= 0.254252<br>• Thermal Correction to Enthalpy<br>= 0.255197<br>• Thermal Correction to Free Energy<br>= 0.187545

Name	HAs RAF 3’-C···•OOH Reactant Complex
Cartesian Coordinate:	Thermochemical Values:
O -0.32176300 2.73444200 -0.40382500<br>O -2.89544900 -1.07000600 0.26855300<br>O -2.57549600 1.57177800 0.33811200<br>C 0.56307700 -0.80478900 -1.00122500<br>C 1.75550100 -1.65513900 -1.38880800<br>C -0.62916500 -1.40758200 -0.59552300<br>C 0.69840200 0.58385500 -0.95784600<br>C -0.35722700 1.37650600 -0.50566600<br>C -1.68251000 -0.61043700 -0.15151000<br>C -1.55139400 0.77818000 -0.10453400<br>C 2.54329800 -2.00624500 -0.15309400<br>C 0.89979600 3.38024800 -0.75401700<br>C -3.10458000 -2.48021300 0.24905100<br>H 1.40917600 -2.56679400 -1.88227100<br>H 2.39133900 -1.10434300 -2.08511000<br>H -0.72517800 -2.48563000 -0.62639100<br>H 1.63584500 1.03422400 -1.25931200<br>H 2.06777400 -2.70696300 0.53188400<br>H -3.31549800 0.99987300 0.58726100<br>H 1.14221800 3.20797600 -1.80521700<br>H 1.71799700 3.02984300 -0.12021900<br>H 0.73312100 4.44168300 -0.58581800<br>H -2.39316600 -2.98300800 0.90786200<br>H -3.01183400 -2.86807400 -0.76771700<br>H -4.11748300 -2.63632400 0.61228200<br>C 3.71848100 -1.46898200 0.16064900<br>O 1.79661900 0.98114600 2.07453900<br>H 4.20727400 -0.76042400 -0.50254100<br>H 4.22800600 -1.72122100 1.08437600<br>O 0.99124800 -0.03264000 2.19846200<br>H 0.94706100 -0.47099200 1.31933400	• Electronic Energy<br>= -804.054742<br>• Zero Point Energy Coorection<br>= 0.248593<br>• Thermal Correction to Energy<br>= 0.267233<br>• Thermal Correction to Enthalpy<br>= 0.268177<br>• Thermal Correction to Free Energy<br>= 0.199116

Name	HAs HT 1-OH···•OOH Product Complex
Cartesian Coordinate:	Thermochemical Values:
O -1.43704800 -1.57839800 -0.21291600<br>O -0.37347200 2.97783100 -0.18523500<br>C 1.78468300 0.07707700 0.40985500<br>C 3.21630600 -0.28701900 0.71408600<br>C 1.41349200 1.41966700 0.27096400<br>C 0.84887900 -0.96541000 0.24948300<br>C -0.46150200 -0.67403600 -0.03855500<br>C 0.10239100 1.74108800 -0.02417200<br>C -0.90828700 0.70460900 -0.19318300<br>C 3.84583800 -1.01203600 -0.44734900<br>C -1.07732400 -2.96300100 -0.15652600<br>C 0.54033600 4.06907700 -0.04386100	• Electronic Energy<br>= -804.072713<br>• Zero Point Energy Coorection<br>= 0.249199<br>• Thermal Correction to Energy<br>= 0.267509<br>• Thermal Correction to Enthalpy

C 4.26998400 -2.27041800 -0.39509900<br>H 3.77830600 0.62322600 0.93555700<br>H 3.24228100 -0.93215000 1.59730500<br>H 2.16289600 2.18964800 0.39739000<br>H 1.18566700 -1.98895400 0.35764000<br>H 3.92957500 -0.44617000 -1.37278900<br>H -0.69119300 -3.21449300 0.83304500<br>H -0.33457900 -3.18965800 -0.92328700<br>H -1.99565400 -3.51181900 -0.35053200<br>H 1.33790200 3.99263700 -0.78517200<br>H 0.95981000 4.08288500 0.96370200<br>H -0.04587700 4.96742400 -0.21693000<br>H 4.71313400 -2.75308100 -1.25927800<br>H 4.19081300 -2.85332200 0.51814400<br>O -2.09544600 0.99774900 -0.46140500<br>H -3.50651100 -0.04431700 -0.60994100<br>O -4.23823400 -0.67708300 -0.44004500<br>O -4.00728700 -1.02681600 0.92048900<br>H -3.16344300 -1.50572000 0.87419200	= 0.268453<br>• Thermal Correction to Free Energy<br>= 0.199756

Name	HAs HT 1'-CH⋯•OOH Product Complex
Cartesian Coordinate:	Thermochemical Values:
O 1.25903500 2.73961200 0.14238500<br>O 2.50379800 -1.68470900 -0.50846600<br>O 3.09848900 0.85725200 0.01485000<br>C -0.88694200 -0.18312100 -0.62585600<br>C -2.24903600 -0.59619800 -0.80741300<br>C 0.12184100 -1.17019800 -0.69116600<br>C -0.52342800 1.15244000 -0.34711600<br>C 0.80614700 1.48575100 -0.13570800<br>C 1.44696200 -0.82453700 -0.47688100<br>C 1.80067900 0.50137400 -0.19898000<br>C -3.39338400 0.22398800 -0.71485200<br>C 0.28826300 3.77951500 0.22307600<br>C 2.21765500 -3.06327800 -0.73436700<br>C -4.66035800 -0.24189000 -0.89659500<br>H -2.40815400 -1.64772000 -1.03409500<br>H -0.15592700 -2.19560000 -0.89825300<br>H -1.28226400 1.91947500 -0.28852900<br>H -3.26463000 1.27825700 -0.48583700<br>H 3.64749000 0.06174600 -0.04383900<br>H -0.42943600 3.57813900 1.02172000<br>H -0.23547500 3.89537000 -0.72862200<br>H 0.84348900 4.68680200 0.44926800<br>H 1.75512700 -3.20426000 -1.71364000<br>H 1.56215900 -3.45104000 0.04889000<br>H 3.17592300 -3.57588000 -0.70195900<br>H -5.51752400 0.41464600 -0.81600700<br>H -4.84351400 -1.28651200 -1.12678300<br>H -2.04143200 -1.33730800 1.58997900<br>O -1.43714600 -1.76656600 2.21730100<br>O -0.52235200 -0.73194300 2.54548600<br>H 0.16917700 -0.82837300 1.87040100	• Electronic Energy<br>= -804.069852<br>• Zero Point Energy Coorection<br>= 0.248295<br>• Thermal Correction to Energy<br>= 0.266837<br>• Thermal Correction to Enthalpy<br>= 0.267782<br>• Thermal Correction to Free Energy<br>= 0.200864

Name	Anonic HAs HT 1'-CH⋯•OOH Product Complex
Cartesian Coordinate:	Thermochemical Values:
O 1.19980000 2.78112800 0.09839600<br>O 2.56459400 -1.66897900 -0.49304900<br>O 3.10245100 0.90699900 -0.01825000<br>C -0.86517700 -0.22599900 -0.61230800<br>C -2.19986500 -0.67690100 -0.76631100<br>C 0.19171500 -1.17898600 -0.66281100<br>C -0.51853400 1.13026400 -0.35672900<br>C 0.79402700 1.49584500 -0.15793000<br>C 1.50194100 -0.80195200 -0.46152300<br>C 1.88150100 0.55769700 -0.20103100<br>C -3.37672500 0.11578500 -0.69494600<br>C 0.19169900 3.77871500 0.17153400<br>C 2.27257900 -3.04368400 -0.69891500<br>C -4.63608800 -0.36641100 -0.86019600<br>H -2.33388200 -1.73809100 -0.96861100<br>H -0.06362200 -2.21456100 -0.85181100<br>H -1.29857200 1.87745000 -0.30632500<br>H -3.26599000 1.17863700 -0.49243400<br>H -0.51575300 3.56240600 0.97670300<br>H -0.34662900 3.86298600 -0.77640300<br>H 0.70710500 4.71377300 0.38148600<br>H 1.80049200 -3.20364400 -1.67200900<br>H 1.62162200 -3.42698400 0.09194300	• Electronic Energy<br>= -803.603577<br>• Zero Point Energy Coorection<br>= 0.235484<br>• Thermal Correction to Energy<br>= 0.253638<br>• Thermal Correction to Enthalpy<br>= 0.254582<br>• Thermal Correction to Free Energy<br>= 0.188201

H 3.22823100 -3.56310300 -0.66858000<br>H -5.50029900 0.28271300 -0.79162900<br>H -4.81157400 -1.41826300 -1.06480700<br>H -1.85395200 -1.31236000 1.48171900<br>O -1.34178800 -1.68236200 2.22264900<br>O -0.40922200 -0.65266000 2.51779300<br>H 0.28370400 -0.78021300 1.84743900	

Name	HAs RAF 1-C···•OOH Product
Cartesian Coordinate:	Thermochemical Values:
O 0.25508600 2.56921900 -0.36821000<br>O 2.26731800 -1.66522200 -0.39188600<br>O 1.88683000 0.78148000 -1.58506500<br>C -1.21770100 -0.73756800 0.40504300<br>C -2.54809300 -1.33967700 0.77824500<br>C -0.08138100 -1.55294400 0.20807600<br>C -1.12786600 0.66433600 0.20200700<br>C 0.04641000 1.24978200 -0.16114900<br>C 1.11418900 -1.00247400 -0.15296400<br>C -3.52351200 -1.25403700 -0.36726000<br>C -0.86681200 3.43508100 -0.21286100<br>C 2.23701700 -3.08818700 -0.29707700<br>C -4.65063600 -0.54927300 -0.33831100<br>H -2.40023000 -2.38616300 1.05814900<br>H -2.96672400 -0.81391500 1.64235300<br>H -0.17408400 -2.62375000 0.34302400<br>H -2.02033100 1.26446400 0.33760200<br>H -3.24640400 -1.79789300 -1.26844300<br>H 2.58810200 0.13691300 -1.76071700<br>H -1.24985600 3.38326700 0.80900500<br>H -1.65539900 3.16862000 -0.92035900<br>H -0.50302300 4.43785300 -0.42246000<br>H 1.52071500 -3.50090200 -1.01093100<br>H 1.97364900 -3.39498700 0.71745800<br>H 3.24152200 -3.42619500 -0.53851100<br>H -5.31491400 -0.50787600 -1.19470700<br>H -4.94354900 0.00569500 0.54866000<br>C 1.31536100 0.47204200 -0.34279200<br>O 2.34138300 0.95843600 0.58005000<br>O 1.93078100 0.69844400 1.90934600<br>H 1.51748900 1.53351800 2.18025400	• Electronic Energy = -804.050832<br>• Zero Point Energy Coorection = 0.250543<br>• Thermal Correction to Energy = 0.267677<br>• Thermal Correction to Enthalpy = 0.268621<br>• Thermal Correction to Free Energy = 0.204454

Name	Anionic HAs RAF 1-C···•OOH Product
Cartesian Coordinate:	Thermochemical Values:
O 0.18513400 2.59053500 -0.39747100<br>O 2.35949200 -1.61571600 -0.43555600<br>O 1.98648300 0.86456600 -1.56388100<br>C -1.15183600 -0.77832800 0.34374000<br>C -2.45585500 -1.43014500 0.73142300<br>C 0.01290200 -1.55189200 0.13503300<br>C -1.11089700 0.62918200 0.15428500<br>C 0.04648900 1.25372800 -0.20439400<br>C 1.19244900 -0.95895900 -0.22267900<br>C -3.47948100 -1.31699300 -0.36774600<br>C -0.97265500 3.39896200 -0.22659200<br>C 2.35273000 -3.03085600 -0.29033100<br>C -4.62558000 -0.65095800 -0.25920500<br>H -2.27345500 -2.48538700 0.95386100<br>H -2.85700900 -0.96188000 1.63691300<br>H -0.05007300 -2.62601500 0.26475900<br>H -2.02716000 1.19259800 0.29356100<br>H -3.22375500 -1.80464800 -1.30684700<br>H -1.35136200 3.32040300 0.79579200<br>H -1.75434800 3.10725900 -0.93258000<br>H -0.65996300 4.42156800 -0.42550200<br>H 1.65795600 -3.48778000 -0.99948700<br>H 2.07868800 -3.31367100 0.72917000<br>H 3.36681100 -3.36131500 -0.50357700<br>H -5.32483400 -0.58611500 -1.08585000<br>H -4.89897100 -0.15102200 0.66603700<br>C 1.36083100 0.53202500 -0.44296100<br>O 2.30918200 1.01923000 0.65492200<br>O 1.79616500 0.72696200 1.95098100<br>H 1.21124000 1.47677700 2.13835500	• Electronic Energy = -803.576093<br>• Zero Point Energy Coorection = 0.236801<br>• Thermal Correction to Energy = 0.253654<br>• Thermal Correction to Enthalpy = 0.254598<br>• Thermal Correction to Free Energy = 0.191040

Name	HAs RAF 2-C···•OOH Product
Cartesian Coordinate:	Thermochemical Values:

O 0.25999900 2.17651900 -1.10146900<br>O 2.80734100 -1.41518100 -0.16137100<br>O 2.59094900 1.23809900 -0.00612000<br>C -0.90707000 -0.99939800 -0.15058900<br>C -2.12441100 -1.90085300 -0.15459100<br>C 0.37421100 -1.65788200 -0.19852000<br>C -1.00203800 0.34579400 -0.07641500<br>C 1.53742400 -0.91055200 -0.14516100<br>C 1.48250900 0.47691000 -0.07016500<br>C -3.42834700 -1.19412700 -0.37055500<br>C -0.96486400 2.83991400 -1.43257400<br>C 2.94342600 -2.83318000 -0.21642200<br>C -4.44406900 -1.22151800 0.48632400<br>H -1.98148300 -2.64419500 -0.94752200<br>H -2.15308800 -2.45627000 0.78876800<br>H 0.40675900 -2.73754000 -0.26283800<br>H -1.96351200 0.83934900 0.00230800<br>H -3.52619400 -0.63947700 -1.30216100<br>H 3.37360100 0.66591700 -0.03500300<br>H -1.45540000 3.23366200 -0.54089700<br>H -1.63906500 2.16543200 -1.96381400<br>H -0.68731800 3.66401700 -2.08679600<br>H 2.49774600 -3.22731800 -1.13236900<br>H 2.47812100 -3.29797300 0.65570200<br>H 4.01268500 -3.03061700 -0.21294600<br>H -5.37631500 -0.70857500 0.27633500<br>H -4.37287500 -1.76214500 1.42601500<br>C 0.19582400 1.23582700 -0.05695700<br>O 0.16421200 2.11662600 1.11430300<br>O 0.10026800 1.33089800 2.29005300<br>H 1.02861400 1.27445400 2.56579600	• Electronic Energy = -804.040421<br>• Zero Point Energy Coorection = 0.249843<br>• Thermal Correction to Energy = 0.267135<br>• Thermal Correction to Enthalpy = 0.268079<br>• Thermal Correction to Free Energy = 0.204052

Name	Anionic HAs RAF 2-C⋯•OOH Product
Cartesian Coordinate:	Thermochemical Values:
O 0.15607900 2.16844400 -1.13831900<br>O 2.87019100 -1.35374600 -0.20504900<br>O 2.57345000 1.30480800 -0.05740000<br>C -0.83831100 -1.03782100 -0.06962500<br>C -2.03308900 -1.97046400 0.01272900<br>C 0.45537600 -1.64923100 -0.15712900<br>C -0.98415500 0.31097000 -0.02208200<br>C 1.58811400 -0.85674700 -0.15633000<br>C 1.53273000 0.56161200 -0.09728500<br>C -3.34065000 -1.34251700 -0.36698100<br>C -1.10879800 2.75617000 -1.44257000<br>C 3.01451600 -2.76549900 -0.25313300<br>C -4.39595500 -1.25811600 0.43725600<br>H -1.84019000 -2.82197000 -0.64955500<br>H -2.10168400 -2.37582800 1.02825700<br>H 0.51938700 -2.72808500 -0.20483100<br>H -1.96489900 0.76131300 0.08253500<br>H -3.40315100 -0.94074800 -1.37706100<br>H -1.58767800 3.16054700 -0.54836800<br>H -1.77320400 2.03263600 -1.92056900<br>H -0.90391600 3.56860700 -2.13806500<br>H 2.54525500 -3.17840100 -1.15015800<br>H 2.58207400 -3.23491700 0.63455600<br>H 4.08508200 -2.95908400 -0.28183800<br>H -5.32539500 -0.80596300 0.10834500<br>H -4.36081800 -1.64242800 1.45298200<br>C 0.17576600 1.23528900 -0.07159100<br>O 0.14110000 2.17328100 1.07595600<br>O 0.25000200 1.44243500 2.28341400<br>H 1.20929600 1.38066700 2.41539900	• Electronic Energy = -803.582007<br>• Zero Point Energy Coorection = 0.237131<br>• Thermal Correction to Energy = 0.254170<br>• Thermal Correction to Enthalpy = 0.255114<br>• Thermal Correction to Free Energy = 0.190727

Name	HAs RAF 3-C⋯•OOH Product
Cartesian Coordinate:	Thermochemical Values:
O 0.03583900 2.63742300 -0.34303900<br>O 3.06186000 -0.91051200 -0.19431800<br>O 2.53283400 1.66893800 -0.52314500<br>C -0.60374100 -1.05078900 0.14050900<br>C -1.76223900 -1.99791300 0.31653300<br>C 0.69054800 -1.48671200 0.07320200<br>C 0.21989700 1.28471400 -0.24436900<br>C 1.75072200 -0.58242000 -0.12233800<br>C 1.49103800 0.80818700 -0.29719300<br>C -2.64727700 -2.00993200 -0.90342400<br>C -1.18256900 3.07463400 -0.95281600<br>C 3.41522400 -2.28726400 -0.05913300<br>C -3.88212900 -1.51791200 -0.93429300<br>H -1.37038000 -3.00147200 0.50074900	• Electronic Energy = -804.039981<br>• Zero Point Energy Coorection = 0.250158<br>• Thermal Correction to Energy = 0.267630<br>• Thermal Correction to Enthalpy = 0.268574

H -2.35682400 -1.70104200 1.18695600<br>H 0.89653600 -2.54745000 0.15694300<br>H -2.20594600 -2.42590300 -1.80713000<br>H 3.34942600 1.15268400 -0.57384600<br>H -2.04181500 2.85824900 -0.31514400<br>H -1.31139700 2.60279100 -1.92975000<br>H -1.08445700 4.15100100 -1.07609900<br>H 2.96026400 -2.87690700 -0.85733900<br>H 3.10227800 -2.66643400 0.91562200<br>H 4.49840000 -2.32508000 -0.14165800<br>H -4.47518100 -1.52927000 -1.84242900<br>H -4.33821600 -1.09086400 -0.04510000<br>C -0.94021400 0.40699400 0.08104700<br>O -1.57397400 0.85592600 1.32617700<br>H -1.77920000 0.57508600 -0.60735000<br>O -0.67218200 0.67860600 2.40835900<br>H -0.21405900 1.53204500 2.45695200	• Thermal Correction to Free Energy = 0.203443

Name	Anionic HAs RAF 3-C···•OOH Product
Cartesian Coordinate:	Thermochemical Values:
O -0.02089500 2.67304800 -0.27304600<br>O 3.07617100 -0.89802100 -0.25287400<br>O 2.54675500 1.69506900 -0.54347200<br>C -0.57990800 -1.03387000 0.13533400<br>C -1.72459400 -1.99775700 0.31004800<br>C 0.72252500 -1.45149900 0.02927400<br>C 0.22624000 1.30315400 -0.22267500<br>C 1.77056300 -0.53743400 -0.16478600<br>C 1.53982300 0.89770100 -0.32040100<br>C -2.61894400 -2.00903300 -0.90298000<br>C -1.14491900 3.06587300 -1.05434400<br>C 3.40134600 -2.27563200 -0.09960700<br>C -3.86165700 -1.53554600 -0.92182100<br>H -1.32043600 -2.99954700 0.47942600<br>H -2.31887300 -1.72056700 1.18769000<br>H 0.93315000 -2.51365200 0.08477800<br>H -2.17714400 -2.40609500 -1.81514600<br>H -2.08601700 2.82660700 -0.55104900<br>H -1.12158700 2.58225000 -2.03552200<br>H -1.07586200 4.14577400 -1.17988600<br>H 2.94081100 -2.87387800 -0.88917500<br>H 3.08163800 -2.64259800 0.87859100<br>H 4.48438600 -2.33776600 -0.17863000<br>H -4.45913800 -1.54325400 -1.82714900<br>H -4.31966000 -1.12682100 -0.02497400<br>C -0.91293300 0.42084100 0.12471700<br>O -1.50713800 0.83255300 1.42334800<br>H -1.79458800 0.60657700 -0.50487600<br>O -0.56223100 0.64140600 2.46622800<br>H 0.06678100 1.36974100 2.33788200	• Electronic Energy = -803.566713<br>• Zero Point Energy Coorection = 0.237255<br>• Thermal Correction to Energy = 0.254354<br>• Thermal Correction to Enthalpy = 0.255299<br>• Thermal Correction to Free Energy = 0.190640

Name	HAs RAF 4-C···•OOH Product
Cartesian Coordinate:	Thermochemical Values:
O -1.17928000 2.56171800 -0.25929600<br>O 2.85475900 0.27770100 -0.59137100<br>O 1.39253600 2.47520000 -0.78189000<br>C -1.21029100 -2.24553400 -0.00177500<br>C 0.96960300 -1.01350300 0.21575500<br>C -1.21037400 0.23287800 0.39253400<br>C -0.58671000 1.35607800 -0.04564300<br>C 1.55220900 0.12859600 -0.22083500<br>C 0.80723600 1.33939300 -0.34931400<br>C -1.24950600 -2.16500900 -1.49911700<br>C -2.57547900 2.64424400 0.01157900<br>C 3.69110900 -0.87190600 -0.48768100<br>C -2.36786500 -2.03485900 -2.20575500<br>H -0.68628200 -3.15491700 0.31088900<br>H -2.22600500 -2.27225900 0.40131100<br>H 1.52368800 -1.93949600 0.30598100<br>H -2.27290400 0.21713200 0.60148400<br>H -0.29124300 -2.21435600 -2.01207800<br>H 2.32881500 2.29635300 -0.96040600<br>H -2.77750700 2.42199200 1.06218400<br>H -3.13278600 1.95500700 -0.62751700<br>H -2.86300100 3.66894900 -0.21131200<br>H 3.32450800 -1.67037500 -1.13681500<br>H 3.73293700 -1.22110700 0.54641600<br>H 4.67891100 -0.55436600 -0.81251000<br>H -2.34959800 -1.98286700 -3.28891200<br>H -3.33800000 -1.98037800 -1.71969900<br>C -0.46812000 -1.04542800 0.62365900	• Electronic Energy = -804.045383<br>• Zero Point Energy Coorection = 0.249870<br>• Thermal Correction to Energy = 0.267089<br>• Thermal Correction to Enthalpy = 0.268033<br>• Thermal Correction to Free Energy = 0.204111

O -0.55739100 -1.39153500 2.05494500<br>O 0.08947000 -0.40236600 2.83580900<br>H -0.56317100 0.31523900 2.87495400<br>H -0.21405900 1.53204500 2.45695200	

Name	Anionic HAs RAF 4-C···•OOH Product
Cartesian Coordinate:	Thermochemical Values:
O -0.91699600 2.67818700 -0.40834800<br>O 2.90344400 -0.03177300 -0.65574600<br>O 1.69779000 2.34768400 -0.84532000<br>C -1.40165500 -2.11529300 0.21507400<br>C 0.88358800 -1.05429700 0.17274500<br>C -1.14968100 0.38676800 0.30544400<br>C -0.40710400 1.43490300 -0.14732400<br>C 1.58682100 0.01771400 -0.27837000<br>C 1.00918600 1.33846300 -0.43564800<br>C -1.54924600 -2.20986600 -1.27400900<br>C -2.30054600 2.87820500 -0.15598400<br>C 3.56310000 -1.28438600 -0.54005100<br>C -2.71216600 -2.14011300 -1.91509300<br>H -0.93148600 -3.02779400 0.59846100<br>H -2.38506600 -2.01248300 0.68209800<br>H 1.33252400 -2.03600000 0.26131900<br>H -2.21684800 0.47047700 0.47154400<br>H -0.63046600 -2.34261700 -1.84184400<br>H -2.53485600 2.69759700 0.89653600<br>H -2.91137600 2.22492100 -0.78465000<br>H -2.50536200 3.91784700 -0.40335600<br>H 3.08915000 -2.03602800 -1.17688500<br>H 3.56377100 -1.63126100 0.49663200<br>H 4.58646400 -1.11893600 -0.87088700<br>H -2.76834600 -2.21921900 -2.99536500<br>H -3.64568400 -2.00382900 -1.37615600<br>C -0.52322200 -0.92206900 0.63718000<br>O -0.57180600 -1.10594000 2.13167900<br>O 0.17476200 -0.08592300 2.77273600<br>H -0.33442100 0.71926600 2.5787600	• Electronic Energy = -803.583568<br>• Zero Point Energy Coorection = 0.237033<br>• Thermal Correction to Energy = 0.253916<br>• Thermal Correction to Enthalpy = 0.254861<br>• Thermal Correction to Free Energy = 0.191708

Name	HAs RAF 2'-C···•OOH Product
Cartesian Coordinate:	Thermochemical Values:
O -2.15983500 2.38421100 0.27274500<br>O -2.29903200 -2.25492100 0.09512300<br>O -3.43590000 0.09785600 0.62422600<br>C 0.47071100 0.01900900 -0.80296600<br>C 1.89857600 0.01332100 -1.29262100<br>C -0.21210700 -1.17992700 -0.60841000<br>C -0.15219000 1.23482500 -0.51679800<br>C -1.45989200 1.25474300 -0.03541000<br>C -1.52126300 -1.15525300 -0.13104800<br>C -2.14771000 0.05608100 0.15753800<br>C -1.49177400 3.63019200 0.09710300<br>C -1.72305600 -3.53095000 -0.17226000<br>C 4.30553200 0.39078600 -0.69193400<br>H 2.15391300 -0.95818300 -1.72392200<br>H 2.03405800 0.76870200 -2.07045100<br>H 0.27740300 -2.11977000 -0.82965600<br>H 0.38772900 2.15990400 -0.67574900<br>H -3.75906000 -0.81092600 0.70156900<br>H -1.21391400 3.77753100 -0.94920200<br>H -0.60254000 3.68568200 0.72944400<br>H -2.20414600 4.39473000 0.39837100<br>H -0.84669500 -3.69751100 0.45808700<br>H -1.44698100 -3.61562100 -1.22566000<br>H -2.49242500 -4.26137600 0.06639900<br>H 4.79547200 -0.52630800 -0.99683400<br>H 4.78082800 1.34140800 -0.89042700<br>C 2.91365000 0.33217800 -0.18292400<br>O 2.75270800 -0.73947000 0.76412000<br>H 2.63233600 1.26788600 0.31212800<br>O 3.55212300 -0.46384800 1.90471600<br>H 2.94065100 0.01319900 2.48684700	• Electronic Energy = -804.058428<br>• Zero Point Energy Coorection = 0.249105<br>• Thermal Correction to Energy = 0.266832<br>• Thermal Correction to Enthalpy = 0.267776<br>• Thermal Correction to Free Energy = 0.202357

Name	Anionic HAs RAF 2'-C···•OOH Product
Cartesian Coordinate:	Thermochemical Values:

O 2.25858800 -2.34236200 0.29472300<br>O 2.23882400 2.35027100 0.10303100<br>O 3.46324600 0.02672800 0.67906800<br>C -0.43109500 -0.04856800 -0.81111200<br>C -1.86043700 -0.08465500 -1.29329200<br>C 0.22583800 1.16494600 -0.60723400<br>C 0.23848500 -1.23786400 -0.51368700<br>C 1.53877200 -1.20788400 -0.01816800<br>C 1.52934400 1.18654100 -0.11507100<br>C 2.24542300 0.00437700 0.20904900<br>C 1.61992500 -3.59281800 0.09071800<br>C 1.58876100 3.57399900 -0.20153500<br>C -4.26334100 -0.49857500 -0.68074700<br>H -2.13959900 0.87024200 -1.74830600<br>H -1.99151700 -0.86015800 -2.05287100<br>H -0.29282900 2.08819200 -0.83597000<br>H -0.26866100 -2.18232900 -0.67401200<br>H 1.35742100 -3.73510100 -0.96143500<br>H 0.72007300 -3.68171900 0.70596700<br>H 2.33924300 -4.35289700 0.39011600<br>H 0.69042200 3.70699100 0.40798500<br>H 1.32133900 3.62631400 -1.26072500<br>H 2.30278000 4.36223500 0.03018700<br>H -4.78085500 0.40072500 -0.99333600<br>H -4.70820200 -1.46436800 -0.87728600<br>C -2.87268000 -0.39491400 -0.17581800<br>O -2.73915500 0.70299200 0.74489000<br>H -2.56958900 -1.31089800 0.34313600<br>O -3.52578700 0.43268400 1.89623300<br>H -2.89971900 -0.01724400 2.48437500	• Electronic Energy<br>= -803.584382<br>• Zero Point Energy Coorection<br>= 0.236633<br>• Thermal Correction to Energy<br>= 0.253831<br>• Thermal Correction to Enthalpy<br>= 0.254775<br>• Thermal Correction to Free Energy<br>= 0.189027

Name	HAs RAF 3'-C···•OOH Product
Cartesian Coordinate:	Thermochemical Values:
O -0.73327900 2.76858400 -0.14046300<br>O -3.06106200 -1.21389500 0.39505900<br>O -2.90607600 1.43985000 0.57515600<br>C 0.36753200 -0.68417400 -0.86882000<br>C 1.59488000 -1.46188700 -1.31894200<br>C -0.78197400 -1.37303300 -0.48388300<br>C 0.41649100 0.70699300 -0.77363600<br>C -0.68310300 1.41430900 -0.28145900<br>C -1.87961800 -0.66217500 -0.00514500<br>C -1.83455600 0.72868100 0.10192400<br>C 2.37764900 -1.94806300 -0.13982100<br>C 0.45311100 3.49638900 -0.44757300<br>C -3.18159000 -2.63229700 0.31793500<br>H 1.27025500 -2.31161400 -1.92683400<br>H 2.22248000 -0.81727800 -1.94035800<br>H -0.81260300 -2.45283000 -0.55905300<br>H 1.31157700 1.23022000 -1.08653000<br>H 1.99798200 -2.78033100 0.44022600<br>H -3.61005900 0.81359500 0.79586100<br>H 0.71386600 3.38771800 -1.50278700<br>H 1.28428700 3.16074500 0.17810900<br>H 0.22701300 4.53803000 -0.23187600<br>H -2.43787100 -3.11645500 0.95465600<br>H -3.06859800 -2.97209200 -0.71390800<br>H -4.18140600 -2.86653100 0.67546600<br>C 3.57451900 -1.20845800 0.33142200<br>O 3.33056500 0.19635500 0.55179900<br>H 4.35377100 -1.17298200 -0.44007800<br>H 3.99376500 -1.64148900 1.24161800<br>O 2.44593000 0.33980600 1.65563400<br>H 1.58687600 0.49800300 1.22599100	• Electronic Energy<br>= -804.062950<br>• Zero Point Energy Coorection<br>= 0.251305<br>• Thermal Correction to Energy<br>= 0.268230<br>• Thermal Correction to Enthalpy<br>= 0.269174<br>• Thermal Correction to Free Energy<br>= 0.205802

Name	Anionic HAs RAF 3'-C···•OOH Product
Cartesian Coordinate:	Thermochemical Values:
O -0.99869800 2.78568600 0.00919000<br>O -2.74465900 -1.55904800 0.37181800<br>O -2.93261800 1.09030800 0.78432000<br>C 0.44086200 -0.42633800 -1.14003300<br>C 1.72510400 -0.99718600 -1.71763800<br>C -0.57370700 -1.28347100 -0.70887500<br>C 0.31283300 0.94361600 -0.91104800<br>C -0.81085900 1.44431000 -0.25563100<br>C -1.69915800 -0.77360400 -0.06788600<br>C -1.87259900 0.61394100 0.18953000<br>C 2.66115900 -1.40593900 -0.62051500<br>C 0.07604300 3.66017800 -0.29559700<br>C -2.62736300 -2.96036100 0.18244200<br>H 1.50020000 -1.85665700 -2.35244600	• Electronic Energy<br>= -803.585786<br>• Zero Point Energy Coorection<br>= 0.238259<br>• Thermal Correction to Energy<br>= 0.254913<br>• Thermal Correction to Enthalpy<br>= 0.255857

H 2.19986000 -0.23440100 -2.34536500<br>H -0.46647300 -2.34949300 -0.87286700<br>H 1.10174400 1.60978200 -1.23948500<br>H 2.76646800 -2.45013200 -0.34802600<br>H 0.27803900 3.68255400 -1.37000500<br>H 0.98407300 3.36605100 0.23992700<br>H -0.23517800 4.65007700 0.03327700<br>H -1.75372200 -3.35741600 0.70705900<br>H -2.55953300 -3.21123000 -0.87990300<br>H -3.53137900 -3.39934400 0.60059600<br>C 3.35605800 -0.38989600 0.20549200<br>O 3.07804700 -0.47611000 1.61813500<br>H 3.12610400 0.62629800 -0.13426800<br>H 4.44408900 -0.52320800 0.19218000<br>O 1.68520900 -0.30628000 1.84293300<br>H 1.51722500 0.61916400 1.59672100	• Thermal Correction to Free Energy = 0.192287

Name	HPns (4-propenylsyringol)
Cartesian Coordinate:	Thermochemical Values:
O 2.44300500 -1.83699700 -0.00017200<br>O 1.10112600 2.60810300 0.00005000<br>O 3.00603800 0.74589300 -0.00029000<br>C -0.98840700 -0.44098600 0.00030800<br>C 1.37486900 -0.98840700 -0.00002500<br>C 0.66224900 1.31544300 0.00008300<br>C -0.66958100 0.92274200 0.00030900<br>C 0.03948200 -1.38912900 0.00018100<br>C 1.69039700 0.36813100 -0.00009100<br>C -2.37967400 -0.93114000 0.00043900<br>C -3.49248700 -0.19234900 -0.00046200<br>C 2.16468400 -3.23432400 -0.00002100<br>C 0.11527900 3.63699900 0.00005900<br>C -4.87467300 -0.76612300 -0.00023600<br>H -1.45105100 1.67019700 0.00054500<br>H -0.21600800 -2.44073100 0.00022300<br>H -2.47981900 -2.01543500 0.00130200<br>H -3.42570100 0.89355300 -0.00148400<br>H 3.04674100 1.71278600 -0.00019800<br>H 1.60534000 -3.51881700 -0.89437300<br>H 1.60562300 -3.51867800 0.89455200<br>H 3.13279000 -3.72983600 -0.00013700<br>H -0.50793100 3.57336100 0.89488400<br>H -0.50810700 3.57319500 -0.89463400<br>H 0.66412100 4.57569400 -0.00008100<br>H -5.43460200 -0.42874800 0.87704700<br>H -5.43423300 -0.43032900 -0.87836000<br>H -4.85134200 -1.85734100 0.00075000	• Electronic Energy = -653.149950<br>• Zero Point Energy Coorection = 0.231740<br>• Thermal Correction to Energy = 0.246588<br>• Thermal Correction to Enthalpy = 0.247532<br>• Thermal Correction to Free Energy = 0.189452

Name	HPns 1-OH radical
Cartesian Coordinate:	Thermochemical Values:
O -2.58209000 -1.66655900 -0.00003100<br>O -0.90789400 2.70243800 0.00003900<br>O -2.90869900 0.96643400 0.00007300<br>C 0.91201800 -0.49912300 -0.00008400<br>C -1.47164900 -0.91352600 -0.00004100<br>C -0.58024900 1.39959600 0.00000400<br>C 0.69624100 0.90147200 -0.00005300<br>C -0.18293500 -1.39162600 -0.00009800<br>C -1.74571800 0.51837800 0.00001900<br>C 2.24701400 -1.07382600 -0.00010500<br>C 3.40442600 -0.39010000 0.00014200<br>C -2.41947300 -3.08550000 -0.00011500<br>C 0.15753500 3.65220600 0.00008800<br>C 4.74852900 -1.03466300 0.00012000<br>H 1.54342000 1.57255200 -0.00009000<br>H 0.01795300 -2.45514200 -0.00014000<br>H 2.28691700 -2.16076100 -0.00030900<br>H 3.39434000 0.69687200 0.00038500<br>H -1.88327700 -3.40706800 0.89519700<br>H -1.88332000 -3.40696600 -0.89548900<br>H -3.42483200 -3.49831100 -0.00011500<br>H 0.77231500 3.53539500 -0.89503700<br>H 0.77234500 3.53526500 0.89517500<br>H -0.31704600 4.62998400 0.00016500<br>H 5.32177800 -0.71849500 -0.87673400<br>H 5.32164800 -0.71883500 0.87718100<br>H 4.67278900 -2.12272900 -0.00009700	• Electronic Energy = -652.520688<br>• Zero Point Energy Coorection = 0.220319<br>• Thermal Correction to Energy = 0.234377<br>• Thermal Correction to Enthalpy = 0.235321<br>• Thermal Correction to Free Energy = 0.178986

Name	HPns 3'-CH radical
Cartesian Coordinate:	Thermochemical Values:
O 2.51667600 -1.68205200 -0.00011700<br>O 0.85650000 2.65374300 0.00003700<br>O 2.88372400 0.92970900 -0.00026300<br>C -1.01704000 -0.54277600 0.00021800<br>C 1.38616100 -0.92009900 -0.00000900<br>C 0.50720900 1.33488200 0.00006000<br>C -0.78996200 0.85088500 0.00023400<br>C 0.09109600 -1.41771500 0.00015000<br>C 1.60235100 0.46135200 -0.00009200<br>C -2.33387500 -1.11150900 0.00025300<br>C -3.55076300 -0.39943800 -0.00029200<br>C 2.34600100 -3.09661600 0.00001200<br>C -0.19727000 3.61333800 0.00009000<br>C -4.77219500 -1.00322100 -0.00020900<br>H -1.62156700 1.54086700 0.00050800<br>H -0.08852700 -2.48489900 0.00017500<br>H -2.39145400 -2.19682600 0.00065400<br>H -3.52017100 0.68685400 -0.00094400<br>H 2.85703200 1.89727100 -0.00025000<br>H 1.80987900 -3.42242400 -0.89448800<br>H 1.81005600 -3.42228100 0.89467000<br>H 3.34907400 -3.51678400 -0.00005300<br>H -0.81443300 3.50638800 0.89498700<br>H -0.81464600 3.50625200 -0.89464300<br>H 0.28629400 4.58720100 -0.00004000<br>H -5.68601300 -0.42247300 -0.00071300<br>H -4.86101000 -2.08484200 0.00038800	• Electronic Energy = -652.509606<br>• Zero Point Energy Coorection = 0.218860<br>• Thermal Correction to Energy = 0.233160<br>• Thermal Correction to Enthalpy = 0.234104<br>• Thermal Correction to Free Energy = 0.177225

Name	HPns 3'-CH radical anion
Cartesian Coordinate:	Thermochemical Values:
O 2.63188400 -1.55452400 0.00007800<br>O 0.72163300 2.73325100 -0.00007100<br>O 2.84945700 1.11098100 0.00001000<br>C -0.96166700 -0.58180100 0.00001100<br>C 1.46380000 -0.82924500 0.00004700<br>C 0.47777100 1.38061200 -0.00003300<br>C -0.78486000 0.82929300 -0.00003200<br>C 0.20675200 -1.39234500 0.00005100<br>C 1.67394800 0.58794700 0.00000800<br>C -2.23355400 -1.20770500 0.00001600<br>C -3.49416900 -0.55987900 -0.00005000<br>C 2.51601500 -2.96956000 0.00011600<br>C -0.40592100 3.59634900 -0.00011400<br>C -4.69205600 -1.20491100 -0.00003000<br>H -1.65340100 1.47344400 -0.00005800<br>H 0.08181600 -2.46836700 0.00008200<br>H -2.23953900 -2.29564500 0.00007300<br>H -3.50928300 0.52775200 -0.00012900<br>H 1.99202300 -3.32074700 -0.89310300<br>H 1.99201000 -3.32069800 0.89334600<br>H 3.53328300 -3.35611100 0.00013300<br>H -1.01770800 3.44303500 0.89314000<br>H -1.01768400 3.44297800 -0.89337400<br>H -0.01026300 4.61008000 -0.00014100<br>H -5.62596500 -0.65649600 -0.00009100<br>H -4.74541900 -2.28942400 0.00004900	• Electronic Energy = -652.041649<br>• Zero Point Energy Coorection = 0.206686<br>• Thermal Correction to Energy = 0.220355<br>• Thermal Correction to Enthalpy = 0.221299<br>• Thermal Correction to Free Energy = 0.165761

Name	HPns HT 1-OH···•OOH Transition State
Cartesian Coordinate:	Thermochemical Values:
O -1.57340900 2.44091000 -0.33935000<br>O -1.10602500 -2.16985700 -0.75424800<br>C 1.47453900 0.37477900 -0.11344000<br>C -0.70533900 1.40691500 -0.32968400<br>C -0.44708900 -1.00855500 -0.55595600<br>C 0.91443700 -0.89368500 -0.33164900<br>C 0.66099100 1.51929200 -0.11808000<br>C -1.28243200 0.13821800 -0.56613800<br>C 2.90985500 0.56617100 0.13018300<br>C 3.83045500 -0.39738500 0.24983700<br>C -1.04493600 3.74622500 -0.10705300<br>C -0.35664800 -3.38016200 -0.64885100<br>C 5.27940800 -0.13691100 0.50060300<br>H 1.54080700 -1.77425300 -0.32759000<br>H 1.11643700 2.48499400 0.05663500<br>H 3.22638700 1.60249100 0.22677500<br>H 3.53923200 -1.44195500 0.16725600<br>H -0.32263100 4.01117500 -0.88192400	• Electronic Energy = -804.044873<br>• Zero Point Energy Coorection = 0.244361<br>• Thermal Correction to Energy = 0.261807<br>• Thermal Correction to Enthalpy = 0.262751<br>• Thermal Correction to Free Energy = 0.198090

H -0.57618500 3.80089200 0.87769700<br>H -1.89635600 4.42081100 -0.14909100<br>H 0.09868200 -3.46147700 0.34069400<br>H 0.41339800 -3.42479800 -1.42184900<br>H -1.07302300 -4.18458000 -0.79507000<br>H 5.60176900 -0.62378600 1.42580800<br>H 5.88886000 -0.55939100 -0.30391100<br>H 5.48659700 0.93169100 0.57432000<br>O -2.58631600 0.02627100 -0.82926300<br>H -3.05243700 -0.47012300 -0.03595300<br>O -3.31662700 -0.96955100 1.26622000<br>O -2.13783500 -0.74621700 1.88082900<br>H -1.62928700 -1.57355500 1.79407200	

Name	HPns HT 3'-CH···•OOH Transition State
Cartesian Coordinate:	Thermochemical Values:
O 2.97301200 -1.95323200 0.22482000<br>O 1.89792500 2.56030600 0.08175300<br>O 3.64993100 0.59005600 0.41212500<br>C -0.30295600 -0.36153800 -0.44888100<br>C 1.97325500 -1.04888800 0.02834600<br>C 1.39718000 1.29950500 -0.05068500<br>C 0.07817900 0.98540900 -0.33761600<br>C 0.65144500 -1.37187700 -0.26028100<br>C 2.35097100 0.29058200 0.13216600<br>C -1.66916500 -0.76482400 -0.75929600<br>C -2.71054000 0.05126700 -1.06334000<br>C 2.62893900 -3.33262500 0.12485800<br>C 0.99503500 3.64856300 -0.10213400<br>H -0.64797400 1.77550200 -0.46872500<br>H 0.34544600 -2.40630000 -0.34731800<br>H -1.85165900 -1.83793000 -0.75425600<br>H -2.56726000 1.12803200 -1.08486400<br>H 3.74406100 1.55307400 0.44243800<br>H 2.25590900 -3.56735900 -0.87480800<br>H 1.88083500 -3.60069800 0.87441700<br>H 3.54852500 -3.88212400 0.31183000<br>H 0.19091800 3.60923300 0.63603000<br>H 0.57803300 3.63462400 -1.11154300<br>H 1.58361200 4.55136200 0.04159100<br>H -4.58794100 -0.38848900 -0.18378900<br>O -4.87870500 -0.35428600 1.14042900<br>O -3.71274200 0.11827600 1.68669300<br>H -3.15485600 -0.66609800 1.81445000<br>C -4.04995700 -0.44282200 -1.29706500<br>H -4.68876500 0.19223900 -1.90840000<br>H -4.11856500 -1.50054500 -1.55004400	• Electronic Energy<br>= -804.033828<br>• Zero Point Energy Coorection<br>= 0.243970<br>• Thermal Correction to Energy<br>= 0.261492<br>• Thermal Correction to Enthalpy<br>= 0.262436<br>• Thermal Correction to Free Energy<br>= 0.195654

Name	Anionic HPns HT 3'-CH···•OOH Transition State
Cartesian Coordinate:	Thermochemical Values:
O 3.14872000 -1.75961700 0.31125000<br>O 1.66506700 2.67587200 0.01752300<br>O 3.56459300 0.86511700 0.42211400<br>C -0.23468900 -0.46297600 -0.47150800<br>C 2.08436300 -0.95310600 0.06774500<br>C 1.30353000 1.37250500 -0.09035500<br>C 0.04291100 0.93214300 -0.38922700<br>C 0.82003900 -1.38779600 -0.23024800<br>C 2.40608400 0.45777400 0.15166700<br>C -1.52484200 -0.97808700 -0.77944200<br>C -2.64408700 -0.24040600 -1.09683500<br>C 2.92789300 -3.16468300 0.24697800<br>C 0.65390200 3.65439000 -0.19919100<br>H -0.75192500 1.64556200 -0.55669400<br>H 0.59464200 -2.44507100 -0.29118400<br>H -1.62559200 -2.06139300 -0.75930900<br>H -2.55398100 0.84180000 -1.15466500<br>H 2.59870000 -3.45859200 -0.75268500<br>H 2.18583900 -3.47365900 0.98711700<br>H 3.88558900 -3.62899200 0.47029400<br>H -0.15317300 3.54425700 0.52925200<br>H 0.25207800 3.57732700 -1.21246200<br>H 1.13856500 4.61898400 -0.06775800<br>H -4.41226600 -0.72538700 -0.05323000<br>O -4.69403000 -0.61799500 1.26666500<br>O -3.79238200 0.44878500 1.59531300<br>H -2.92221700 0.02660800 1.59424000<br>C -3.95740800 -0.78457500 -1.25755800<br>H -4.64251900 -0.18161900 -1.85147100	• Electronic Energy<br>= -803.577206<br>• Zero Point Energy Coorection<br>= 0.231165<br>• Thermal Correction to Energy<br>= 0.248405<br>• Thermal Correction to Enthalpy<br>= 0.249349<br>• Thermal Correction to Free Energy<br>= 0.183517

H -3.99565400 -1.84822100 -1.49653000	

Name	HPns RAF 2'-C···•OOH Transition State
Cartesian Coordinate:	Thermochemical Values:
O -2.90402500 -1.92999700 0.14945400<br>O -1.77314200 2.57102500 0.05765500<br>O -3.55373900 0.61888400 0.33326700<br>C 0.41126300 -0.37477500 -0.41590600<br>C -1.88699700 -1.03831100 -0.01093700<br>C -1.28171600 1.30551600 -0.06276800<br>C 0.04085200 0.97790900 -0.31325900<br>C -0.56205500 -1.37582700 -0.25839100<br>C -2.25158000 0.30630000 0.08953700<br>C 1.77693900 -0.78870600 -0.67164800<br>C -2.57788400 -3.31272200 0.03617800<br>C -0.85555500 3.65037200 -0.10547700<br>C 4.19339000 -0.49249000 -1.25645100<br>H 0.77857400 1.76045100 -0.42406500<br>H -0.26386500 -2.41319600 -0.33673800<br>H 1.95401500 -1.86213200 -0.67319000<br>H -3.63741600 1.58266000 0.37229100<br>H -1.85729000 -3.60496200 0.80338100<br>H -2.17758200 -3.53591200 -0.95561400<br>H -3.51095900 -3.85087800 0.18538500<br>H -0.41408900 3.63058400 -1.10434400<br>H -0.07032500 3.60467900 0.65237300<br>H -1.43894200 4.55891000 0.02226700<br>H 4.29181600 -0.41748800 -2.34398900<br>H 5.00249100 0.09019900 -0.81294800<br>H 4.30923000 -1.54034600 -0.97526300<br>C 2.85959000 0.03651600 -0.83774500<br>O 3.24039300 0.43200700 1.17110300<br>H 2.70282300 1.10239300 -0.96585500<br>O 3.96814000 -0.60200400 1.69939300<br>H 3.31302300 -1.18696200 2.11053600	• Electronic Energy<br>= -804.044296<br>• Zero Point Energy Coorection<br>= 0.248340<br>• Thermal Correction to Energy<br>= 0.265826<br>• Thermal Correction to Enthalpy<br>= 0.266771<br>• Thermal Correction to Free Energy<br>= 0.201400

Name	Anionic HPns RAF 2'-C···•OOH Transition State
Cartesian Coordinate:	Thermochemical Values:
O -3.07824400 -1.63178300 0.17703900<br>O -1.35071300 2.71018300 -0.15546700<br>O -3.36369700 1.01183200 0.16370500<br>C 0.41708400 -0.53372600 -0.37318500<br>C -1.95829700 -0.89022500 -0.00466400<br>C -1.04735600 1.38932600 -0.18302900<br>C 0.20795400 0.87622900 -0.36314800<br>C -0.69843900 -1.39727300 -0.18231300<br>C -2.21116800 0.53825700 0.00385900<br>C 1.70208400 -1.12170400 -0.56584900<br>C -2.92863900 -3.04831600 0.17668200<br>C -0.27839700 3.63084100 -0.33034500<br>C 4.13309500 -1.13774100 -1.18477700<br>H 1.05237100 1.53902500 -0.49216500<br>H -0.52323400 -2.46559900 -0.18599700<br>H 1.74568800 -2.20736400 -0.51648800<br>H -2.26897400 -3.36677600 0.98737200<br>H -2.53317800 -3.39397300 -0.78141700<br>H -3.92549600 -3.45409900 0.33185800<br>H 0.19705600 3.48880400 -1.30394600<br>H 0.46244200 3.51613900 0.46462400<br>H -0.72369900 4.62155000 -0.27888100<br>H 4.20326100 -1.14877000 -2.27801800<br>H 5.01316000 -0.61558300 -0.80625400<br>H 4.14618400 -2.17005000 -0.83103200<br>C 2.87906600 -0.44095800 -0.77320400<br>O 3.66842800 0.01235700 1.21305100<br>H 2.84863500 0.62746800 -0.95185100<br>O 2.51404600 0.18546000 2.05424300<br>H 1.88531200 -0.46342400 1.70145100	• Electronic Energy<br>= -803.587521<br>• Zero Point Energy Coorection<br>= 0.235896<br>• Thermal Correction to Energy<br>= 0.253046<br>• Thermal Correction to Enthalpy<br>= 0.253990<br>• Thermal Correction to Free Energy<br>= 0.190296

Name	HPns HT 1-OH···•OOH Reactant Complex
Cartesian Coordinate:	Thermochemical Values:

O -1.19066200 2.82139200 -0.13813300<br>O -1.46055300 -1.72698700 -1.02944900<br>C 1.50175000 0.27565900 -0.10377900<br>C -0.49280000 1.65290000 -0.23059300<br>C -0.61827600 -0.71234200 -0.68136300<br>C 0.74222800 -0.85101900 -0.44203400<br>C 0.87725500 1.52392800 -0.00673700<br>C -1.24703600 0.53037900 -0.56483500<br>C 2.95244700 0.20600000 0.15391800<br>C 3.71598600 -0.88991700 0.14643200<br>C -0.45856800 3.98836200 0.22503800<br>C -1.00588900 -3.05969500 -0.80872200<br>C 5.18816300 -0.88453700 0.41435500<br>H 1.20588400 -1.82438800 -0.52871200<br>H 1.47196900 2.39001100 0.25345600<br>H 3.42389500 1.16299500 0.37181100<br>H 3.27146000 -1.86018100 -0.06453400<br>H 0.31331300 4.20885000 -0.51591600<br>H -0.00445800 3.87010500 1.21163400<br>H -1.18379400 4.79845000 0.25030600<br>H -0.67917300 -3.18591500 0.22746100<br>H -0.18876700 -3.30957700 -1.48872600<br>H -1.85821200 -3.70463600 -1.01018200<br>H 5.42918200 -1.51990800 1.27176300<br>H 5.73860000 -1.28867600 -0.44048100<br>H 5.55119000 0.12482700 0.61591200<br>O -2.59371500 0.65935700 -0.75904600<br>H -2.96891300 -0.22868900 -0.85512200<br>O -3.18767800 -1.73159200 1.32301300<br>O -2.19469100 -1.09689100 1.87684500<br>H -1.43534900 -1.71380000 1.90538600	• Electronic Energy = -804.059685<br>• Zero Point Energy Coorection = 0.248976<br>• Thermal Correction to Energy = 0.267568<br>• Thermal Correction to Enthalpy = 0.268513<br>• Thermal Correction to Free Energy = 0.201017

Name	HPns HT 3'-CH···•OOH Reactant Complex
Cartesian Coordinate:	Thermochemical Values:
O 2.89581600 -1.91873300 0.18207600<br>O 1.74092900 2.57562100 0.02669300<br>O 3.54076500 0.63783500 0.33461400<br>C -0.41522800 -0.38840300 -0.44129800<br>C 1.87818700 -1.03034800 -0.00251600<br>C 1.26430500 1.30262900 -0.09129100<br>C -0.05795300 0.96172100 -0.34677100<br>C 0.55290500 -1.37961900 -0.25568800<br>C 2.23722700 0.31399400 0.07871800<br>C -1.79965900 -0.81168500 -0.72672200<br>C -2.77081300 -0.05094400 -1.24781500<br>C 2.57283400 -3.30375300 0.09483300<br>C 0.81049800 3.64315600 -0.13765400<br>H -0.80600800 1.73541900 -0.45716000<br>H 0.26222700 -2.42007600 -0.32068100<br>H -2.02299000 -1.85307700 -0.49957900<br>H -2.55923800 0.98162200 -1.51962000<br>H 3.61566200 1.60221600 0.36170000<br>H 2.18127200 -3.54886400 -0.89525300<br>H 1.84655000 -3.58204300 0.86187300<br>H 3.50549400 -3.83728200 0.26275400<br>H 0.02498000 3.58874300 0.61942000<br>H 0.36972300 3.61845900 -1.13673700<br>H 1.38223400 4.55912400 -0.00955800<br>H -4.88656900 0.07068200 -0.93460200<br>O -3.94023900 -0.66859500 1.91813500<br>O -3.24346600 0.42219700 1.78322300<br>H -2.79484200 0.35116500 0.90618700<br>C -4.16580600 -0.53150300 -1.49648600<br>H -4.42475300 -0.42721300 -2.55406100<br>H -4.28316800 -1.57694400 -1.20647500	• Electronic Energy = -804.061220<br>• Zero Point Energy Coorection = 0.248877<br>• Thermal Correction to Energy = 0.267358<br>• Thermal Correction to Enthalpy = 0.268303<br>• Thermal Correction to Free Energy = 0.200590

Name	Anionic HPns HT 3'-CH···•OOH Reactant Complex
Cartesian Coordinate:	Thermochemical Values:
O 2.88146100 -1.90487900 0.15441900<br>O 1.71672800 2.62668200 0.10914900<br>O 3.47834600 0.66849600 0.40093500<br>C -0.37616500 -0.33327000 -0.53707200<br>C 1.88581700 -1.02629600 -0.03598000<br>C 1.26597100 1.37350800 -0.06596500<br>C -0.02277800 1.03009200 -0.38099100<br>C 0.58721400 -1.34906200 -0.34933900<br>C 2.30365200 0.36197600 0.12017900<br>C -1.72955300 -0.74378300 -0.87564200<br>C -2.74258700 0.06934000 -1.22536700	• Electronic Energy = -803.597041<br>• Zero Point Energy Coorection = 0.235327<br>• Thermal Correction to Energy = 0.253814

C 2.57187200 -3.29272500 0.02243700<br>C 0.78045800 3.69271600 -0.04840400<br>H -0.77669700 1.79525400 -0.50037700<br>H 0.28078300 -2.38046700 -0.46591900<br>H -1.91724300 -1.81436000 -0.83736700<br>H -2.58296600 1.14261300 -1.29933300<br>H 2.22094500 -3.50950300 -0.98863900<br>H 1.81572500 -3.58513400 0.75376100<br>H 3.50080700 -3.82276900 0.21570100<br>H -0.02770400 3.60113800 0.68032800<br>H 0.37373400 3.69623300 -1.06188800<br>H 1.33994200 4.60719300 0.13065400<br>H -4.83118500 0.04470300 -0.81295000<br>O -3.64027700 -1.07482900 1.92618900<br>O -3.24861900 0.33396400 1.94522400<br>H -2.66822100 0.41148100 1.17732600<br>C -4.12351700 -0.41191600 -1.51282000<br>H -4.43566400 -0.10843300 -2.51663700<br>H -4.19567500 -1.49690200 -1.42821200	• Thermal Correction to Enthalpy = 0.254758<br>• Thermal Correction to Free Energy = 0.186564

Name	HPns RAF 2’-C···•OOH Reactant Complex
Cartesian Coordinate:	Thermochemical Values:
O -3.07696400 -1.55238800 0.26976300<br>O -1.18600400 2.64411600 -0.34289100<br>O -3.28930000 1.07703500 0.11630500<br>C 0.45340700 -0.66624400 -0.40405100<br>C -1.92297600 -0.86922500 0.02260200<br>C -0.92707300 1.30488800 -0.30246500<br>C 0.32246600 0.72561600 -0.47739100<br>C -0.67013300 -1.45709000 -0.14591500<br>C -2.05426700 0.51556500 -0.05588200<br>C 1.75394300 -1.34180100 -0.57369000<br>C -2.98704200 -2.97177400 0.35465600<br>C -0.07948800 3.51896400 -0.54814300<br>C 4.19487500 -1.51677400 -1.10212000<br>H 1.18724900 1.34806600 -0.66101300<br>H -0.55561700 -2.53150500 -0.08316900<br>H 1.75046900 -2.40929600 -0.35845700<br>H -3.20376600 2.03633000 0.02111900<br>H -2.33177800 -3.27083300 1.17606800<br>H -2.62328900 -3.39429100 -0.58476700<br>H -3.99846300 -3.32227200 0.54684700<br>H 0.38968700 3.32590600 -1.51547400<br>H 0.65498100 3.40350500 0.25264500<br>H -0.48822000 4.52639400 -0.53137500<br>H 4.57574200 -1.44598700 -2.12502600<br>H 4.95669000 -1.07692400 -0.45117100<br>H 4.07904300 -2.57040000 -0.84267600<br>C 2.90163900 -0.77681800 -0.97022600<br>O 2.86550100 1.19891400 1.63641500<br>H 2.92682600 0.28252700 -1.21682500<br>O 2.70228900 -0.00432900 2.10415300<br>H 2.64417300 -0.59983100 1.31906100	• Electronic Energy = -804.061631<br>• Zero Point Energy Coorection = 0.248205<br>• Thermal Correction to Energy = 0.267022<br>• Thermal Correction to Enthalpy = 0.267967<br>• Thermal Correction to Free Energy = 0.198521

Name	Anionic HPns RAF 2’-C···•OOH Reactant Complex
Cartesian Coordinate:	Thermochemical Values:
O -3.16392400 -1.47051200 0.38859800<br>O -1.17897500 2.70827900 -0.32102200<br>O -3.26728000 1.17855400 0.25145000<br>C 0.31815100 -0.65189800 -0.48960300<br>C -2.02631600 -0.82961600 0.07996600<br>C -0.97227100 1.38156700 -0.29818100<br>C 0.22372600 0.76134500 -0.55010700<br>C -0.81698400 -1.43123100 -0.17186000<br>C -2.17569200 0.62012700 0.02921700<br>C 1.56762500 -1.35496700 -0.73106500<br>C -3.12136500 -2.89629000 0.45754700<br>C -0.05961600 3.54530100 -0.61221500<br>C 3.99607400 -1.57866000 -1.28613300<br>H 1.09886600 1.34670100 -0.79520300<br>H -0.70987600 -2.50738400 -0.13163900<br>H 1.51698100 -2.43738500 -0.63935700<br>H -2.42249800 -3.21925000 1.23186200<br>H -2.83172200 -3.31522200 -0.50837100<br>H -4.13017800 -3.21032900 0.71222200<br>H 0.32870100 3.32981900 -1.60994800<br>H 0.72511600 3.40480300 0.13450100<br>H -0.43372400 4.56491400 -0.57126700<br>H 4.36412500 -1.40360200 -2.30192000	• Electronic Energy = -803.596917<br>• Zero Point Energy Coorection = 0.235613<br>• Thermal Correction to Energy = 0.254078<br>• Thermal Correction to Enthalpy = 0.255022<br>• Thermal Correction to Free Energy = 0.186878

H 4.78628300 -1.24853900 -0.60537600<br>H 3.83123400 -2.64806400 -1.14869000<br>C 2.75014500 -0.79609100 -1.04758100<br>O 3.86416800 0.05939000 1.99818400<br>H 2.83649700 0.28334100 -1.14757600<br>O 2.60681800 0.80143100 1.90036900<br>H 2.05288200 0.22554000 1.36022900	

Name	HPns HT 1-OH···•OOH Product Complex
Cartesian Coordinate:	Thermochemical Values:
O -0.63451200 2.95112500 -0.10369800<br>O -1.49187700 -1.63673000 -0.45978100<br>C 1.71165600 0.11708600 0.01965700<br>C -0.08898900 1.72727700 -0.10443700<br>C -0.53921300 -0.70115300 -0.29285200<br>C 0.79799900 -0.95455300 -0.14871800<br>C 1.24919600 1.45089100 0.04368200<br>C -1.06362400 0.65979700 -0.28403300<br>C 3.13942300 -0.10014700 0.17086200<br>C 3.77075300 -1.28696400 0.13112100<br>C 0.24231900 4.06680400 0.06448100<br>C -1.07488600 -3.00246400 -0.55161800<br>C 5.24360200 -1.44653100 0.29017500<br>H 1.16105000 -1.97243600 -0.15996100<br>H 1.97032600 2.24679400 0.17651500<br>H 3.73371000 0.79757900 0.32372600<br>H 3.20439500 -2.20083300 -0.02786600<br>H 0.97031900 4.10368200 -0.74849500<br>H 0.75445000 4.00595800 1.02682800<br>H -0.39299400 4.94803400 0.03600800<br>H -0.58977400 -3.31551200 0.37525500<br>H -0.39706000 -3.13372400 -1.39712000<br>H -1.98491200 -3.57619700 -0.70894200<br>H 5.46452900 -2.10045000 1.13937100<br>H 5.67053600 -1.93025000 -0.59370200<br>H 5.73828400 -0.48668200 0.44325000<br>O -2.28072000 0.90926400 -0.42560000<br>H -3.65272900 -0.18301100 -0.25762300<br>O -4.29188100 -0.84576200 0.08424300<br>O -3.68813500 -1.21644100 1.31916900<br>H -2.88253600 -1.67484500 1.02816400	• Electronic Energy = -804.084180<br>• Zero Point Energy Coorection = 0.249380<br>• Thermal Correction to Energy = 0.267584<br>• Thermal Correction to Enthalpy = 0.268528<br>• Thermal Correction to Free Energy = 0.201918

Name	HPns HT 3'-CH···•OOH Product Complex
Cartesian Coordinate:	Thermochemical Values:
O 2.92064000 -1.74366800 0.22247900<br>O 1.40603900 2.63127000 -0.11127800<br>O 3.33709100 0.85898600 0.33208000<br>C -0.48727800 -0.51766900 -0.58910300<br>C 1.84029700 -0.95446400 -0.03386400<br>C 1.03730700 1.32279600 -0.21252600<br>C -0.23512800 0.87166700 -0.52065200<br>C 0.56989300 -1.41991000 -0.33773700<br>C 2.08225300 0.42182800 0.02952600<br>C -1.78357000 -1.05466000 -0.88216300<br>C -2.95289500 -0.31026300 -1.15425200<br>C 2.72139700 -3.15369300 0.16807000<br>C 0.39784800 3.61639900 -0.32491100<br>H -1.02707100 1.58382100 -0.70571600<br>H 0.37015300 -2.48230100 -0.38676900<br>H -1.87552900 -2.13746200 -0.86371200<br>H -2.88294100 0.77240800 -1.21111300<br>H 3.33096700 1.82705000 0.33849400<br>H 2.40106100 -3.46120000 -0.83007400<br>H 1.98410900 -3.46963100 0.90964000<br>H 3.68659000 -3.59915000 0.39733600<br>H -0.40789800 3.50509300 0.40432100<br>H -0.00155000 3.54387500 -1.33889000<br>H 0.88753700 4.57767700 -0.18948900<br>H -3.65494300 -0.74513800 1.12770500<br>O -3.28537600 -0.59804300 2.01533400<br>O -2.60539800 0.63974300 1.87104300<br>H -1.70878400 0.37452400 1.61122500<br>C -4.17792000 -0.88167900 -1.32549200<br>H -5.05500400 -0.27921200 -1.52583900<br>H -4.30387900 -1.95877800 -1.27574400	• Electronic Energy = -804.069862<br>• Zero Point Energy Coorection = 0.248156<br>• Thermal Correction to Energy = 0.266720<br>• Thermal Correction to Enthalpy = 0.267664<br>• Thermal Correction to Free Energy = 0.200128

Name	Anionic HPns HT 3’-CH···•OOH Product Complex
Cartesian Coordinate:	Thermochemical Values:
O 2.91324700 -1.79850400 0.16876300<br>O 1.50800700 2.67500400 0.01846000<br>O 3.39901800 0.82289800 0.37418700<br>C -0.45580300 -0.41873600 -0.60424000<br>C 1.86150400 -0.94631700 -0.05830200<br>C 1.13461900 1.36460300 -0.13802000<br>C -0.14102400 0.96382800 -0.46520000<br>C 0.59024200 -1.35943300 -0.38267500<br>C 2.21197100 0.43900700 0.07685200<br>C -1.75058600 -0.89436100 -0.92434300<br>C -2.90444000 -0.10367600 -1.16647900<br>C 2.65501000 -3.19042600 0.05785700<br>C 0.50282600 3.66288700 -0.15695900<br>H -0.91415100 1.70470400 -0.61682800<br>H 0.35703700 -2.41277300 -0.47885000<br>H -1.87875700 -1.97417100 -0.95797400<br>H -2.79254100 0.97805300 -1.17851300<br>H 2.32729900 -3.44913300 -0.95269400<br>H 1.89835700 -3.50773200 0.78052300<br>H 3.59694400 -3.69056900 0.27404800<br>H -0.30741300 3.52970500 0.56518900<br>H 0.09753000 3.63452700 -1.17199800<br>H 0.98889300 4.62128100 0.01407100<br>H -3.87241100 -0.70008000 0.98503600<br>O -3.45848200 -0.81213100 1.86062700<br>O -2.57114000 0.29421500 1.93767600<br>H -1.78577900 -0.01279500 1.45362400<br>C -4.15522700 -0.60714000 -1.35513900<br>H -5.00101000 0.04436900 -1.53718600<br>H -4.33375300 -1.67866900 -1.35625700	• Electronic Energy<br>= -803.603425<br>• Zero Point Energy Coorection<br>= 0.235686<br>• Thermal Correction to Energy<br>= 0.253715<br>• Thermal Correction to Enthalpy<br>= 0.254660<br>• Thermal Correction to Free Energy<br>= 0.188056

Name	HPns RAF 1-C···•OOH Product
Cartesian Coordinate:	Thermochemical Values:
O 2.04672200 -2.03324400 -0.26161100<br>O 0.88099600 2.50569600 -0.28346700<br>O 2.35731900 0.43400100 -1.33448800<br>C -1.31492400 -0.47365000 -0.09172500<br>C 1.01313600 -1.16372100 -0.21595900<br>C 0.38071800 1.25041500 -0.21573800<br>C -0.92446800 0.90368100 -0.14436900<br>C -0.30439200 -1.48035500 -0.13787000<br>C -2.69101000 -0.88419500 -0.00414400<br>C -3.77910100 -0.08409000 0.05684700<br>C 1.72841700 -3.42223900 -0.23039700<br>C -0.05492100 3.58109300 -0.31745500<br>C -5.17872400 -0.59827500 0.14409400<br>H -1.68379500 1.67426300 -0.13647400<br>H -0.61598200 -2.51734300 -0.11071800<br>H -2.85366800 -1.96067400 0.01402300<br>H -3.66223300 0.99644300 0.04503400<br>H 2.53684200 1.38177800 -1.42212900<br>H 1.11070600 -3.69168300 -1.09022700<br>H 1.20716500 -3.67353600 0.69632700<br>H 2.67855700 -3.94843500 -0.27647400<br>H -0.65284700 3.59313600 0.59666900<br>H -0.70688600 3.48980900 -1.18919600<br>H 0.53583600 4.49090700 -0.38690700<br>H -5.67092000 -0.23474700 1.05175100<br>H -5.77791200 -0.24028400 -0.69914500<br>H -5.20234100 -1.68937900 0.14888600<br>C 1.50539600 0.25258500 -0.23789400<br>O 2.40434700 0.49146900 0.88420600<br>O 1.68835800 0.32762400 2.09422600<br>H 1.45478300 1.23788800 2.33531700	• Electronic Energy<br>= -804.065367<br>• Zero Point Energy Coorection<br>= 0.250579<br>• Thermal Correction to Energy<br>= 0.267768<br>• Thermal Correction to Enthalpy<br>= 0.268712<br>• Thermal Correction to Free Energy<br>= 0.205619

Name	Anionic HPns RAF 1-C···•OOH Product
Cartesian Coordinate:	Thermochemical Values:
O 2.89296700 -1.78858300 -0.03666300<br>O 1.50412300 2.69614800 -0.01968900<br>O 3.43592900 0.83645000 0.05310100<br>C -0.53260800 -0.38917500 -0.20420400<br>C 1.82412000 -0.91834800 -0.07474100<br>C 1.11283200 1.37460500 -0.06839200<br>C -0.21445900 0.97075000 -0.15539500<br>C 0.49972400 -1.33016000 -0.16125500<br>C 2.19645600 0.45334600 -0.02326100	• Electronic Energy<br>= -803.589661<br>• Zero Point Energy Coorection<br>= 0.236764<br>• Thermal Correction to Energy

C -2.91322300 -0.04790800 -1.01750600<br>C 2.60043900 -3.17630200 -0.08559100<br>C 0.48072700 3.67807600 -0.05982100<br>C -4.34834300 -0.43703700 -1.06611100<br>H -1.00821600 1.70646300 -0.17950300<br>H 0.25034900 -2.38398100 -0.19899700<br>H -2.56829100 0.83972300 -1.52975000<br>H 2.08840900 -3.43899500 -1.01573000<br>H 1.98644600 -3.48028100 0.76685800<br>H 3.55916300 -3.68976200 -0.04276200<br>H -0.19722100 3.57703700 0.79252600<br>H -0.09128100 3.61447100 -0.98989000<br>H 0.98382100 4.64194400 -0.00964500<br>H -4.88663300 -0.12221200 -0.16026800<br>H -4.85740700 0.01654700 -1.91767700<br>H -4.45836600 -1.52518500 -1.12785800<br>C -1.95621100 -0.87298700 -0.23548300<br>O -2.47516200 -1.11575500 1.11714500<br>H -1.97679600 -1.90949500 -0.60166000<br>O -2.58444700 0.11589400 1.81775800<br>H -3.50799000 0.37133100 1.67170500	= 0.254073<br>• Thermal Correction to Enthalpy<br>= 0.255018<br>• Thermal Correction to Free Energy<br>= 0.190623

Name	HPns RAF 2-C···•OOH Product
Cartesian Coordinate:	Thermochemical Values:
O -2.10062500 2.31469100 -0.13496500<br>O -1.21718700 -2.06352600 -1.00375400<br>O -2.94177600 -0.18099900 -0.05396500<br>C 1.15951700 0.48560800 -0.24330400<br>C -1.13902900 1.34521500 -0.15015800<br>C 0.73037600 -0.80091200 -0.20860400<br>C 0.22557400 1.58151100 -0.21944500<br>C -1.61426800 0.04069900 -0.10590600<br>C 2.59313300 0.84028400 -0.30970900<br>C 3.59350600 0.08784900 0.15314000<br>C -1.65553700 3.66634700 -0.20851200<br>C -0.40645900 -3.21047200 -1.28459200<br>C 5.03836500 0.46267600 0.06640400<br>H 1.42972200 -1.62770100 -0.23923400<br>H 0.61155900 2.59089100 -0.26146700<br>H 2.81926000 1.80896700 -0.75176500<br>H 3.36538400 -0.86027900 0.63683400<br>H -3.11713100 -1.13258200 -0.11443700<br>H -1.10827000 3.84457900 -1.13718600<br>H -1.02520500 3.91433000 0.64866200<br>H -2.55551400 4.27659600 -0.19045600<br>H -0.05494400 -3.68014400 -0.36501600<br>H 0.44098900 -2.93572300 -1.91491000<br>H -1.04956900 -3.90327900 -1.82341400<br>H 5.48676600 0.50669600 1.06304800<br>H 5.59675100 -0.29103800 -0.49675100<br>H 5.17004100 1.43013500 -0.42097800<br>C -0.70754900 -1.14820400 -0.06744800<br>O -0.94847600 -1.88246000 1.18942700<br>O -0.49345600 -1.10849900 2.28284600<br>H -1.30343600 -0.67870700 2.59916300	• Electronic Energy<br>= -804.045670<br>• Zero Point Energy Coorection<br>= 0.249370<br>• Thermal Correction to Energy<br>= 0.266975<br>• Thermal Correction to Enthalpy<br>= 0.267919<br>• Thermal Correction to Free Energy<br>= 0.203429

Name	Anionic HPns RAF 2-C···•OOH Product
Cartesian Coordinate:	Thermochemical Values:
O -3.18532900 -1.56876900 0.15315000<br>O -1.31054000 2.71970100 -0.19653600<br>O -3.42121600 1.09260800 0.03337000<br>C 0.40011100 -0.58946200 -0.16439400<br>C -2.02480200 -0.84436300 0.03079200<br>C -1.05620200 1.37050900 -0.15279800<br>C 0.20584400 0.82823500 -0.21685000<br>C -0.77253500 -1.40333700 -0.03325100<br>C -2.24522100 0.57465400 -0.02651800<br>C 1.65408700 -1.19907200 -0.23671000<br>C -3.06213500 -2.98163100 0.22476800<br>C -0.19514100 3.58872900 -0.32588400<br>C 3.92689900 -1.29412300 -1.28027000<br>H 1.06730200 1.47626000 -0.29643600<br>H -0.64152600 -2.47753000 0.00953500<br>H 1.69729400 -2.28325800 -0.16417000<br>H -2.47050300 -3.27987800 1.09457200<br>H -2.60434100 -3.38240600 -0.68365400<br>H -4.07449100 -3.36849400 0.32314500<br>H 0.35010500 3.39175600 -1.25299300<br>H 0.48304800 3.48704100 0.52592300<br>H -0.60026200 4.59843300 -0.34934800<br>H 3.51279700 -1.40464100 -2.28347300	• Electronic Energy<br>= -803.613820<br>• Zero Point Energy Coorection<br>= 0.238495<br>• Thermal Correction to Energy<br>= 0.255075<br>• Thermal Correction to Enthalpy<br>= 0.256019<br>• Thermal Correction to Free Energy<br>= 0.193864

H 4.88922800 -0.78440900 -1.35440700<br>H 4.08138500 -2.28998500 -0.85797700<br>C 2.95428800 -0.50289100 -0.42084200<br>O 3.68413600 -0.33813900 0.83587200<br>H 2.82442700 0.50247300 -0.83208500<br>O 2.93217000 0.48787900 1.71091600<br>H 3.18060200 1.38491000 1.43893400	

Name	HPns RAF 3-C···•OOH Product
Cartesian Coordinate:	Thermochemical Values:
O 2.68299200 -1.81548100 -0.05868000<br>O 1.02718400 2.54389600 -0.20172900<br>O 3.07865900 0.76909300 -0.20981700<br>C -0.84653600 -0.73391300 -0.19303300<br>C 1.55627700 -1.07278600 -0.11546700<br>C 0.73615000 1.20377300 -0.22883000<br>C 0.25963200 -1.57366200 -0.13799100<br>C 1.77925400 0.34520200 -0.18656600<br>C -2.17363500 -1.29022100 -0.28644200<br>C -3.32968600 -0.59962600 -0.35933700<br>C 2.53440800 -3.23303600 0.01382300<br>C 0.17871400 3.39327100 -0.98266200<br>C -4.67433800 -1.24140200 -0.47646400<br>H 0.09376600 -2.64412900 -0.14016300<br>H -2.22248500 -2.37802600 -0.30631600<br>H -3.31400600 0.48703900 -0.33389700<br>H 3.07673100 1.73630900 -0.25538200<br>H 2.03684600 -3.61279900 -0.88108500<br>H 1.96852800 -3.51268500 0.90503700<br>H 3.54313000 -3.63371200 0.07406600<br>H -0.81369500 3.47005200 -0.53425700<br>H 0.10248200 3.01847800 -2.00584400<br>H 0.65296200 4.37219400 -0.98332900<br>H -5.31850700 -0.95007000 0.35911300<br>H -5.18227900 -0.91600300 -1.38974400<br>H -4.59604300 -2.32992400 -0.49180800<br>C -0.68131900 0.75647400 -0.11939400<br>O -1.27376400 1.30820800 1.09816800<br>H -1.30986700 1.24930300 -0.87087900<br>O -0.61798400 0.75787600 2.23001900<br>H 0.07222200 1.41079700 2.42498300	• Electronic Energy = -804.054145<br>• Zero Point Energy Coorection = 0.250392<br>• Thermal Correction to Energy = 0.267714<br>• Thermal Correction to Enthalpy = 0.268658<br>• Thermal Correction to Free Energy = 0.205343

Name	Anionic HPns RAF 3-C···•OOH Product
Cartesian Coordinate:	Thermochemical Values:
O -2.42216100 2.04214900 -0.03979400<br>O -1.14909500 -2.54261400 -0.03460600<br>O -3.10691400 -0.52408400 -0.04957100<br>C 0.95334700 0.56386600 -0.22448000<br>C -1.39782900 1.15851500 -0.08495400<br>C -0.83048800 -1.18278100 -0.04840100<br>C -0.06574300 1.51850800 -0.20036800<br>C -1.83970700 -0.25193500 -0.04770000<br>C 2.32146700 0.97029100 -0.39890400<br>C 3.40536500 0.16441500 -0.43487700<br>C -2.10852000 3.43020700 -0.09229000<br>C -1.39231800 -3.05479400 -1.34553500<br>C 4.80178700 0.65982700 -0.63490300<br>H 0.21261600 2.56205100 -0.29283600<br>H 2.47964200 2.04192800 -0.51730300<br>H 3.28357000 -0.90889400 -0.31305600<br>H -1.61486300 3.67934900 -1.03459500<br>H -1.46986100 3.71356600 0.74761600<br>H -3.05903100 3.95443100 -0.02563000<br>H -0.50631300 -2.93001500 -1.97571700<br>H -2.24220100 -2.54269000 -1.80472200<br>H -1.61737100 -4.11665500 -1.24355700<br>H 5.43901500 0.38678000 0.21238400<br>H 5.25454000 0.20765300 -1.52306500<br>H 4.82533300 1.74521100 -0.74934100<br>C 0.61847300 -0.88023200 -0.00474300<br>O 1.19940500 -1.35706800 1.27258000<br>H 1.17167300 -1.53300900 -0.69332900<br>O 0.64637500 -0.61432200 2.34883500<br>H -0.29262400 -0.86751800 2.33653000	• Electronic Energy = -803.582723<br>• Zero Point Energy Coorection = 0.236986<br>• Thermal Correction to Energy = 0.254089<br>• Thermal Correction to Enthalpy = 0.255033<br>• Thermal Correction to Free Energy = 0.191915

Name	HPns RAF 4-C···•OOH Product
Cartesian Coordinate:	Thermochemical Values:

O 2.47213000 -1.63700800 -0.69300000<br>O 0.93432600 2.73173400 -0.31441000<br>O 2.81989600 0.96268700 -0.86455300<br>C 1.40524000 -0.88286600 -0.31366300<br>C 0.57032300 1.43418300 -0.11682600<br>C -0.63752100 1.00050100 0.31484700<br>C 0.21269000 -1.36162500 0.12516000<br>C 1.62168100 0.52098400 -0.43173600<br>C -2.20156200 -0.94287400 -0.06753600<br>C -2.98208500 -0.25052100 -0.89306800<br>C 2.32522600 -3.05049900 -0.59066800<br>C -0.04442400 3.72866000 -0.03106600<br>C -4.23651100 -0.78812000 -1.50628300<br>H -1.43449700 1.68992200 0.55953300<br>H 0.02069900 -2.42388700 0.20627300<br>H -2.45601700 -1.96854100 0.19475300<br>H -2.70578900 0.76617100 -1.16360900<br>H 2.79832700 1.93134100 -0.90661900<br>H 1.51656700 -3.40162200 -1.23600900<br>H 2.12828300 -3.34264800 0.44363000<br>H 3.27083400 -3.47364800 -0.92093000<br>H -0.33832100 3.68672300 1.02002000<br>H -0.92058300 3.59508900 -0.66974500<br>H 0.42944200 4.68324900 -0.24612100<br>H -5.09151700 -0.15940300 -1.24268300<br>H -4.16329100 -0.78070400 -2.59756700<br>H -4.43652800 -1.80864900 -1.17592900<br>C -0.90427700 -0.45130200 0.53794900<br>O -1.17987300 -0.66690100 1.98173200<br>O -0.03363100 -0.33915400 2.74919300<br>H 0.50692800 -1.14338200 2.70064800	• Electronic Energy<br>= -804.046181<br>• Zero Point Energy Coorection<br>= 0.249056<br>• Thermal Correction to Energy<br>= 0.266656<br>• Thermal Correction to Enthalpy<br>= 0.267600<br>• Thermal Correction to Free Energy<br>= 0.203178

Name	Anionic HPns RAF 4-C⋯•OOH Product
Cartesian Coordinate:	Thermochemical Values:
O 2.51856700 -1.66555300 -0.63529900<br>O 0.93488500 2.74411800 -0.42352100<br>O 2.89926300 0.97684700 -0.80133800<br>C 1.45233900 -0.87075300 -0.30452300<br>C 0.62393700 1.43074100 -0.20074000<br>C -0.60892500 0.99743200 0.18285500<br>C 0.24211600 -1.34736200 0.09156100<br>C 1.74125000 0.54198600 -0.44561000<br>C -2.19337400 -0.96772700 -0.00130400<br>C -3.02649000 -0.34310200 -0.82969200<br>C 2.34120000 -3.06968000 -0.51315000<br>C -0.09183000 3.70156700 -0.20495700<br>C -4.31451400 -0.92474600 -1.32382800<br>H -1.42785800 1.68653700 0.34199200<br>H 0.04486400 -2.40858600 0.17470500<br>H -2.43201700 -1.97074200 0.35101500<br>H -2.76907500 0.64873500 -1.19511800<br>H 1.55211900 -3.42392900 -1.18167700<br>H 2.09835100 -3.34403900 0.51682600<br>H 3.28975900 -3.52032900 -0.79806600<br>H -0.43580500 3.67430700 0.83233100<br>H -0.93705800 3.52830000 -0.87623800<br>H 0.35095200 4.67230000 -0.41848500<br>H -5.15597200 -0.27959000 -1.05567100<br>H -4.31024500 -1.00040000 -2.41507300<br>H -4.48953600 -1.91768000 -0.90616900<br>C -0.86546900 -0.43452700 0.48730900<br>O -1.09541600 -0.55584100 1.98497600<br>O 0.07596300 -0.17484900 2.68864800<br>H 0.15398600 0.77437500 2.49436400	• Electronic Energy<br>= -803.585018<br>• Zero Point Energy Coorection<br>= 0.236879<br>• Thermal Correction to Energy<br>= 0.253830<br>• Thermal Correction to Enthalpy<br>= 0.254775<br>• Thermal Correction to Free Energy<br>= 0.192219

Name	HPns RAF 1’-C⋯•OOH Product
Cartesian Coordinate:	Thermochemical Values:
O 2.84766000 -1.82212500 -0.02530200<br>O 1.52110000 2.62843600 -0.05624600<br>O 3.41359100 0.75583700 0.01850300<br>C -0.56644900 -0.41299200 -0.16525200<br>C 1.78179800 -0.97291400 -0.06235600<br>C 1.07914500 1.33748500 -0.08305400<br>C -0.25446200 0.94651300 -0.14736000<br>C 0.44689800 -1.36987100 -0.12195100<br>C 2.09888200 0.38489600 -0.04012000<br>C -2.92424100 -0.10783400 -1.04995000<br>C 2.56649800 -3.21889000 -0.04973600<br>C 0.53647000 3.65863500 -0.08772700<br>C -4.36704700 -0.46677800 -1.08908200<br>H -1.04092000 1.68869300 -0.17834100	• Electronic Energy<br>= -804.061840<br>• Zero Point Energy Coorection<br>= 0.249450<br>• Thermal Correction to Energy<br>= 0.267161<br>• Thermal Correction to Enthalpy<br>= 0.268106

H 0.18650800 -2.42033600 -0.13839100<br>H -2.54606000 0.71949400 -1.63485300<br>H 3.45829500 1.72233400 0.04355600<br>H 2.04478000 -3.49326700 -0.96969800<br>H 1.96924100 -3.50940100 0.81767800<br>H 3.53252100 -3.71699700 -0.01341300<br>H -0.12536000 3.58478900 0.77812000<br>H -0.04685000 3.60556600 -1.00969500<br>H 1.08535700 4.59661300 -0.05288100<br>H -4.92011300 -0.01764600 -0.25131000<br>H -4.83968600 -0.11869900 -2.00873600<br>H -4.50495200 -1.55000600 -1.00838300<br>C -2.00160300 -0.88212700 -0.18055200<br>O -2.52859000 -0.99402600 1.17682600<br>H -2.02786600 -1.94394700 -0.46173800<br>O -2.63486100 0.29752500 1.75889900<br>H -3.55142200 0.55089200 1.56948800	• Thermal Correction to Free Energy<br>= 0.203315

Name	Anionic HPns RAF 1'-C···•OOH Product
Cartesian Coordinate:	Thermochemical Values:
O 2.89296700 -1.78858300 -0.03666300<br>O 1.50412300 2.69614800 -0.01968900<br>O 3.43592900 0.83645000 0.05310100<br>C -0.53260800 -0.38917500 -0.20420400<br>C 1.82412000 -0.91834800 -0.07474100<br>C 1.11283200 1.37460500 -0.06839200<br>C -0.21445900 0.97075000 -0.15539500<br>C 0.49972400 -1.33016000 -0.16125500<br>C 2.19645600 0.45334600 -0.02326100<br>C -2.91322300 -0.04790800 -1.01750600<br>C 2.60043900 -3.17630200 -0.08559100<br>C 0.48072700 3.67807600 -0.05982100<br>C -4.34834300 -0.43703700 -1.06611100<br>H -1.00821600 1.70646300 -0.17950300<br>H 0.25034900 -2.38398100 -0.19899700<br>H -2.56829100 0.83972300 -1.52975000<br>H 2.08840900 -3.43899500 -1.01573000<br>H 1.98644600 -3.48028100 0.76685800<br>H 3.55916300 -3.68976200 -0.04276200<br>H -0.19722100 3.57703700 0.79252600<br>H -0.09128100 3.61447100 -0.98989000<br>H 0.98382100 4.64194400 -0.00964500<br>H -4.88663300 -0.12221200 -0.16026800<br>H -4.85740700 0.01654700 -1.91767700<br>H -4.45836600 -1.52518500 -1.12785800<br>C -1.95621100 -0.87298700 -0.23548300<br>O -2.47516200 -1.11575500 1.11714500<br>H -1.97679600 -1.90949500 -0.60166000<br>O -2.58444700 0.11589400 1.81775800<br>H -3.50799000 0.37133100 1.67170500	• Electronic Energy<br>= -803.589661<br>• Zero Point Energy Coorection<br>= 0.236764<br>• Thermal Correction to Energy<br>= 0.254073<br>• Thermal Correction to Enthalpy<br>= 0.255018<br>• Thermal Correction to Free Energy<br>= 0.190623

Name	HPns RAF 2'-C···•OOH Product
Cartesian Coordinate:	Thermochemical Values:
O -3.05995800 -1.70475600 0.13511700<br>O -1.44818700 2.64411600 -0.06586800<br>O -3.43846400 0.90391800 0.18001200<br>C 0.44206600 -0.53952200 -0.30024000<br>C -1.94150200 -0.93856200 -0.00391400<br>C -1.08594900 1.32993800 -0.10939300<br>C 0.20430700 0.85914100 -0.26692800<br>C -0.65776200 -1.42720100 -0.16423700<br>C -2.16562900 0.44520200 0.02399600<br>C 1.74052900 -1.07299600 -0.46356000<br>C -2.88302600 -3.11829500 0.10745100<br>C -0.41132200 3.61413900 -0.19261000<br>C 4.20388900 -1.06484900 -0.92528200<br>H 1.02859400 1.55160300 -0.36518000<br>H -0.47100700 -2.49275200 -0.18824200<br>H 1.85185500 -2.15190200 -0.48405700<br>H -3.41814900 1.87197600 0.16782500<br>H -2.23630800 -3.44372400 0.92561900<br>H -2.46225500 -3.43725700 -0.84903600<br>H -3.87569100 -3.54457500 0.23212600<br>H 0.09491800 3.51168900 -1.15502200<br>H 0.30992500 3.51371700 0.62152500<br>H -0.90095300 4.58318400 -0.13428800<br>H 4.05966600 -1.54248600 -1.89655100<br>H 5.08729600 -0.42683700 -0.97779000<br>H 4.36807100 -1.84561000 -0.18040200<br>C 2.97743100 -0.24257900 -0.58032300<br>O 3.20306200 0.54974000 0.61534900	• Electronic Energy<br>= -804.080540<br>• Zero Point Energy Coorection<br>= 0.250622<br>• Thermal Correction to Energy<br>= 0.267711<br>• Thermal Correction to Enthalpy<br>= 0.268655<br>• Thermal Correction to Free Energy<br>= 0.205454

H 2.84065900 0.55616700 -1.31877300<br>O 3.33866100 -0.31222700 1.73649900<br>H 2.42426100 -0.41601900 2.04361100	

Name	Anionic HPns RAF 2'-C···•OOH Product
Cartesian Coordinate:	Thermochemical Values:
O -3.18532900 -1.56876900 0.15315000<br>O -1.31054000 2.71970100 -0.19653600<br>O -3.42121600 1.09260800 0.03337000<br>C 0.40011100 -0.58946200 -0.16439400<br>C -2.02480200 -0.84436300 0.03079200<br>C -1.05620200 1.37050900 -0.15279800<br>C 0.20584400 0.82823500 -0.21685000<br>C -0.77253500 -1.40333700 -0.03325100<br>C -2.24522100 0.57465400 -0.02651800<br>C 1.65408700 -1.19907200 -0.23671000<br>C -3.06213500 -2.98163100 0.22476800<br>C -0.19514100 3.58872900 -0.32588400<br>C 3.92689900 -1.29412300 -1.28027000<br>H 1.06730200 1.47626000 -0.29643600<br>H -0.64152600 -2.47753000 0.00953500<br>H 1.69729400 -2.28325800 -0.16417000<br>H -2.47050300 -3.27987800 1.09457200<br>H -2.60434100 -3.38240600 -0.68365400<br>H -4.07449100 -3.36849400 0.32314500<br>H 0.35010500 3.39175600 -1.25299300<br>H 0.48304800 3.48704100 0.52592300<br>H -0.60026200 4.59843300 -0.34934800<br>H 3.51279700 -1.40464100 -2.28347300<br>H 4.88922800 -0.78440900 -1.35440700<br>H 4.08138500 -2.28998500 -0.85797700<br>C 2.95428800 -0.50289100 -0.42084200<br>O 3.68413600 -0.33813900 0.83587200<br>H 2.82442700 0.50247300 -0.83208500<br>O 2.93217000 0.48787900 1.71091600<br>H 3.18060200 1.38491000 1.43893400	• Electronic Energy<br>= -803.613820<br>• Zero Point Energy Coorection<br>= 0.238495<br>• Thermal Correction to Energy<br>= 0.255075<br>• Thermal Correction to Enthalpy<br>= 0.256019<br>• Thermal Correction to Free Energy<br>= 0.193864

Name	HPs (4-propylsyringol)
Cartesian Coordinate:	Thermochemical Values:
O -2.07370400 -1.99362800 0.17122200<br>O -1.24964700 2.57750200 0.08729400<br>O -2.87716100 0.53157400 0.47834200<br>C 2.50460500 -0.48779300 -1.00770400<br>C 1.06304500 -0.21514600 -0.65064600<br>C 3.32243200 -0.99891500 0.18784800<br>C 0.61003900 1.09282700 -0.48292700<br>C 0.19100500 -1.28482500 -0.43740700<br>C -1.12557300 -1.03744500 -0.06027200<br>C -0.71100200 1.33750200 -0.10298600<br>C -1.58161200 0.27078300 0.10917300<br>C 3.37512400 -0.00064900 1.34013300<br>C -1.67403600 -3.35535500 0.04151000<br>C -0.38684700 3.69534800 -0.09923200<br>H 2.54566000 -1.22868000 -1.81118500<br>H 2.96091700 0.43149600 -1.38622900<br>H 2.89525400 -1.94510100 0.53566500<br>H 4.33721100 -1.21823000 -0.15648700<br>H 1.29494300 1.91518900 -0.64574600<br>H 0.54450100 -2.30026300 -0.56814300<br>H 3.76991000 0.96208300 1.00138800<br>H 2.38064800 0.17570700 1.75829400<br>H 4.01712000 -0.36337800 2.14591600<br>H -3.33549400 -0.31303300 0.58973800<br>H -0.87221300 -3.58990500 0.74519800<br>H -1.34788400 -3.56538400 -0.97961700<br>H -2.55473300 -3.94879500 0.27550000<br>H -0.01635100 3.73342000 -1.12624900<br>H 0.45371100 3.65755100 0.59779700<br>H -0.99218200 4.57574600 0.10434800	• Electronic Energy<br>= -654.371190<br>• Zero Point Energy Coorection<br>= 0.255725<br>• Thermal Correction to Energy<br>= 0.270721<br>• Thermal Correction to Enthalpy<br>= 0.271665<br>• Thermal Correction to Free Energy<br>= 0.213305

Name	HPs 1-OH radical
Cartesian Coordinate:	Thermochemical Values:

O -1.94283900 -2.14075900 0.19222100<br>O -1.41223200 2.50748500 0.10671400<br>O -2.87434600 0.32666900 0.49245700<br>C 2.46474600 -0.32571600 -1.02240800<br>C 1.02208000 -0.13628800 -0.65066700<br>C 3.30474700 -0.81876800 0.16961600<br>C 0.50294200 1.15722600 -0.48143000<br>C 0.22479300 -1.27619600 -0.42719300<br>C -1.08812100 -1.13468800 -0.04209300<br>C -0.80955900 1.32794500 -0.09141500<br>C -1.68212400 0.18459400 0.14693900<br>C 3.29886500 0.15921500 1.33986000<br>C -1.45408500 -3.47354200 0.03350900<br>C -0.63575200 3.69020600 -0.09232200<br>H 2.53148600 -1.05846800 -1.83175700<br>H 2.87424800 0.61949000 -1.38688600<br>H 2.92874700 -1.79353900 0.49485600<br>H 4.32832600 -0.97366100 -0.18130500<br>H 1.14876600 2.00764700 -0.65813900<br>H 0.66300800 -2.25625700 -0.56796500<br>H 3.63827100 1.14896300 1.02031900<br>H 2.29585800 0.26958800 1.76085700<br>H 3.96039900 -0.18305100 2.13845700<br>H -0.63072400 -3.66161900 0.72548900<br>H -1.12635700 -3.63837500 -0.99494500<br>H -2.29214300 -4.12522800 0.26624900<br>H -0.28768000 3.74681500 -1.12550700<br>H 0.21446100 3.70546700 0.59263000<br>H -1.30251100 4.52114100 0.12213600	• Electronic Energy = -653.739532<br>• Zero Point Energy Coorection = 0.243961<br>• Thermal Correction to Energy = 0.258349<br>• Thermal Correction to Enthalpy = 0.259293<br>• Thermal Correction to Free Energy = 0.201722

Name	HPs 1’-CH radical
Cartesian Coordinate:	Thermochemical Values:
O 2.93105400 -0.94313000 0.11945800<br>O 0.06439800 2.70863900 -0.00679300<br>O 2.48438700 1.68149800 0.18952700<br>C -1.85748900 -1.79459800 -0.39427300<br>C -0.76829900 -0.90525600 -0.24772400<br>C -3.28774600 -1.36993600 -0.47424900<br>C -0.95145900 0.50236700 -0.20674100<br>C 0.55001100 -1.42365000 -0.13721500<br>C 1.62321400 -0.56558300 0.00516800<br>C 0.13693800 1.34717600 -0.06177100<br>C 1.43115700 0.82272600 0.04502500<br>C -3.88408300 -1.06844600 0.91135100<br>C 3.21202900 -2.33983900 0.09486100<br>C -1.22952600 3.29531100 -0.10738100<br>H -1.63470600 -2.85670100 -0.38842700<br>H -3.38923000 -0.48133600 -1.10562800<br>H -3.87140000 -2.16288400 -0.94720400<br>H -1.94794700 0.91412300 -0.28454600<br>H 0.69635300 -2.49564500 -0.16781800<br>H -3.83215400 -1.95145000 1.55253500<br>H -3.33498000 -0.26107900 1.40175400<br>H -4.93123300 -0.76894100 0.82530800<br>H 3.29659600 1.15932200 0.25472300<br>H 2.89498000 -2.77951700 -0.85347500<br>H 2.71579100 -2.84658800 0.92553100<br>H 4.29058700 -2.42977900 0.20029400<br>H -1.87047500 2.96495000 0.71362900<br>H -1.69484400 3.04803000 -1.06449000<br>H -1.07452800 4.36980100 -0.04201800	• Electronic Energy = -653.722812<br>• Zero Point Energy Coorection = 0.242068<br>• Thermal Correction to Energy = 0.257001<br>• Thermal Correction to Enthalpy = 0.257945<br>• Thermal Correction to Free Energy = 0.199714

Name	HPs 1’-CH radical anion
Cartesian Coordinate:	Thermochemical Values:

O 0.01566100 -2.73090500 -0.00468600<br>O -3.00310800 0.86105000 0.13152100<br>O -2.47301200 -1.76224300 0.18637100<br>C 1.75447200 1.84129000 -0.38729600<br>C 0.70901000 0.92374100 -0.23925100<br>C 3.20292600 1.46571600 -0.47865300<br>C -0.64362200 1.37779100 -0.12404800<br>C 0.92787000 -0.48865700 -0.19786500<br>C -0.12821800 -1.36304800 -0.05608700<br>C -1.68290100 0.48815900 0.01585700<br>C -1.48960600 -0.93262300 0.05662100<br>C 3.83256600 1.16147500 0.89090500<br>C 1.33250800 -3.25061900 -0.10511200<br>C -3.28765300 2.25100300 0.09173400<br>H 1.49985500 2.89731900 -0.38436100<br>H 3.33280800 0.59110400 -1.12612100<br>H 3.75830400 2.28253500 -0.94701200<br>H -0.82622300 2.44515700 -0.15247700<br>H 1.93907000 -0.86554500 -0.27472300<br>H 3.76525900 2.03316100 1.54662700<br>H 3.31366200 0.33265900 1.37919200<br>H 4.88722200 0.89179700 0.78903300<br>H 1.95995000 -2.89451200 0.71684600<br>H 1.79188100 -2.97811900 -1.05929200<br>H 1.23559300 -4.33299300 -0.04501400<br>H -2.97314400 2.68975600 -0.85928200<br>H -2.79736000 2.77676900 0.91566500<br>H -4.36730700 2.34233300 0.19445200	• Electronic Energy<br>= -653.252928<br>• Zero Point Energy Coorection<br>= 0.229688<br>• Thermal Correction to Energy<br>= 0.244053<br>• Thermal Correction to Enthalpy<br>= 0.244998<br>• Thermal Correction to Free Energy<br>= 0.187821

Name	HPs HT 1-OH···•OOH Transition State
Cartesian Coordinate:	Thermochemical Values:
O -0.65569800 2.65591000 -0.48014200<br>O -1.49603600 -1.91893700 -0.67891600<br>C 2.81000000 -0.41012000 1.23076800<br>C 1.45275200 -0.15602500 0.63011500<br>C 3.94358900 -0.18107900 0.21715100<br>C 0.62412900 -1.22483300 0.28785500<br>C 1.05752300 1.16672600 0.38018900<br>C -0.17052300 1.42579100 -0.20719500<br>C -0.60305600 -0.97494600 -0.31732700<br>C 3.85469400 -1.10920000 -0.98993000<br>C 0.16585800 3.77402300 -0.14692300<br>C -1.19200000 -3.27611600 -0.35154600<br>H 2.95445700 0.25738300 2.08477600<br>H 2.85705300 -1.43883000 1.59751700<br>H 3.92443700 0.86249700 -0.11211700<br>H 4.89569100 -0.33375900 0.73251100<br>H 0.94681300 -2.23683700 0.49404700<br>H 1.71971200 1.97549400 0.66195700<br>H 3.83722700 -2.15641900 -0.67359400<br>H 2.94803500 -0.92079500 -1.57089800<br>H 4.71107900 -0.97147400 -1.65348400<br>H 1.10884600 3.73405800 -0.69634200<br>H 0.35888600 3.80265900 0.92750900<br>H -0.39846800 4.65426300 -0.44447800<br>H -1.06813100 -3.38875900 0.72786400<br>H -0.28851900 -3.60244900 -0.87024700<br>H -2.04464000 -3.85884000 -0.69061900<br>C -1.02035400 0.35347800 -0.57895400<br>O -2.18165200 0.59360700 -1.18777600<br>H -2.97011100 0.27796000 -0.56397000<br>O -3.72005600 -0.05446600 0.56427000<br>O -2.76000600 -0.24979800 1.49427300<br>H -2.52045300 -1.19288000 1.43069000	• Electronic Energy<br>= -805.265808<br>• Zero Point Energy Coorection<br>= 0.268213<br>• Thermal Correction to Energy<br>= 0.285804<br>• Thermal Correction to Enthalpy<br>= 0.286749<br>• Thermal Correction to Free Energy<br>= 0.221748

Name	HPs HT 1'-CH···•OOH Transition State
Cartesian Coordinate:	Thermochemical Values:
O -1.03612100 2.73953500 0.12215200<br>O -2.84361800 -1.51699600 -0.28977700<br>O -3.11946400 1.08770800 -0.01353100<br>C 2.05945900 -1.09251700 -0.31497300<br>C 0.71588900 -0.49380800 -0.27627500<br>C 3.20731400 -0.30276600 -0.91568300<br>C -0.40942100 -1.33173300 -0.32279500<br>C 0.54553500 0.89146700 -0.11808400<br>C -0.73463900 1.41845300 -0.02605500<br>C -1.68908200 -0.79635100 -0.23703100<br>C -1.85599600 0.58351100 -0.08850500<br>C 3.01515900 -0.07281400 -2.41702600<br>C 0.05336100 3.65431800 0.21685600<br>C -2.72120600 -2.92805300 -0.44811100	• Electronic Energy<br>= -805.255144<br>• Zero Point Energy Coorection<br>= 0.267254<br>• Thermal Correction to Energy<br>= 0.285369<br>• Thermal Correction to Enthalpy<br>= 0.286313

H 2.03696300 -2.13315300 -0.64558200<br>H 3.31884800 0.65683600 -0.40388600<br>H 4.13350900 -0.85709300 -0.74537600<br>H -0.26511600 -2.39848100 -0.43564900<br>H 1.40758700 1.54337600 -0.07025900<br>H 2.90635800 -1.02507900 -2.94254500<br>H 2.11951000 0.52324400 -2.60858000<br>H 3.87260800 0.45390700 -2.84144900<br>H -3.06462500 2.05096800 0.06538900<br>H 0.65518200 3.63119500 -0.69433300<br>H 0.67608500 3.42006300 1.08318800<br>H -0.39337600 4.63801500 0.33905500<br>H -2.18282000 -3.36753400 0.39478100<br>H -2.21054800 -3.16987300 -1.38318900<br>H -3.73787100 -3.31303600 -0.47473100<br>H 2.34671500 -1.19174500 0.89503800<br>O 1.24904500 -0.82219200 2.67359500<br>O 2.51477800 -1.08549300 2.20300800<br>H 1.14578100 0.13964800 2.59064600	• Thermal Correction to Free Energy<br>= 0.219762

Name	Anionic HPs HT 1’-CH···•OOH Transition State
Cartesian Coordinate:	Thermochemical Values:
O -0.77820900 2.84185100 0.19905800<br>O -2.97983700 -1.25884400 -0.35917500<br>O -2.99264300 1.37647200 0.02664200<br>C 1.91773500 -1.22844400 -0.34968800<br>C 0.65530200 -0.52991000 -0.33313400<br>C 3.16557900 -0.51250300 -0.85133000<br>C -0.56114000 -1.26575400 -0.40734500<br>C 0.59094400 0.87546500 -0.12652900<br>C -0.62098000 1.50906100 -0.00055700<br>C -1.77324900 -0.63800900 -0.28977000<br>C -1.87809400 0.79196000 -0.07854000<br>C 3.09437900 -0.15514300 -2.33740300<br>C 0.40198500 3.63226700 0.29857400<br>C -2.97515100 -2.66827100 -0.55870600<br>H 1.82417800 -2.24487900 -0.73903700<br>H 3.34722000 0.39286800 -0.26399400<br>H 4.02745300 -1.16230500 -0.67445900<br>H -0.50067300 -2.33607800 -0.55998500<br>H 1.51118400 1.44059000 -0.06517000<br>H 2.93839900 -1.05307400 -2.94163900<br>H 2.26314200 0.52802400 -2.53273200<br>H 4.01531100 0.32584600 -2.67688800<br>H 0.98357100 3.57688700 -0.62489000<br>H 1.01467400 3.30711000 1.14297500<br>H 0.06488400 4.65353200 0.46003300<br>H -2.46390100 -3.17375400 0.26415300<br>H -2.49361100 -2.92332400 -1.50597700<br>H -4.01948900 -2.97066600 -0.58415800<br>H 2.14347500 -1.35785600 0.91508600<br>O 0.97625600 -1.11713000 2.66803500<br>O 2.33475700 -1.29803900 2.24296800<br>H 0.75772700 -0.22171000 2.37303000	• Electronic Energy<br>= -804.796644<br>• Zero Point Energy Coorection<br>= 0.254488<br>• Thermal Correction to Energy<br>= 0.272390<br>• Thermal Correction to Enthalpy<br>= 0.273334<br>• Thermal Correction to Free Energy<br>= 0.207021

Name	HPs HT 1-OH···•OOH Reactant Complex
Cartesian Coordinate:	Thermochemical Values:
O 0.18370000 2.88390300 -0.27604500<br>O -1.84149300 -1.32762600 -0.34173300<br>C 2.88015300 -1.01369700 1.16029500<br>C 1.58554300 -0.39932100 0.68536400<br>C 3.95603500 -1.04069800 0.06453800<br>C 0.47274700 -1.19337900 0.42689800<br>C 1.51791000 0.97789400 0.45724100<br>C 0.34631900 1.55368300 -0.02284200<br>C -0.69841200 -0.61332900 -0.05943200<br>C 3.54048700 -1.86204200 -1.15187400<br>C 1.30337200 3.73214400 -0.04107400<br>C -1.70959200 -2.74710800 -0.49596200<br>H 3.25663600 -0.44763500 2.01713200<br>H 2.68935300 -2.03549000 1.50093700<br>H 4.18264200 -0.01391100 -0.24043300<br>H 4.87443100 -1.45288500 0.49225400<br>H 0.52410000 -2.26129800 0.59766400<br>H 2.38651900 1.59168700 0.66094100<br>H 3.28113000 -2.88412300 -0.85948100<br>H 2.66864100 -1.42535600 -1.64618600<br>H 4.34833500 -1.91598900 -1.88496100<br>H 2.14772500 3.44446200 -0.67198700<br>H 1.59859700 3.70404200 1.01041000<br>H 0.97588000 4.73577400 -0.30260000	• Electronic Energy<br>= -805.285513<br>• Zero Point Energy Coorection<br>= 0.273287<br>• Thermal Correction to Energy<br>= 0.291699<br>• Thermal Correction to Enthalpy<br>= 0.292643<br>• Thermal Correction to Free Energy<br>= 0.224418

H -1.48574400 -3.22029300 0.46128800<br>H -0.92873700 -2.97154500 -1.22394900<br>H -2.67364900 -3.09341400 -0.86224300<br>C -0.77619200 0.75805800 -0.28864500<br>O -1.89081100 1.37199300 -0.79226800<br>H -2.68003800 0.81831600 -0.66635700<br>O -4.36237300 0.28232300 0.11997700<br>O -4.14280900 -0.75952800 0.86733500<br>H -3.24575700 -1.09408600 0.59241500	

Name	HPs HT 1’-CH⋯•OOH Reactant Complex
Cartesian Coordinate:	Thermochemical Values:
O 2.16411100 -1.48134600 -1.13007500<br>O 0.73008200 2.85136100 -0.27459300<br>O 2.56426500 1.15212300 -1.13220800<br>C -2.40435700 -0.88001800 0.70796400<br>C -1.07219000 -0.34034100 0.24533500<br>C -3.28875100 -1.33739000 -0.46137600<br>C -0.82454900 1.03498500 0.24194900<br>C -0.09251500 -1.22281300 -0.21819500<br>C 1.12422400 -0.72548500 -0.67509600<br>C 0.39507200 1.53148600 -0.22837200<br>C 1.37115800 0.64949400 -0.68733300<br>C -3.62623800 -0.20399200 -1.42485400<br>C 2.00364600 -2.89788700 -1.10547800<br>C -0.23627100 3.78435000 0.20154300<br>H -2.92887600 -0.10477400 1.27382300<br>H -2.78096500 -2.14429500 -0.99951500<br>H -4.21019000 -1.76099500 -0.05196900<br>H -1.59048400 1.71101200 0.59976900<br>H -0.28291800 -2.28886600 -0.20445000<br>H -4.10998400 0.62299900 -0.89632900<br>H -2.72683200 0.18874400 -1.90638100<br>H -4.30434100 -0.54448100 -2.21043600<br>H 3.11696600 0.41142700 -1.41908200<br>H 1.17514600 -3.20248500 -1.74863800<br>H 1.83263900 -3.24620400 -0.08419400<br>H 2.93547300 -3.31010300 -1.48500200<br>H -0.46041100 3.60585800 1.25587300<br>H -1.15336600 3.72865500 -0.38936800<br>H 0.21630200 4.76622800 0.08413000<br>H -2.23647900 -1.72363100 1.38380900<br>O 0.82982400 -0.18124300 2.78912000<br>O 0.86675800 -1.47837700 2.88939100<br>H 0.17262800 0.03643600 2.08836700	• Electronic Energy<br>= -805.282894<br>• Zero Point Energy Coorection<br>= 0.272624<br>• Thermal Correction to Energy<br>= 0.291532<br>• Thermal Correction to Enthalpy<br>= 0.292476<br>• Thermal Correction to Free Energy<br>= 0.222802

Name	Anionic HPs HT 1’-CH⋯•OOH Reactant Complex
Cartesian Coordinate:	Thermochemical Values:
O 0.44454700 2.54307100 -1.20684600<br>O -3.10612000 -0.15655900 0.20487000<br>O -2.13971900 2.10752200 -0.78443400<br>C 1.47602000 -1.97362000 0.44444700<br>C 0.49173700 -0.88681700 0.12011400<br>C 2.12061700 -2.57088700 -0.81763600<br>C -0.88009800 -1.08539000 0.34152800<br>C 0.97557600 0.32458300 -0.41656300<br>C 0.10138000 1.34342700 -0.71798200<br>C -1.77993400 -0.08337200 0.03888300<br>C -1.33538100 1.19238000 -0.50965800<br>C 1.09610100 -3.20836200 -1.75041700<br>C 1.83645200 2.80022200 -1.40236200<br>C -3.64423400 -1.36792000 0.73853300<br>H 0.97233500 -2.76470700 1.00524900<br>H 2.67218200 -1.78767800 -1.34627600<br>H 2.85386000 -3.31750500 -0.50196500<br>H -1.21229600 -2.02898700 0.75463900<br>H 2.04149100 0.44040000 -0.56586500<br>H 0.51784200 -3.97448400 -1.22558300<br>H 0.39336400 -2.46388200 -2.13488800<br>H 1.58405700 -3.68034300 -2.60580100<br>H 2.25230500 2.10663000 -2.13596400<br>H 2.37346100 2.71308600 -0.45539700<br>H 1.89947300 3.81899300 -1.77552900<br>H -3.24750500 -1.55102600 1.73900300<br>H -3.41249900 -2.20837100 0.08137500<br>H -4.71943900 -1.21716300 0.78578800<br>H 2.25865500 -1.55215000 1.08385700<br>O 1.03861000 0.75205700 2.86888200<br>O 2.44246500 0.96930100 2.52540000	• Electronic Energy<br>= -804.816427<br>• Zero Point Energy Coorection<br>= 0.259324<br>• Thermal Correction to Energy<br>= 0.278135<br>• Thermal Correction to Enthalpy<br>= 0.279079<br>• Thermal Correction to Free Energy<br>= 0.209355

H 0.58504800 0.83860000 2.02105600	

Name	HPs HT 1-OH···•OOH Product Complex
Cartesian Coordinate:	Thermochemical Values:
O -0.12720400 2.85382900 0.22741600<br>O 1.76982400 -1.42301500 0.24501700<br>C -2.95598200 -0.94999400 -1.07981900<br>C -1.63411100 -0.37795600 -0.65715700<br>C -3.97480200 -0.94730200 0.07454400<br>C -0.53818900 -1.22960700 -0.43505200<br>C -1.53330500 1.00950300 -0.44173500<br>C -0.34679500 1.55492900 -0.00818800<br>C 0.66161600 -0.70973200 -0.00199600<br>C -3.51648000 -1.77960000 1.26751000<br>C -1.21185400 3.75864800 0.00930100<br>C 1.69660300 -2.84563500 0.09874500<br>H -3.35349100 -0.35492800 -1.90685600<br>H -2.81430500 -1.97353700 -1.43492700<br>H -4.15967100 0.08486000 0.38698700<br>H -4.92012200 -1.33731300 -0.31137100<br>H -0.65706200 -2.29140900 -0.60744800<br>H -2.40001200 1.63148500 -0.62612000<br>H -3.28892000 -2.80495100 0.96141400<br>H -2.61612100 -1.35913900 1.72366100<br>H -4.29162400 -1.82067700 2.03556800<br>H -2.04988100 3.51128100 0.66386900<br>H -1.52719200 3.72737500 -1.03540300<br>H -0.82662800 4.74507500 0.25319300<br>H 1.46108700 -3.10847000 -0.93413700<br>H 0.94508100 -3.25470200 0.77608500<br>H 2.68310000 -3.21735700 0.36427400<br>C 0.82135900 0.71729400 0.23800900<br>O 1.90035500 1.21242900 0.63762600<br>H 3.49980500 0.47886100 0.70458400<br>O 4.33793600 0.02127000 0.47467400<br>O 4.13203700 -0.28148200 -0.90104300<br>H 3.40401200 -0.92399600 -0.86785500	• Electronic Energy = -805.302955<br>• Zero Point Energy Coorection = 0.273244<br>• Thermal Correction to Energy = 0.291808<br>• Thermal Correction to Enthalpy = 0.292752<br>• Thermal Correction to Free Energy = 0.223494

Name	HPs HT 1'-CH···•OOH Product Complex
Cartesian Coordinate:	Thermochemical Values:
O -0.30800900 2.73291000 -0.33766000<br>O -3.02097800 -1.03631800 -0.40709400<br>O -2.70790400 1.58315600 -0.38054400<br>C 1.81860200 -1.69682700 -0.29307100<br>C 0.69225500 -0.84343000 -0.34849900<br>C 3.23623000 -1.22180300 -0.29353200<br>C -0.61276500 -1.40486300 -0.37920800<br>C 0.82563500 0.57120400 -0.33759500<br>C -0.30557400 1.36881600 -0.34776500<br>C -1.73228000 -0.59226200 -0.38720900<br>C -1.58904700 0.80295300 -0.37149400<br>C 3.72461100 -0.85320700 -1.70496400<br>C 0.95509900 3.38841900 -0.25336200<br>C -3.21900500 -2.44709000 -0.40190900<br>H 1.63223800 -2.76544500 -0.32825300<br>H 3.34931600 -0.35075600 0.36093900<br>H 3.87696600 -2.00807500 0.11149700<br>H -0.71308500 -2.48245700 -0.38229700<br>H 1.80771500 1.02286200 -0.31587900<br>H 3.65771000 -1.71556500 -2.37218200<br>H 3.11649000 -0.04966500 -2.12710200<br>H 4.76455500 -0.51984400 -1.67865100<br>H -2.43321400 2.51103400 -0.35437900<br>H 1.57007900 3.15233500 -1.12455500<br>H 1.47624900 3.09935600 0.66238700<br>H 0.73756400 4.45359600 -0.23404200<br>H -2.79304500 -2.89428200 0.49932700<br>H -2.77466200 -2.90419200 -1.28912500<br>H -4.29614000 -2.59660300 -0.41246700<br>H 1.57249400 -0.97737000 2.09170000<br>O -0.00979900 -0.20138700 2.78481100<br>O 1.40554300 -0.31142100 2.77939600<br>H -0.18460500 0.45809500 2.09345200	• Electronic Energy = -805.284001<br>• Zero Point Energy Coorection = 0.271559<br>• Thermal Correction to Energy = 0.290696<br>• Thermal Correction to Enthalpy = 0.291640<br>• Thermal Correction to Free Energy = 0.223492

Name	Anionic HPs HT 1'-CH···•OOH Product

	Complex
Cartesian Coordinate:	Thermochemical Values:
O -0.23187700 2.78744100 -0.28200800<br>O -3.11137100 -0.91772700 -0.36046400<br>O -2.68527100 1.72577000 -0.30800200<br>C 1.70650700 -1.71621500 -0.30312200<br>C 0.61707800 -0.83962000 -0.35051600<br>C 3.14328800 -1.28254100 -0.34558800<br>C -0.72313700 -1.34503100 -0.36939700<br>C 0.77942100 0.58256400 -0.32923600<br>C -0.31776100 1.41781300 -0.30795000<br>C -1.80353400 -0.49684500 -0.35056100<br>C -1.66923300 0.93416700 -0.31987500<br>C 3.61416400 -0.91280000 -1.76169100<br>C 1.06781700 3.35653300 -0.22383100<br>C -3.33892300 -2.31899800 -0.38674500<br>H 1.49414800 -2.78091400 -0.34302700<br>H 3.30753200 -0.42206300 0.31397200<br>H 3.77296100 -2.08979700 0.03723300<br>H -0.86016000 -2.41947100 -0.38224300<br>H 1.77858600 0.99714400 -0.32111600<br>H 3.50950300 -1.76619500 -2.43613800<br>H 3.01768800 -0.09011300 -2.16405400<br>H 4.66327700 -0.60560000 -1.75948200<br>H 1.65232000 3.09389300 -1.10973500<br>H 1.59801600 3.02938900 0.67510200<br>H 0.92367400 4.43462200 -0.19024400<br>H -2.91570900 -2.80167000 0.49844900<br>H -2.91206900 -2.76906800 -1.28721700<br>H -4.41920100 -2.45021600 -0.39141200<br>H 1.60972600 -1.09367000 1.87410700<br>O 0.14986600 -0.23293900 2.72471600<br>O 1.54529200 -0.48267800 2.63271900<br>H -0.00751900 0.42062400 2.02120100	• Electronic Energy<br>= -804.816382<br>• Zero Point Energy Coorection<br>= 0.258983<br>• Thermal Correction to Energy<br>= 0.277550<br>• Thermal Correction to Enthalpy<br>= 0.278494<br>• Thermal Correction to Free Energy<br>= 0.211202

Name	HPs RAF 1-C⋯•OOH Product
Cartesian Coordinate:	Thermochemical Values:
O 1.39466600 -2.29194100 -0.44558800<br>O 1.22105900 2.39258700 -0.40362900<br>O 1.90805600 0.10914700 -1.67296200<br>C -2.75670800 -0.12909600 1.15704800<br>C -1.34274200 -0.05749100 0.65654000<br>C -3.74663800 -0.57711500 0.06715100<br>C -0.72823900 1.18866900 0.39400300<br>C -0.64224200 -1.25919100 0.37068400<br>C 0.62491700 -1.22730200 -0.12259800<br>C 0.53765200 1.26157500 -0.11440100<br>C -3.77828800 0.37411600 -1.12475200<br>C 0.81531700 -3.58612300 -0.29434300<br>C 0.55075200 3.63133400 -0.18387400<br>H -2.81163400 -0.83304200 1.99380700<br>H -3.05898200 0.85295800 1.53191300<br>H -3.47920900 -1.58443000 -0.26767600<br>H -4.74230000 -0.64613100 0.51448400<br>H -1.28792200 2.09465800 0.59314900<br>H -1.13723300 -2.20695100 0.54850600<br>H -4.01329500 1.39289800 -0.80201500<br>H -2.81220000 0.40067900 -1.63609400<br>H -4.53336100 0.06842200 -1.85231400<br>H 2.28934700 -0.75664800 -1.88041400<br>H -0.07513100 -3.67926900 -0.92026400<br>H 0.55788300 -3.76699300 0.75164600<br>H 1.57382700 -4.29436700 -0.61794000<br>H 0.29453700 3.74496600 0.87208800<br>H -0.35290500 3.68986200 -0.79499000<br>H 1.25088800 4.40794200 -0.48147300<br>C 1.37315300 0.04744800 -0.37865400<br>O 2.58788600 0.08513400 0.44062400<br>O 2.23134400 -0.01887900 1.80633200<br>H 2.15200600 0.90612200 2.08854300	• Electronic Energy<br>= -805.280172<br>• Zero Point Energy Coorection<br>= 0.273694<br>• Thermal Correction to Energy<br>= 0.291474<br>• Thermal Correction to Enthalpy<br>= 0.292418<br>• Thermal Correction to Free Energy<br>= 0.226962

Name	Anionic HPs RAF 1-C⋯•OOH Product
Cartesian Coordinate:	Thermochemical Values:
O 1.36853200 -2.34746700 -0.45765300<br>O 1.25684200 2.38512400 -0.40116500<br>O 1.86086000 0.04444000 -1.76467600<br>C -2.72953700 -0.11414800 1.16095700<br>C -1.31481300 -0.05522000 0.65802400<br>C -3.73229300 -0.52552100 0.06791300	• Electronic Energy<br>= -804.805077<br>• Zero Point Energy Coorection

C -0.68632300 1.18687000 0.39962000<br>C -0.62740300 -1.26339000 0.36261900<br>C 0.63078700 -1.24675400 -0.16211000<br>C 0.57164700 1.24238900 -0.13292300<br>C -3.74979500 0.44399400 -1.10963400<br>C 0.77485600 -3.61747700 -0.21908200<br>C 0.61277800 3.61860300 -0.10714100<br>H -2.79787300 -0.83158800 1.98580400<br>H -3.01686700 0.86565500 1.55467500<br>H -3.48411500 -1.53194600 -0.28445800<br>H -4.72898000 -0.58398600 0.51538600<br>H -1.23315500 2.09604600 0.62251300<br>H -1.12982900 -2.20321800 0.56359600<br>H -3.96395400 1.46225000 -0.77073800<br>H -2.78466800 0.45992600 -1.62302800<br>H -4.51277500 0.16455100 -1.83969500<br>H -0.13186400 -3.73814000 -0.81747100<br>H 0.53629800 -3.74075700 0.84044000<br>H 1.51370800 -4.35771600 -0.51797400<br>H 0.38106000 3.68954800 0.95882300<br>H -0.30506900 3.72414100 -0.69116000<br>H 1.31614200 4.40106800 -0.38315200<br>C 1.38114300 0.02085000 -0.52500900<br>O 2.63379400 0.04319500 0.34270200<br>O 2.30217500 0.00521200 1.72730500<br>H 2.17803200 0.93895000 1.95492700	= 0.260326<br>• Thermal Correction to Energy = 0.277625<br>• Thermal Correction to Enthalpy = 0.278569<br>• Thermal Correction to Free Energy = 0.214360

Name	HPs RAF 2-C···•OOH Product
Cartesian Coordinate:	Thermochemical Values:
O 1.82762600 -1.17377700 -1.24240500<br>O -0.00891000 2.88869100 -0.37760900<br>O 2.19847500 1.52498800 -0.84135100<br>C -2.33775400 -1.23322800 1.15186000<br>C -1.08857900 -0.53697300 0.66538600<br>C -3.14858400 -1.85301300 0.00328000<br>C -1.15063100 0.87570900 0.40463800<br>C 0.04722900 -1.23796400 0.44388500<br>C -0.04562500 1.55130000 -0.09703400<br>C 1.12970400 0.86043100 -0.35490800<br>C -3.62450500 -0.83097600 -1.02494900<br>C 1.86790800 -2.60492200 -1.28848100<br>C -1.20225800 3.63011200 -0.14023700<br>H -2.05900300 -2.02035400 1.85636600<br>H -2.96517100 -0.51515300 1.68833700<br>H -2.53511500 -2.61499600 -0.48843100<br>H -4.01157700 -2.36892500 0.43376100<br>H -2.07551300 1.39924700 0.60550800<br>H 0.10291700 -2.29634700 0.67460900<br>H -4.20903900 -0.03984600 -0.54578700<br>H -2.78295400 -0.35981500 -1.53962600<br>H -4.25471200 -1.30405900 -1.78129100<br>H 2.91937300 0.90180300 -1.01754700<br>H 0.86841900 -3.01196600 -1.45143500<br>H 2.29726500 -3.01782400 -0.37450900<br>H 2.50450000 -2.85611300 -2.13442500<br>H -1.47833000 3.59383300 0.91626500<br>H -2.02372200 3.24996100 -0.75245500<br>H -0.97673600 4.65528300 -0.42484000<br>C 1.28627500 -0.60108500 -0.07717100<br>O 2.40601400 -0.78102000 0.86164900<br>O 2.05803400 -0.23078600 2.11750400<br>H 2.43039500 0.66415500 2.07558100	• Electronic Energy = -805.268438<br>• Zero Point Energy Coorection = 0.273152<br>• Thermal Correction to Energy = 0.291057<br>• Thermal Correction to Enthalpy = 0.292001<br>• Thermal Correction to Free Energy = 0.226220

Name	Anionic HPs RAF 2-C···•OOH Product
Cartesian Coordinate:	Thermochemical Values:
O 1.42907600 -1.46206700 -1.30712700<br>O 0.56748600 2.88677300 -0.44322200<br>O 2.51181800 1.06648200 -0.74135100<br>C -2.48576500 -0.64467500 1.26142900<br>C -1.13984900 -0.21396800 0.72108500<br>C -3.34450500 -1.35641100 0.20471900<br>C -0.94628300 1.16296900 0.37664500<br>C -0.15849800 -1.13509800 0.53135200<br>C 0.27447900 1.57944900 -0.12410500<br>C 1.35825500 0.68789100 -0.33961900<br>C -3.62678400 -0.48984300 -1.01887800<br>C 1.03881500 -2.83308100 -1.38109500<br>C -0.45829600 3.84627800 -0.23679200<br>H -2.33874900 -1.31850700 2.11015500<br>H -3.02705500 0.23220700 1.62968900<br>H -2.83537400 -2.27542000 -0.10385000	• Electronic Energy = -804.810697<br>• Zero Point Energy Coorection = 0.260807<br>• Thermal Correction to Energy = 0.278197<br>• Thermal Correction to Enthalpy = 0.279141<br>• Thermal Correction to Free Energy

H -4.28872000 -1.65706200 0.66861100<br>H -1.75912200 1.85891100 0.53476400<br>H -0.31003600 -2.16884500 0.82658700<br>H -4.10538700 0.45016500 -0.72760600<br>H -2.70372700 -0.24313000 -1.55038400<br>H -4.28985400 -1.00246000 -1.71951900<br>H -0.04787700 -2.92915600 -1.44193800<br>H 1.41060700 -3.40232500 -0.52634800<br>H 1.48530000 -3.22468900 -2.29385600<br>H -0.74625100 3.89327300 0.81692400<br>H -1.33753400 3.61962600 -0.84605800<br>H -0.04234300 4.80432400 -0.54286600<br>C 1.14435100 -0.79240300 -0.08653000<br>O 2.26686200 -1.29638100 0.73371100<br>O 2.25357100 -0.65192100 1.99372600<br>H 2.77010300 0.15335000 1.83051300	= 0.214536

Name	HPs RAF 3-C⋯•OOH Product
Cartesian Coordinate:	Thermochemical Values:
O 0.40802000 -2.45727000 -1.01911700<br>O 2.81364400 1.41427900 -0.03419300<br>O 2.72640900 -1.08549900 -0.84975100<br>C -2.09728500 1.55664600 0.65885700<br>C -0.81816300 0.85574100 0.29267200<br>C -3.23969800 1.33835000 -0.34375700<br>C 0.38422500 1.50809800 0.28534700<br>C 0.38045900 -1.21270300 -0.43753300<br>C 1.58717700 0.84416000 -0.03035900<br>C 1.55246900 -0.52469100 -0.42427000<br>C -2.85673100 1.74072100 -1.76438100<br>C -0.32515300 -3.50114200 -0.36763500<br>C 2.90788700 2.78622000 0.34713700<br>H -2.42601800 1.21186500 1.64692900<br>H -1.89098800 2.62719200 0.74645500<br>H -3.55624000 0.29089700 -0.32444500<br>H -4.09976000 1.92503400 -0.01050700<br>H 0.40617600 2.56502500 0.52323300<br>H -2.51301000 2.77906600 -1.79266200<br>H -2.04736000 1.11346300 -2.14958700<br>H -3.70555900 1.64682000 -2.44508900<br>H 2.54253600 -2.00612700 -1.08678700<br>H -1.39927500 -3.32218400 -0.43249000<br>H -0.02174300 -3.58357100 0.67899800<br>H -0.07497800 -4.41712000 -0.89961000<br>H 2.53741500 2.92811800 1.36448500<br>H 2.34972500 3.41741900 -0.34735600<br>H 3.96553500 3.03374300 0.30119400<br>C -0.89952600 -0.61882900 0.03526500<br>O -1.41427500 -1.30139100 1.23196900<br>O -0.52485100 -1.09066600 2.31664400<br>H 0.11361900 -1.81573300 2.22410100<br>H -1.70560700 -0.86496600 -0.66934100	• Electronic Energy = -805.268700<br>• Zero Point Energy Coorection = 0.274211<br>• Thermal Correction to Energy = 0.291802<br>• Thermal Correction to Enthalpy = 0.292747<br>• Thermal Correction to Free Energy = 0.228421

Name	Anionic HPs RAF 3-C⋯•OOH Product
Cartesian Coordinate:	Thermochemical Values:
O 1.16996600 -2.20470800 -1.08324500<br>O 2.03747400 2.34596500 0.02439800<br>O 2.90039200 0.00006000 -0.92060600<br>C -2.54470200 0.59681600 0.70234300<br>C -1.10956300 0.44497200 0.28270800<br>C -3.55633000 0.04270600 -0.31157800<br>C -0.22292300 1.49342000 0.32854900<br>C 0.77498000 -0.98101800 -0.53689500<br>C 1.12776100 1.33886300 -0.01868900<br>C 1.66708800 0.06813600 -0.51266200<br>C -3.40103800 0.66713900 -1.69469800<br>C 1.95632200 -3.00241600 -0.19755700<br>C 1.60216400 3.62211200 0.48049400<br>H -2.69698500 0.08584600 1.66187900<br>H -2.74719600 1.65862800 0.87240300<br>H -3.45232200 -1.04462200 -0.38022200<br>H -4.56311600 0.23247200 0.07046400<br>H -0.58962700 2.46408600 0.64330600<br>H -3.48052700 1.75700300 -1.63824700<br>H -2.42589000 0.42826600 -2.12910800<br>H -4.17080400 0.30749100 -2.38107500<br>H 1.35632400 -3.33526500 0.65521500<br>H 2.82518100 -2.44338100 0.15797800<br>H 2.29168800 -3.87386900 -0.76071600<br>H 1.22434900 3.55986200 1.50380200<br>H 0.82845100 4.02628100 -0.17651900	• Electronic Energy = -804.797121<br>• Zero Point Energy Coorection = 0.260977<br>• Thermal Correction to Energy = 0.278274<br>• Thermal Correction to Enthalpy = 0.279218<br>• Thermal Correction to Free Energy = 0.215435

H 2.47901600 4.26514200 0.45199700<br>C -0.63620100 -0.91949400 -0.09804500<br>O -0.89802300 -1.87516600 1.01029800<br>O -0.23907500 -1.43544200 2.18920900<br>H 0.70230500 -1.57827500 1.99507700<br>H -1.29206400 -1.38274600 -0.85046400	

Name	HPs RAF 4-C···•OOH Product
Cartesian Coordinate:	Thermochemical Values:
O 2.40193700 1.48895800 -0.42880800<br>O -2.23433900 1.77847000 -0.24341800<br>O 0.13940500 2.84401500 -0.62481900<br>C -0.16058800 -2.59291400 -0.00329300<br>C 0.15923400 -2.67350300 -1.49805700<br>C -1.26342400 -0.35296500 0.36726300<br>C 1.24463100 -0.52301300 0.27023600<br>C 1.28441800 0.77572300 -0.11590700<br>C -1.17919400 0.94509900 -0.01619400<br>C 0.09019400 1.54714200 -0.25231600<br>C -0.70724600 -1.79689600 -2.40006500<br>C 3.65185200 0.81340200 -0.31655700<br>C -3.53298200 1.22803100 -0.04668600<br>H 0.53577000 -3.24227800 0.53663200<br>H -1.17272500 -2.96372000 0.18613300<br>H 1.21315800 -2.42516900 -1.65542100<br>H 0.04187200 -3.71991800 -1.79356200<br>H -2.21865100 -0.83477400 0.53170000<br>H 2.14426500 -1.11954400 0.35871700<br>H -1.77118400 -1.95027300 -2.19611300<br>H -0.48920400 -0.73557800 -2.26003800<br>H -0.52657200 -2.03899800 -3.45007700<br>H 1.06713900 3.09933100 -0.74542400<br>H 3.68667300 -0.03988000 -0.99787600<br>H 3.81282100 0.47757100 0.71041200<br>H 4.41168900 1.54003400 -0.59343000<br>H -3.66014300 0.89789100 0.98714300<br>H -3.70318600 0.38958600 -0.72683300<br>H -4.23430100 2.02945800 -0.26696000<br>C -0.04840200 -1.18806300 0.61860300<br>O -0.01670500 -1.53919000 2.06055500<br>O 0.04126300 -0.36455900 2.85100800<br>H 0.97913600 -0.11753200 2.81667700	• Electronic Energy<br>= -805.273024<br>• Zero Point Energy Coorection<br>= 0.273894<br>• Thermal Correction to Energy<br>= 0.291289<br>• Thermal Correction to Enthalpy<br>= 0.292233<br>• Thermal Correction to Free Energy<br>= 0.229036

Name	Anionic HPs RAF 4-C···•OOH Product
Cartesian Coordinate:	Thermochemical Values:
O -2.34549100 -1.61392400 -0.52544100<br>O 2.34316700 -1.66334500 -0.31302000<br>O -0.00382800 -2.87936000 -0.73862500<br>C 0.01305900 2.57472900 0.16016900<br>C -0.29865900 2.77212000 -1.32601600<br>C 1.23092200 0.36867800 0.35575300<br>C -1.26201900 0.39807500 0.24326600<br>C -1.23139400 -0.89595100 -0.18064400<br>C 1.21462800 -0.92222100 -0.06514500<br>C -0.00633000 -1.65205800 -0.34244400<br>C 0.62883400 2.03918800 -2.29340500<br>C -3.60059700 -0.95922600 -0.40903000<br>C 3.59357500 -1.02954700 -0.08699300<br>H -0.72248100 3.13930900 0.74252700<br>H 1.00085400 2.98647600 0.39257200<br>H -1.33253400 2.47013700 -1.51864400<br>H -0.24911300 3.84648500 -1.52662600<br>H 2.15844200 0.90251900 0.52099100<br>H -2.19053800 0.95088800 0.31808600<br>H 1.67907900 2.24946700 -2.06982000<br>H 0.48788900 0.95715100 -2.24290000<br>H 0.43289500 2.35624700 -3.32073400<br>H -3.64906700 -0.08710400 -1.06657900<br>H -3.78559200 -0.65024000 0.62325400<br>H -4.35023600 -1.68707900 -0.71295900<br>H 3.69246600 -0.71982200 0.95687000<br>H 3.71502800 -0.16006100 -0.73907500<br>H 4.35550000 -1.77023200 -0.32174100<br>C -0.02204700 1.11580700 0.64823900<br>O -0.09744000 1.36037400 2.14004400<br>O -0.12640000 0.12541700 2.83694300<br>H -0.96250100 -0.27500700 2.54306000	• Electronic Energy<br>= -804.810402<br>• Zero Point Energy Coorection<br>= 0.261180<br>• Thermal Correction to Energy<br>= 0.278177<br>• Thermal Correction to Enthalpy<br>= 0.279121<br>• Thermal Correction to Free Energy<br>= 0.216446

Name	•OOH
Cartesian Coordinate:	Thermochemical Values:
O 0.05494100 0.70594700 0.00000000<br>O 0.05494100 -0.59539200 0.00000000<br>H -0.87906100 -0.88444000 0.00000000	• Electronic Energy = -150.903343<br>• Zero Point Energy Coorection = 0.014649<br>• Thermal Correction to Energy = 0.017498<br>• Thermal Correction to Enthalpy = 0.018443<br>• Thermal Correction to Free Energy = -0.007484

Name	OOH anion
Cartesian Coordinate:	Thermochemical Values:
O 0.05512100 0.78863800 0.00000000<br>O 0.05512100 -0.67620500 0.00000000<br>H -0.88193600 -0.89946200 0.00000000	• Electronic Energy = -151.070397<br>• Zero Point Energy Coorection = 0.013789<br>• Thermal Correction to Energy = 0.016682<br>• Thermal Correction to Enthalpy = 0.017626<br>• Thermal Correction to Free Energy = -0.007915

Name	HOOH
Cartesian Coordinate:	Thermochemical Values:
O 0.70256700 0.10244000 -0.06952600<br>H 1.01899900 -0.56502800 0.55618800<br>O -0.70256900 -0.10244700 -0.06951600<br>H -1.01898300 0.56508300 0.55614200	• Electronic Energy = -151.551595<br>• Zero Point Energy Coorection = 0.026940<br>• Thermal Correction to Energy = 0.030178<br>• Thermal Correction to Enthalpy = 0.031122<br>• Thermal Correction to Free Energy = 0.004659

4. Figure S4: Cartesian Coordinate and Thermochemical Values of all Optimized Stationary Points in Pentyl Ethanoate

Name	Hs (Syringol)
Cartesian Coordinate:	Thermochemical Values:
O 2.32429700 -0.80127400 0.00000600<br>O -2.30867500 -0.74877400 0.00000100<br>O -0.01796800 -2.04366700 0.00000600<br>C 0.03883300 2.08885500 -0.00000400<br>C 1.22296400 -0.01347800 0.00000300<br>C -1.19544700 0.03754800 0.00000000<br>C -1.18608100 1.43022400 -0.00000300<br>C 1.23782600 1.38343700 -0.00000100<br>C -0.00148600 -0.68745800 0.00000300	• Electronic Energy = -536.458159<br>• Zero Point Energy Coorection = 0.171508<br>• Thermal Correction to Energy

C 3.58656800 -0.15619000 0.00000000<br>C -3.57287500 -0.10209000 -0.00000800<br>H -2.10976200 1.99274800 -0.00000500<br>H 2.17558700 1.92162600 -0.00000100<br>H -0.94300200 -2.32245100 0.00000500<br>H 3.71649900 0.46018500 0.89450600<br>H 3.71649500 0.46017800 -0.89451100<br>H 4.32919400 -0.95170900 0.00000100<br>H -3.69534200 0.51363400 -0.89511700<br>H -3.69535200 0.51364300 0.89509300<br>H -4.31782700 -0.89510000 -0.00000800<br>H 0.06046800 3.17189500 -0.00000600	= 0.182095<br>• Thermal Correction to Enthalpy<br>= 0.183039<br>• Thermal Correction to Free Energy<br>= 0.135374

Name	Hs 1-OH radical
Cartesian Coordinate:	Thermochemical Values:
O 2.34345100 -0.78333000 0.00000200<br>O -2.34348200 -0.78330300 -0.00000100<br>O -0.00004100 -2.03932500 0.00000100<br>C 0.00004700 2.02493000 -0.00000300<br>C 1.24544400 -0.02817600 0.00000000<br>C -1.24544200 -0.02815400 -0.00000100<br>C -1.22775800 1.35464600 -0.00000300<br>C 1.22783700 1.35465500 -0.00000200<br>C -0.00005200 -0.80355700 0.00000000<br>C 3.60436300 -0.12503200 0.00000700<br>C -3.60436900 -0.12497100 -0.00000200<br>H -2.14446200 1.92794700 -0.00000300<br>H 2.14452800 1.92798000 -0.00000200<br>H 3.72048300 0.49143600 0.89531300<br>H 3.72049000 0.49143600 -0.89529900<br>H 4.35229100 -0.91448900 0.00000900<br>H -3.72048200 0.49151400 -0.89530100<br>H -3.72048400 0.49151400 0.89529600<br>H -4.35232700 -0.91440000 -0.00000300<br>H 0.00010800 3.10869300 -0.00000400	• Electronic Energy<br>= -535.820165<br>• Zero Point Energy Coorection<br>= 0.158915<br>• Thermal Correction to Energy<br>= 0.169139<br>• Thermal Correction to Enthalpy<br>= 0.170083<br>• Thermal Correction to Free Energy<br>= 0.122555

Name	Hs HT 1-OH···•OOH Transition State
Cartesian Coordinate:	Thermochemical Values:
O -2.12219700 -0.22867000 -0.55351100<br>O 2.43108500 0.75881400 -0.37432200<br>C 0.67096200 -2.29795200 0.61183500<br>C -0.89315700 -0.70167100 -0.24616800<br>C 1.49286300 -0.16387100 -0.13052800<br>C 1.73545900 -1.42199100 0.41171000<br>C -0.64118900 -1.95589700 0.29574300<br>C 0.16785200 0.21395800 -0.49480300<br>C -3.22324000 -1.13177500 -0.46531900<br>C 3.77613400 0.43749900 -0.04495600<br>H 2.73718500 -1.72648900 0.68077400<br>H -1.44277800 -2.65845700 0.47608000<br>H -3.37751100 -1.46270900 0.56520400<br>H -3.06344800 -1.99518900 -1.11483300<br>H -4.09226500 -0.57235500 -0.80395400<br>H 4.12070100 -0.43057400 -0.61323500<br>H 3.88044700 0.24689000 1.02653000<br>H 4.36516000 1.30983300 -0.31846000<br>H 0.86992400 -3.27550900 1.03507700<br>O -0.04629800 1.35968800 -1.09529700<br>H -0.70890500 2.00027300 -0.50610300<br>O -1.33147400 2.62164400 0.44438300<br>O -1.39856200 1.69329700 1.43275300<br>H -2.06304900 1.05631300 1.11577600	• Electronic Energy<br>= -687.346823<br>• Zero Point Energy Coorection<br>= 0.183531<br>• Thermal Correction to Energy<br>= 0.196820<br>• Thermal Correction to Enthalpy<br>= 0.197764<br>• Thermal Correction to Free Energy<br>= 0.142870

Name	Hs HT 1-OH···•OOH Reactant Complex
Cartesian Coordinate:	Thermochemical Values:
O -1.79182600 -0.62599900 -0.24189800<br>O 2.43787100 1.34004700 -0.17940500<br>C 1.48443300 -2.16396100 0.43585700<br>C -0.43916200 -0.83327400 -0.05895000<br>C 1.74098100 0.19311600 -0.01814000<br>C 2.30015400 -1.04204500 0.30254300<br>C 0.11185500 -2.07155100 0.25983400<br>C 0.35308300 0.30272700 -0.20850200<br>C -2.56890000 -1.73950900 -0.69867100<br>C 3.84339000 1.27913100 -0.00161800<br>H 3.36769800 -1.13748800 0.44644400<br>H -0.51930100 -2.94395900 0.36702400	• Electronic Energy<br>= -687.371619<br>• Zero Point Energy Coorection<br>= 0.189125<br>• Thermal Correction to Energy<br>= 0.203148<br>• Thermal Correction to Enthalpy

H -2.71447600 -2.46622300 0.10267000<br>H -2.07932600 -2.20926500 -1.55343400<br>H -3.53210200 -1.33255500 -1.00269900<br>H 4.30396100 0.59903400 -0.72416500<br>H 4.09964400 0.96576500 1.01471300<br>H 4.21041800 2.28931000 -0.17132400<br>H 1.92989300 -3.11969200 0.68292700<br>O -0.15736600 1.50966700 -0.53573600<br>H -1.12405500 1.46880400 -0.59615000<br>O -2.93752300 2.08486900 0.08531200<br>O -3.31750200 1.18515400 0.94970900<br>H -2.76659800 0.38856600 0.73600800	= 0.204092<br>• Thermal Correction to Free Energy<br>= 0.146639

Name	Hs HT 1-OH···•OOH Product Complex
Cartesian Coordinate:	Thermochemical Values:
O -1.76906600 -0.79078900 -0.11964700<br>O 2.36411100 1.41032200 -0.04828800<br>C 1.61804400 -2.16000900 0.13135700<br>C -0.43877500 -0.94147700 -0.04616000<br>C 1.75526700 0.23164800 -0.00650800<br>C 2.38706600 -0.99586400 0.10302500<br>C 0.21770400 -2.14814200 0.05767900<br>C 0.29627600 0.32135200 -0.09503500<br>C -2.57443700 -1.96809000 -0.20085800<br>C 3.78615700 1.43387300 0.03638900<br>H 3.46409000 -1.06708400 0.16486600<br>H -0.32542600 -3.08256900 0.08786000<br>H -2.47724000 -2.56486700 0.70906700<br>H -2.29302900 -2.55935300 -1.07478600<br>H -3.59956200 -1.61980800 -0.30781700<br>H 4.23151200 0.89215300 -0.80165900<br>H 4.12256600 1.00463300 0.98323500<br>H 4.06805900 2.48262000 -0.01233400<br>H 2.12321700 -3.11463500 0.21678300<br>O -0.27772400 1.41570100 -0.20985300<br>H -2.00181500 1.96194100 -0.53591900<br>O -2.97330500 2.01455900 -0.47103700<br>O -3.18890700 1.50310600 0.84051300<br>H -2.83705600 0.60404100 0.75787200	• Electronic Energy<br>= -687.381087<br>• Zero Point Energy Coorection<br>= 0.188893<br>• Thermal Correction to Energy<br>= 0.203146<br>• Thermal Correction to Enthalpy<br>= 0.204091<br>• Thermal Correction to Free Energy<br>= 0.146113

Name	HAs RAF 1-C···•OOH Product
Cartesian Coordinate:	Thermochemical Values:
O 2.34098700 -0.52661200 -0.37241000<br>O -2.34524500 -0.51567800 -0.30951000<br>O 0.00440000 -1.30942800 -1.48525600<br>C 0.01004800 2.26558000 0.24063000<br>C 1.24840200 0.24604500 -0.19646100<br>C -1.25017000 0.25636700 -0.14312100<br>C -1.22612300 1.59423800 0.11865300<br>C 1.23513300 1.58408300 0.07570100<br>C 3.61272800 0.11227400 -0.28123800<br>C -3.61682300 0.12823400 -0.24563200<br>H -2.14853300 2.14996400 0.22555300<br>H 2.16318800 2.13405400 0.16037800<br>H -0.88216900 -1.67564100 -1.61946400<br>H 3.74913400 0.54887800 0.71075900<br>H 3.70636300 0.88637300 -1.04623000<br>H 4.35169600 -0.66717200 -0.44867800<br>H -3.69269300 0.89505200 -1.01980000<br>H -3.76623400 0.57463900 0.73974600<br>H -4.35589700 -0.65007900 -0.41713600<br>H 0.01886200 3.32573700 0.45471600<br>C -0.00587000 -0.57139400 -0.29408700<br>O 0.01017300 -1.63140700 0.71284600<br>O -0.02314700 -1.04490700 2.00065200<br>H 0.91498500 -0.99011300 2.24289800	• Electronic Energy<br>= -687.365682<br>• Zero Point Energy Coorection<br>= 0.188913<br>• Thermal Correction to Energy<br>= 0.202297<br>• Thermal Correction to Enthalpy<br>= 0.203241<br>• Thermal Correction to Free Energy<br>= 0.148695

Name	Hs RAF 2-C···•OOH Product
Cartesian Coordinate:	Thermochemical Values:
O 1.84576900 -0.63227700 -1.06764700<br>O -2.57656800 -0.71730300 -0.20203200<br>O -0.30145800 -2.01621300 -0.08776900<br>C -0.16096600 2.10748800 -0.12691400<br>C -1.45594200 0.05868500 -0.16500600<br>C -1.42778800 1.44309100 -0.17414100<br>C 1.01317800 1.44087600 -0.07750600<br>C -0.27785900 -0.68257300 -0.10962100	• Electronic Energy<br>= -687.350404<br>• Zero Point Energy Coorection<br>= 0.188968<br>• Thermal Correction to Energy

C 3.11776900 -0.04259400 -1.32047300<br>C -3.83273000 -0.05507500 -0.24319900<br>H -2.33779700 2.02454900 -0.21386900<br>H 1.95687600 1.97198900 -0.02877300<br>H -1.22671700 -2.30239600 -0.11778700<br>H 3.68196300 0.10786900 -0.39763900<br>H 3.01356400 0.90515000 -1.85503200<br>H 3.65259800 -0.74957500 -1.95284300<br>H -3.91992600 0.55656800 -1.14516300<br>H -3.97292100 0.56702300 0.64480900<br>H -4.58689700 -0.83898300 -0.26074200<br>H -0.15696700 3.19212600 -0.13422700<br>C 1.08186000 -0.05629200 -0.05330400<br>O 1.77487900 -0.49897200 1.15535100<br>O 1.03862500 -0.08061000 2.28834800<br>H 1.30111500 0.84705100 2.39224200	= 0.202370<br>• Thermal Correction to Enthalpy = 0.203314<br>• Thermal Correction to Free Energy = 0.148871

Name	Hs RAF 3-C···•OOH Product
Cartesian Coordinate:	Thermochemical Values:
O -1.88450800 1.41767200 0.07405000<br>O 2.69135700 0.62086400 0.17872100<br>O 0.63053900 2.21735900 0.53024600<br>C 0.03013800 -1.64846400 -0.94369300<br>C -0.87993100 0.55354600 -0.19175500<br>C 1.49293400 0.07868500 -0.13711900<br>C 1.29304500 -1.21233700 -0.66343900<br>C 0.39579300 0.96027600 0.06988500<br>C -3.12617200 1.22053000 -0.59343200<br>C 3.85934900 -0.16856700 -0.01489900<br>H 2.14412500 -1.84640200 -0.87887200<br>H 1.58472400 2.31807100 0.63681800<br>H -3.59989200 0.28573000 -0.28676500<br>H -2.99229100 1.23772400 -1.67924600<br>H -3.75654500 2.05735300 -0.29884400<br>H 3.98047700 -0.42643500 -1.06985900<br>H 3.81561200 -1.07688100 0.59118300<br>H 4.69533500 0.44716600 0.30901100<br>H -0.13210200 -2.62698400 -1.37995600<br>C -1.18355500 -0.84900300 -0.61113800<br>O -1.98793300 -1.52569700 0.40977900<br>O -1.25809800 -1.57736200 1.62348800<br>H -0.66180900 -2.32856200 1.48268000<br>H -1.89810400 -0.87145900 -1.44289500	• Electronic Energy = -687.347461<br>• Zero Point Energy Coorection = 0.189210<br>• Thermal Correction to Energy = 0.202794<br>• Thermal Correction to Enthalpy = 0.203738<br>• Thermal Correction to Free Energy = 0.148862

Name	Hs RAF 4-C···•OOH Product
Cartesian Coordinate:	Thermochemical Values:
O 2.31607700 -1.30190000 0.08301400<br>O -2.31647500 -1.22163900 0.07077800<br>O -0.03463100 -2.39410900 0.57844300<br>C 1.24699900 -0.51091900 -0.15702100<br>C -1.22725500 -0.44562200 -0.16760800<br>C -1.23149400 0.82628300 -0.63883500<br>C 1.28654900 0.76238100 -0.62759400<br>C -0.00885500 -1.13791500 0.11062900<br>C 3.59616900 -0.73161400 -0.13558000<br>C -3.59360700 -0.64772400 -0.17288700<br>H -2.15140300 1.34294800 -0.88006700<br>H 2.22576500 1.25583500 -0.83715900<br>H -0.96161700 -2.65979400 0.66680500<br>H 3.74761100 0.14542500 0.50033500<br>H 3.72696100 -0.45172100 -1.18516700<br>H 4.31760300 -1.50249800 0.12726600<br>H -3.69848800 -0.37502800 -1.22617000<br>H -3.74640100 0.23329700 0.45627900<br>H -4.32331300 -1.41274600 0.08252300<br>C 0.04948600 1.57895800 -0.79982900<br>O 0.06482200 2.74331500 0.08394000<br>H 0.06640000 2.09657400 -1.76664900<br>O 0.08628400 2.31045700 1.43347300<br>H -0.83968900 2.07573600 1.59717800	• Electronic Energy = -687.353965<br>• Zero Point Energy Coorection = 0.189760<br>• Thermal Correction to Energy = 0.203074<br>• Thermal Correction to Enthalpy = 0.204018<br>• Thermal Correction to Free Energy = 0.149752

Name	HAs (4-allylsyringol)
Cartesian Coordinate:	Thermochemical Values:

O 2.84769700 -1.09059500 0.11156700<br>O 0.18658800 2.70079300 0.00630500<br>O 2.54842800 1.54869200 0.19867000<br>C -0.81697300 -0.84022200 -0.27384900<br>C -2.04502300 -1.70951400 -0.46888000<br>C -0.95454800 0.54714300 -0.21846400<br>C 0.44603300 -1.41737200 -0.16412700<br>C 1.58328300 -0.61907000 -0.00541000<br>C 0.17762500 1.33980800 -0.06224800<br>C 1.44818100 0.76862200 0.04379600<br>C -3.11860700 -1.42343800 0.54358200<br>C 3.02749900 -2.49583800 0.06899500<br>C -1.06270700 3.37211800 -0.06248800<br>C -4.34021800 -0.99452800 0.24262400<br>H -2.45022600 -1.55957100 -1.47490000<br>H -1.74388800 -2.75878400 -0.39355600<br>H -1.93838100 0.99353700 -0.29556900<br>H 0.53958900 -2.49480200 -0.20571100<br>H -2.84184600 -1.57322900 1.58580400<br>H 2.25054600 2.46739600 0.21889300<br>H 2.68962100 -2.90995700 -0.88562200<br>H 2.49964700 -2.98817600 0.89122800<br>H 4.09737900 -2.66406400 0.17619200<br>H -1.71369400 3.06828400 0.76176400<br>H -1.55752400 3.17711300 -1.01765700<br>H -0.84005200 4.43385100 0.02141100<br>H -5.07881300 -0.79935800 1.01260600<br>H -4.64133000 -0.82960000 -0.78839800	• Electronic Energy = -653.144244<br>• Zero Point Energy Coorection = 0.232857<br>• Thermal Correction to Energy = 0.247249<br>• Thermal Correction to Enthalpy = 0.248193<br>• Thermal Correction to Free Energy = 0.191127

Name	HAs 1-OH radical
Cartesian Coordinate:	Thermochemical Values:
O 2.89894000 -0.91786700 0.14312200<br>O -0.01085700 2.75268400 0.03685700<br>O 2.41940100 1.69525600 0.28807600<br>C -0.73077700 -0.82436800 -0.36123000<br>C -1.91009600 -1.73997300 -0.59539400<br>C -0.95900600 0.56090600 -0.27319900<br>C 0.55728300 -1.35677300 -0.22275900<br>C 1.63581900 -0.51781200 -0.00166100<br>C 0.09246500 1.42577400 -0.05734600<br>C 1.46552100 0.93344600 0.09302600<br>C -2.89554300 -1.67842400 0.54183200<br>C 3.17151800 -2.31159000 0.06964600<br>C -1.30491800 3.32720600 -0.08970500<br>C -4.15856500 -1.28528300 0.41647500<br>H -2.41417700 -1.45421800 -1.52425900<br>H -1.54798500 -2.76476200 -0.71476300<br>H -1.97336900 0.92685700 -0.37418900<br>H 0.69098300 -2.42833200 -0.29516900<br>H -2.51342300 -1.96987600 1.51826700<br>H 2.89111800 -2.71167900 -0.90837000<br>H 2.64357700 -2.85290000 0.85917600<br>H 4.24494800 -2.41309200 0.21212300<br>H -1.97269900 2.95838100 0.69345100<br>H -1.73127700 3.11174400 -1.07312900<br>H -1.16704200 4.40020400 0.02189200<br>H -4.82902300 -1.25616000 1.26846400<br>H -4.56370900 -0.98540000 -0.54604400	• Electronic Energy = -652.508243<br>• Zero Point Energy Coorection = 0.220403<br>• Thermal Correction to Energy = 0.234407<br>• Thermal Correction to Enthalpy = 0.235352<br>• Thermal Correction to Free Energy = 0.178201

Name	HAs 1’-CH radical
Cartesian Coordinate:	Thermochemical Values:
O -2.48793300 -1.71611600 0.00034900<br>O -0.89905200 2.63490700 -0.00013900<br>O -2.88167200 0.90862400 0.00018400<br>C 1.02576700 -0.52705500 -0.00012800<br>C 2.34764000 -1.08064100 -0.00022800<br>C 0.77897800 0.86305900 -0.00019900<br>C -0.07409400 -1.41281700 0.00004700<br>C -1.37971400 -0.93947200 0.00015600<br>C -0.52487300 1.32559400 -0.00009800<br>C -1.61310600 0.44168400 0.00007500<br>C 3.56136100 -0.36297900 -0.00036200<br>C -2.29886600 -3.12142400 0.00077500<br>C 0.12815700 3.61619700 -0.00018000<br>C 4.78747100 -0.95546400 -0.00045600<br>H 2.41559600 -2.16546700 -0.00018900<br>H 1.59948700 1.56555900 -0.00033100<br>H 0.12175200 -2.47707900 0.00009600<br>H 3.52534500 0.72320700 -0.00038900<br>H -2.83740900 1.87428100 0.00013200<br>H -1.75995200 -3.44737400 0.89534700	• Electronic Energy = -652.511563<br>• Zero Point Energy Coorection = 0.219345<br>• Thermal Correction to Energy = 0.233597<br>• Thermal Correction to Enthalpy = 0.234541<br>• Thermal Correction to Free Energy = 0.177689

H -1.76016000 -3.44796700 -0.89370400<br>H -3.29588300 -3.55741600 0.00102800<br>H 0.74938600 3.52813300 -0.89546600<br>H 0.74948600 3.52809700 0.89503200<br>H -0.37573800 4.58033900 -0.00013500<br>H 5.69687300 -0.36768900 -0.00055100<br>H 4.88814200 -2.03603300 -0.00043800	

Name	HAs HT 1-OH···•OOH Transition State
Cartesian Coordinate:	Thermochemical Values:
O -2.07707900 -1.53231700 -0.58910200<br>O 0.04560500 2.61330200 -0.36006700<br>C 1.39586000 -0.73635900 0.31355800<br>C 2.68759700 -1.39506300 0.74804500<br>C 1.35644300 0.65639500 0.18357700<br>C 0.26433100 -1.50794100 0.05094400<br>C -0.91255100 -0.88539900 -0.34986700<br>C 0.18604900 1.29001000 -0.20932000<br>C -0.97762000 0.52410900 -0.51160900<br>C 3.80595400 -1.12890100 -0.22348000<br>C -2.05594200 -2.95881100 -0.60607000<br>C 1.17903300 3.43173600 -0.10416100<br>C 4.92900900 -0.48935300 0.08536700<br>H 2.97177200 -1.02122200 1.73674300<br>H 2.52394600 -2.47269400 0.83600000<br>H 2.25267700 1.22659800 0.39277000<br>H 0.31156700 -2.58252900 0.16828200<br>H 3.64822700 -1.48484400 -1.23984400<br>H -1.80801200 -3.35940100 0.38046600<br>H -1.34143400 -3.32314000 -1.34766400<br>H -3.06213800 -3.26544500 -0.88266900<br>H 2.00051400 3.18378200 -0.78177000<br>H 1.50911700 3.32929900 0.93317300<br>H 0.85365100 4.45394600 -0.28342000<br>H 5.70506900 -0.31994000 -0.65306700<br>H 5.10968700 -0.11969200 1.09098200<br>O -2.05067400 1.11401900 -0.98427800<br>H -2.90810100 0.96302800 -0.33219000<br>O -3.71050900 0.80601000 0.68615300<br>O -2.95946500 0.09635000 1.56655300<br>H -2.89854000 -0.78921200 1.16622900	• Electronic Energy<br>= -804.034329<br>• Zero Point Energy Coorection<br>= 0.244994<br>• Thermal Correction to Energy<br>= 0.262137<br>• Thermal Correction to Enthalpy<br>= 0.263081<br>• Thermal Correction to Free Energy<br>= 0.198370

Name	HAs HT 1'-CH···•OOH Transition State
Cartesian Coordinate:	Thermochemical Values:
O 1.36631300 2.72413600 0.22128400<br>O 2.61828100 -1.69019200 -0.39109800<br>O 3.21394200 0.83003400 0.08189500<br>C -0.76997800 -0.21072800 -0.46007400<br>C 0.22843800 -1.19600300 -0.53036100<br>C -0.41252200 1.11640600 -0.19850900<br>C 0.92542900 1.46979200 -0.02116300<br>C 1.55740500 -0.84138900 -0.34541200<br>C 1.91769200 0.48746200 -0.09043600<br>C -3.26966600 0.34313700 -0.61453600<br>C 0.39634200 3.75695800 0.29722600<br>C 2.35341200 -3.06648300 -0.62882600<br>C -4.35199900 0.22333700 -1.38602500<br>H -0.04921400 -2.22390200 -0.72949500<br>H -1.17664100 1.87833700 -0.14506900<br>H -3.19671900 1.18659900 0.06755600<br>H 3.74497500 0.02751300 -0.00978800<br>H -0.30896600 3.57711600 1.11376800<br>H -0.14761200 3.85746300 -0.64645800<br>H 0.95049500 4.67249000 0.49388700<br>H 1.87761100 -3.20739700 -1.60274000<br>H 1.71902900 -3.48086800 0.15949700<br>H 3.31990300 -3.56547100 -0.61911400<br>H -5.15092300 0.95546200 -1.35419300<br>H -4.46729000 -0.61047700 -2.07228200<br>H -2.33048800 -1.26780200 0.49606600<br>O -2.15756100 -1.84849100 1.67565600<br>O -1.34009500 -0.98360300 2.34633500<br>H -0.44531200 -1.19766800 2.03176900<br>C -2.18019000 -0.64914000 -0.58052000<br>H -2.31205800 -1.45655900 -1.30416200	• Electronic Energy<br>= -804.026293<br>• Zero Point Energy Coorection<br>= 0.244680<br>• Thermal Correction to Energy<br>= 0.262099<br>• Thermal Correction to Enthalpy<br>= 0.263043<br>• Thermal Correction to Free Energy<br>= 0.198121

Name	HAs HT 1-OH···•OOH Reactant Complex
Cartesian Coordinate:	Thermochemical Values:
O 2.11044700 -1.15402300 0.26107400<br>O -0.51977400 2.70569800 0.17692500<br>C -1.50840100 -0.81152600 -0.36284800<br>C -2.74037200 -1.63484600 -0.68393500<br>C -1.61893200 0.57857700 -0.24335900<br>C -0.27022200 -1.41422800 -0.19455700<br>C 0.84382300 -0.62598600 0.09092600<br>C -0.50492000 1.36161300 0.03746500<br>C 0.75275500 0.75469200 0.21313400<br>C -3.84014900 -1.43125900 0.32129500<br>C 2.20192600 -2.41278900 0.94199800<br>C -1.76907500 3.36247900 0.03902200<br>C -5.04665400 -0.95709000 0.02799900<br>H -3.11119300 -1.37420100 -1.68029100<br>H -2.45823400 -2.69179000 -0.70767200<br>H -2.59141500 1.03843600 -0.36953200<br>H -0.16617500 -2.48858200 -0.28716300<br>H -3.59655400 -1.68494000 1.35160500<br>H 1.88956300 -3.23143900 0.29174700<br>H 1.58912200 -2.39022500 1.84471000<br>H 3.25014800 -2.53924600 1.20961100<br>H -2.48446100 3.01178300 0.78869400<br>H -2.18404700 3.21530400 -0.96243800<br>H -1.56918700 4.42058700 0.19556300<br>H -5.80529000 -0.82499700 0.79184700<br>H -5.31479200 -0.68968500 -0.99050800<br>O 1.81347300 1.53909100 0.50478000<br>H 2.63011600 1.01628700 0.51155900<br>O 4.45663800 0.57047800 -0.23805900<br>O 4.32342700 -0.47826000 -1.00163600<br>H 3.45002400 -0.86898500 -0.73524600	• Electronic Energy<br>= -804.057809<br>• Zero Point Energy Coorection<br>= 0.250274<br>• Thermal Correction to Energy<br>= 0.268268<br>• Thermal Correction to Enthalpy<br>= 0.269212<br>• Thermal Correction to Free Energy<br>= 0.201517

Name	HAs HT 1'-CH···•OOH Reactant Complex
Cartesian Coordinate:	Thermochemical Values:
O 0.01406200 2.88626400 0.17123800<br>O 2.88035300 -0.68757800 -0.49973600<br>O 2.45051200 1.84289500 0.10407300<br>C -0.79815300 -0.58243800 -0.80527800<br>C 0.49153300 -1.11452800 -0.82779100<br>C -0.98928800 0.76060600 -0.46459300<br>C 0.10059600 1.58184100 -0.16525600<br>C 1.57989400 -0.29410500 -0.52455400<br>C 1.39551700 1.05044500 -0.19771700<br>C -3.08725900 -1.38827100 -0.13822600<br>C -1.28408100 3.45335400 0.26212100<br>C 3.16841900 -2.04821700 -0.79628700<br>C -4.31265700 -0.93070700 -0.37294700<br>H 0.63899900 -2.15703800 -1.07771700<br>H -1.99651300 1.15673500 -0.43582100<br>H -2.82932400 -1.74473000 0.85782900<br>H 3.24850200 1.29991800 0.05520000<br>H -1.88449600 2.94066100 1.01919400<br>H -1.79833600 3.41968500 -0.70257800<br>H -1.13651500 4.49008500 0.55730800<br>H 2.84741700 -2.30008500 -1.81040900<br>H 2.68718600 -2.71294300 -0.07427400<br>H 4.24884100 -2.14984100 -0.72070400<br>H -5.06825100 -0.90922200 0.40484000<br>H -4.59992400 -0.56761100 -1.35601500<br>H -1.65130200 -2.47969200 -1.27847800<br>O 0.27136700 -2.07150100 2.42816100<br>O -0.08559700 -0.81891600 2.35598200<br>H -0.19466600 -0.61724100 1.40142700<br>C -1.99230800 -1.44632100 -1.16650600<br>H -2.39048300 -1.12793800 -2.13534500	• Electronic Energy<br>= -804.056129<br>• Zero Point Energy Coorection<br>= 0.249099<br>• Thermal Correction to Energy<br>= 0.267776<br>• Thermal Correction to Enthalpy<br>= 0.268720<br>• Thermal Correction to Free Energy<br>= 0.200009

Name	HAs HT 1-OH···•OOH Product Complex
Cartesian Coordinate:	Thermochemical Values:
O -2.02547700 -1.29655300 -0.10680300<br>O 0.44391300 2.68073700 -0.00557700<br>C 1.58360000 -0.78830500 0.32422000<br>C 2.86153700 -1.56658300 0.53246700<br>C 1.64895200 0.61280100 0.25638500<br>C 0.35947600 -1.46583500 0.20394600<br>C -0.80619200 -0.75289500 0.02004900	• Electronic Energy<br>= -804.069443<br>• Zero Point Energy Coorection<br>= 0.250390

C 0.50011800 1.35457600 0.07323400<br>C -0.80338900 0.70396800 -0.06481800<br>C 3.86822600 -1.29597900 -0.55432000<br>C -2.12564100 -2.71980600 -0.16909100<br>C 1.66465400 3.40658200 0.09651200<br>C 5.06886000 -0.76751500 -0.34415600<br>H 3.29414600 -1.29266100 1.50045000<br>H 2.62836400 -2.63418800 0.56378300<br>H 2.61498800 1.09332800 0.34748600<br>H 0.34844600 -2.54645800 0.26308200<br>H 3.55866000 -1.55313300 -1.56535800<br>H -1.80357900 -3.17066200 0.77231200<br>H -1.52947500 -3.10610700 -0.99850300<br>H -3.17793400 -2.93571800 -0.34183500<br>H 2.34709600 3.12953800 -0.71104600<br>H 2.13966000 3.22994700 1.06480900<br>H 1.39425300 4.45581900 0.00600400<br>H 5.76040900 -0.59272300 -1.16106700<br>H 5.40062200 -0.49919600 0.65507200<br>O -1.84130100 1.36020300 -0.25043100<br>H -3.57010000 0.93494500 -0.66501600<br>O -4.44152300 0.49638000 -0.64777400<br>O -4.46349300 -0.01820600 0.68006700<br>H -3.70370700 -0.61927700 0.66738500	• Thermal Correction to Energy<br>= 0.268418<br>• Thermal Correction to Enthalpy<br>= 0.269362<br>• Thermal Correction to Free Energy<br>= 0.202195

Name	HAs HT 1'-CH···•OOH Product Complex
Cartesian Coordinate:	Thermochemical Values:
O 1.45805600 2.68214200 0.22614900<br>O 2.45027900 -1.78742800 -0.45950700<br>O 3.18070800 0.67809800 0.11404300<br>C -0.83968900 -0.08966100 -0.63635800<br>C 0.10840600 -1.13554500 -0.69453600<br>C -0.39842500 1.21471500 -0.32483300<br>C 0.94392800 1.47049200 -0.07641600<br>C 1.44580300 -0.87227400 -0.44114300<br>C 1.87812600 0.42414500 -0.13152000<br>C -3.31903500 0.46149900 -0.72529300<br>C 0.55598800 3.77377400 0.31415400<br>C 2.10085700 -3.14324400 -0.70842300<br>C -4.60893900 0.08401400 -0.94826600<br>H -0.22458200 -2.13866200 -0.92784900<br>H -1.11217700 2.02446900 -0.27888000<br>H -3.13497300 1.48730400 -0.41808800<br>H 3.66350700 -0.15616200 0.03711900<br>H -0.18685400 3.60810700 1.09987000<br>H 0.05244700 3.94724700 -0.64125000<br>H 1.16167200 4.64173200 0.56625900<br>H 1.65896700 -3.25327500 -1.70205600<br>H 1.40589000 -3.50888800 0.05243000<br>H 3.02923800 -3.70770100 -0.65758800<br>H -5.42822500 0.78118100 -0.82590300<br>H -4.85167100 -0.92795700 -1.25630300<br>H -2.44577100 -1.31268600 1.48077900<br>O -1.81487300 -1.87274800 1.95845600<br>O -0.87567600 -0.92767500 2.44842900<br>H -0.22766800 -0.88743700 1.72729300<br>C -2.21936700 -0.41127700 -0.86097800<br>H -2.43367400 -1.43620400 -1.15472300	• Electronic Energy<br>= -804.066464<br>• Zero Point Energy Coorection<br>= 0.248445<br>• Thermal Correction to Energy<br>= 0.267234<br>• Thermal Correction to Enthalpy<br>= 0.268179<br>• Thermal Correction to Free Energy<br>= 0.199518

Name	HAs RAF 1-C···•OOH Product
Cartesian Coordinate:	Thermochemical Values:
O -2.35166300 -1.59913800 -0.41075800<br>O -0.18754800 2.55593200 -0.33220600<br>O -1.95127300 0.85993100 -1.50257700<br>C 1.18628800 -0.81344200 0.30037900<br>C 2.49356400 -1.48289600 0.64552300<br>C 1.14624900 0.59874000 0.16056600<br>C 0.01315900 -1.57193000 0.09647900<br>C -1.17945900 -0.97271900 -0.20904700<br>C -0.01980500 1.23410600 -0.13002900<br>C 3.55124500 -1.22421300 -0.39518600<br>C -2.35084700 -3.01776100 -0.37411900<br>C 0.95937000 3.38713600 -0.22186400<br>C 4.69067900 -0.58024700 -0.16304400<br>H 2.85400500 -1.12201400 1.61563700<br>H 2.32777900 -2.56023500 0.73826600<br>H 2.06711500 1.15897300 0.27443700<br>H 0.07209100 -2.65006100 0.18421100<br>H 3.32821900 -1.58327800 -1.39831400<br>H -1.90381900 1.82261700 -1.57991900<br>H -2.05028600 -3.38307000 0.61238300	• Electronic Energy<br>= -804.046809<br>• Zero Point Energy Coorection<br>= 0.250158<br>• Thermal Correction to Energy<br>= 0.267492<br>• Thermal Correction to Enthalpy<br>= 0.268436<br>• Thermal Correction to Free Energy<br>= 0.203747

H -1.68224300 -3.42554200 -1.13765900<br>H -3.37348700 -3.32539800 -0.58117600<br>H 1.71600200 3.10059600 -0.95755100<br>H 1.37980100 3.32821600 0.78558800<br>H 0.61934500 4.40140400 -0.41863100<br>H 5.41701800 -0.41115300 -0.95063200<br>H 4.93485400 -0.20574200 0.82742500<br>C -1.33294700 0.51880500 -0.30517700<br>O -2.29300300 0.99233100 0.68422900<br>O -1.77659200 0.75381900 1.98038500<br>H -2.17071800 -0.10178600 2.20647300	

Name	HAs RAF 2-C···•OOH Product
Cartesian Coordinate:	Thermochemical Values:
O -2.81736700 -1.41780200 -0.19109800<br>O -0.22518000 2.21943200 -1.02304800<br>O -2.58635800 1.22897900 -0.21667200<br>C 0.89561500 -1.02265500 -0.10275500<br>C 2.10553100 -1.93668100 -0.06360900<br>C 1.01437700 0.32194600 -0.07826000<br>C -0.39550800 -1.66119800 -0.12552500<br>C -1.56096200 -0.90973500 -0.14852000<br>C -1.47659100 0.48065500 -0.15480100<br>C 3.39133100 -1.28079400 -0.47026400<br>C -2.94325700 -2.82973500 -0.22949100<br>C 0.97149900 2.96931600 -1.22658900<br>C 4.44796000 -1.14259300 0.32314700<br>H 2.20196300 -2.34753600 0.94736200<br>H 1.90941400 -2.78504400 -0.72917500<br>H 1.98445400 0.79969200 -0.01005200<br>H -0.43547200 -2.74236900 -0.13558300<br>H 3.43264100 -0.90225000 -1.49028400<br>H -2.31906100 2.15170400 -0.34486700<br>H -2.51555000 -3.28964800 0.66634200<br>H -2.46434500 -3.24485700 -1.12118100<br>H -4.01144100 -3.03421700 -0.26503400<br>H 1.71291500 2.37451200 -1.76524400<br>H 1.38648800 3.31978300 -0.27992400<br>H 0.68834600 3.82593100 -1.83602900<br>H 5.35992700 -0.66914500 -0.02426000<br>H 4.43378300 -1.50093300 1.34869700<br>C -0.17572600 1.21335400 -0.04785300<br>O -0.19839700 2.02228300 1.18105800<br>O -0.16135000 1.16815600 2.30765700<br>H -1.10046700 1.06470800 2.52317300	• Electronic Energy = -804.037247<br>• Zero Point Energy Coorection = 0.249870<br>• Thermal Correction to Energy = 0.267258<br>• Thermal Correction to Enthalpy = 0.268202<br>• Thermal Correction to Free Energy = 0.204039

Name	HAs RAF 3-C···•OOH Product
Cartesian Coordinate:	Thermochemical Values:
O -3.04519300 -1.03710500 -0.18991800<br>O -0.16549300 2.62411000 -0.28366400<br>O -2.61360500 1.55442500 -0.47850500<br>C 0.62463600 -1.03467900 0.13386600<br>C 1.80656200 -1.94931400 0.32502900<br>C -0.65826500 -1.50490000 0.06591500<br>C -1.76070900 -0.64716500 -0.12618200<br>C -0.27538000 1.25920800 -0.22553800<br>C -1.53572400 0.75479200 -0.28106500<br>C 2.69160500 -1.96817900 -0.89521700<br>C -3.32997300 -2.41936200 -0.02427800<br>C 0.96963600 3.14067800 -0.97806300<br>C 3.92155400 -1.46551600 -0.93662300<br>H 2.39243300 -1.61789600 1.18857900<br>H 1.44267500 -2.95924400 0.53350800<br>H -0.82345100 -2.57190400 0.15724200<br>H 2.25418800 -2.40140400 -1.79312600<br>H -2.28485100 2.46072300 -0.54950000<br>H -2.98799000 -2.77446400 0.95154700<br>H -2.86908400 -3.01223400 -0.81892600<br>H -4.41230800 -2.51007700 -0.08560500<br>H 1.02982200 2.71878800 -1.98530400<br>H 1.89044800 2.93437800 -0.42815900<br>H 0.82198500 4.21711100 -1.04271000<br>H 4.51293300 -1.48613200 -1.84584200<br>H 4.37725200 -1.02089500 -0.05582700<br>C 0.92853000 0.42954400 0.05381800<br>O 1.60924900 0.91187400 1.25124500<br>H 1.72627500 0.61060600 -0.68154400<br>O 0.76478900 0.73366100 2.37693300<br>H 0.23685900 1.54629000 2.37697900	• Electronic Energy = -804.038164<br>• Zero Point Energy Coorection = 0.250717<br>• Thermal Correction to Energy = 0.268161<br>• Thermal Correction to Enthalpy = 0.269105<br>• Thermal Correction to Free Energy = 0.203992

Name	HAs RAF 4-C···•OOH Product
Cartesian Coordinate:	Thermochemical Values:
O -2.84642300 0.29638800 -0.66627900<br>O 1.17377900 2.55671400 -0.20095800<br>O -1.37093700 2.48188500 -0.80225200<br>C 1.18160700 -2.24537200 -0.01600400<br>C 1.20015900 0.22054100 0.44064100<br>C -0.98862000 -0.99117200 0.18067000<br>C -1.56381100 0.15742900 -0.26687300<br>C 0.59283000 1.34110100 -0.01246800<br>C -0.79165300 1.35481400 -0.36386400<br>C 1.31871400 -2.09832800 -1.50218900<br>C -3.67114600 -0.85503200 -0.59323400<br>C 2.54932600 2.67754900 0.13467400<br>C 2.48017700 -2.09813700 -2.14784400<br>H 2.16779400 -2.33235500 0.44813700<br>H 0.61415100 -3.15229000 0.22067700<br>H 2.25055700 0.20448100 0.70298600<br>H -1.54682000 -1.91810500 0.23332800<br>H 0.39325800 -1.98588200 -2.06390800<br>H -0.69629400 3.17683900 -0.80845800<br>H -3.74573800 -1.21983700 0.43567200<br>H -3.29078200 -1.65216800 -1.23869000<br>H -4.65392300 -0.54336200 -0.94082600<br>H 3.15569300 2.00829500 -0.48149700<br>H 2.70782400 2.45499100 1.19309500<br>H 2.82154200 3.71090500 -0.06870300<br>H 2.52736400 -1.99574200 -3.22655800<br>H 3.42237200 -2.20365800 -1.61716100<br>C 0.44077700 -1.05365000 0.62892100<br>O 0.47794900 -1.44637500 2.04126100<br>O -0.12748700 -0.44413800 2.83624200<br>H -1.07221800 -0.56635200 2.65321000	• Electronic Energy<br>= -804.043317<br>• Zero Point Energy Coorection<br>= 0.250465<br>• Thermal Correction to Energy<br>= 0.267600<br>• Thermal Correction to Enthalpy<br>= 0.268544<br>• Thermal Correction to Free Energy<br>= 0.205224

Name	HAs RAF 2'-C···•OOH Product
Cartesian Coordinate:	Thermochemical Values:
O -1.73913100 2.58931500 -0.01221000<br>O -2.72051800 -1.92549300 0.34688300<br>O -3.41038200 0.60878300 0.57054400<br>C 0.41993800 -0.31528500 -0.73975800<br>C 1.82238000 -0.65430000 -1.18164700<br>C -0.47071900 -1.34205200 -0.42738200<br>C 0.02204000 1.01429700 -0.61097900<br>C -1.26394400 1.33079600 -0.16915300<br>C -1.75265300 -1.02464600 0.01358700<br>C -2.15777000 0.30426000 0.14494200<br>C -0.85135700 3.65915300 -0.29019400<br>C -2.39444600 -3.30418800 0.26171100<br>C 4.14352900 -1.27632700 -0.45508100<br>H 1.82820900 -1.61903800 -1.69466300<br>H 2.20044600 0.09168500 -1.88704100<br>H -0.16189100 -2.37479000 -0.53306800<br>H 0.72872300 1.79933200 -0.84430600<br>H -3.86872700 -0.22460000 0.73918800<br>H -0.53489900 3.64921600 -1.33748100<br>H 0.02756000 3.62284100 0.36019000<br>H -1.41064500 4.57139400 -0.09156300<br>H -1.56226900 -3.54949300 0.92726200<br>H -2.14326100 -3.58244600 -0.76546700<br>H -3.28388300 -3.84571500 0.57710100<br>H 4.45602600 -2.28330500 -0.21383800<br>H 4.74365200 -0.68766600 -1.14049700<br>C 2.82205900 -0.75377900 -0.01606400<br>H 2.39881200 -1.37237700 0.78153100<br>O 2.94323300 0.58936000 0.47322800<br>O 3.67137800 0.55896100 1.69068400<br>H 4.59114800 0.60998000 1.38973500	• Electronic Energy<br>= -804.055073<br>• Zero Point Energy Coorection<br>= 0.249893<br>• Thermal Correction to Energy<br>= 0.267409<br>• Thermal Correction to Enthalpy<br>= 0.268353<br>• Thermal Correction to Free Energy<br>= 0.202947

Name	HAs RAF 3'-C···•OOH Product
Cartesian Coordinate:	Thermochemical Values:
O -2.66542900 -1.85809700 -0.43807200<br>O -1.49140000 2.59869400 0.02458600<br>O -3.19115100 0.72702200 -0.72405300<br>C 0.43877400 -0.41108100 0.94155500<br>C 1.78463200 -0.84703800 1.50346000<br>C 0.14371200 0.94267900 0.78856400<br>C -0.48528400 -1.37597900 0.53681500<br>C -1.70745000 -0.99865800 -0.02119900<br>C -1.07754600 1.31761300 0.22779800	• Electronic Energy<br>= -804.058567<br>• Zero Point Energy Coorection<br>= 0.251705<br>• Thermal Correction to Energy

C -2.00449900 0.35873900 -0.17970000<br>C 2.66672300 -1.42998200 0.44307500<br>C -2.40118800 -3.24491900 -0.30668600<br>C -0.59107700 3.64586100 0.35832900<br>H 2.27802900 0.01096800 1.96694900<br>H 1.60940700 -1.59132800 2.28897800<br>H 0.85779400 1.69022200 1.11018300<br>H -0.24568000 -2.42414700 0.66571800<br>H 2.36490900 -2.36473400 -0.01895400<br>H -3.20929600 1.69252600 -0.75439800<br>H -2.26025000 -3.52295700 0.74197400<br>H -1.52052100 -3.53644400 -0.88666300<br>H -3.27782200 -3.75525200 -0.70055100<br>H 0.33323000 3.56345900 -0.22031900<br>H -0.36354300 3.63794200 1.42756300<br>H -1.09926200 4.57314700 0.10226500<br>C 3.82042200 -0.70602100 -0.15185400<br>O 3.50790100 -0.07737700 -1.40830700<br>H 4.62279100 -1.39660400 -0.42757000<br>H 4.21582100 0.05519300 0.52737400<br>O 2.64141600 1.01632400 -1.14635900<br>H 1.76038500 0.60820000 -1.12584900	= 0.268737<br>• Thermal Correction to Enthalpy = 0.269681<br>• Thermal Correction to Free Energy = 0.205049

Name	HPns (4-propenylsyringol)
Cartesian Coordinate:	Thermochemical Values:
O -2.41840400 -1.86018800 -0.02928400<br>O -1.13034200 2.58925800 0.00184100<br>O -2.99663200 0.73535100 -0.06397100<br>C 0.99305900 -0.43113900 0.06215100<br>C -1.37007200 -1.00315500 -0.00366400<br>C -0.67271600 1.30582900 0.00975600<br>C 0.66274900 0.92885300 0.05453000<br>C -0.02825600 -1.38564000 0.04108700<br>C -1.69627500 0.35321600 -0.02322700<br>C 2.38864900 -0.90609600 0.09140800<br>C 3.48968300 -0.17453200 -0.09526900<br>C -2.12900900 -3.24768100 -0.00153200<br>C -0.16929300 3.63388600 0.02809400<br>C 4.88092100 -0.72540900 -0.05046100<br>H 1.43781100 1.68179200 0.09698300<br>H 0.23801000 -2.43457900 0.05373400<br>H 2.50451100 -1.97384700 0.27141800<br>H 3.40241400 0.89054100 -0.30067300<br>H -3.01478400 1.70133300 -0.06500000<br>H -1.59684400 -3.52302200 0.91392200<br>H -1.54035100 -3.54709200 -0.87374100<br>H -3.09175600 -3.75449900 -0.02511700<br>H 0.48183400 3.58568900 -0.84895400<br>H 0.43149500 3.58762400 0.94039600<br>H -0.73383500 4.56381100 0.01143600<br>H 5.40013500 -0.55188600 -0.99801900<br>H 5.47086800 -0.23142800 0.72786600<br>H 4.87686400 -1.79859500 0.14981200	• Electronic Energy = -653.152044<br>• Zero Point Energy Coorection = 0.232584<br>• Thermal Correction to Energy = 0.247290<br>• Thermal Correction to Enthalpy = 0.248234<br>• Thermal Correction to Free Energy = 0.189859

Name	HPns 1-OH radical
Cartesian Coordinate:	Thermochemical Values:
O 2.56234400 -1.69343500 -0.00004000<br>O 0.92673000 2.69846800 -0.00010700<br>O 2.92244000 0.94428600 -0.00018800<br>C -0.91379300 -0.48609500 0.00015100<br>C 1.47098500 -0.92375400 0.00000600<br>C 0.59950200 1.40253400 -0.00001700<br>C -0.68208800 0.91033900 0.00012100<br>C 0.17340400 -1.38559200 0.00011500<br>C 1.76865300 0.51182200 -0.00007300<br>C -2.25679800 -1.04982500 0.00018700<br>C -3.41389300 -0.36901900 -0.00013600<br>C 2.38067300 -3.10281600 0.00001700<br>C -0.12878200 3.64894200 0.00014800<br>C -4.76090500 -1.01299400 -0.00015600<br>H -1.52417000 1.58761600 0.00019500<br>H -0.03955900 -2.44690900 0.00014700<br>H -2.30153800 -2.13711600 0.00046300<br>H -3.40530800 0.71821100 -0.00044200<br>H 1.84185600 -3.42586900 -0.89499100<br>H 1.84197800 -3.42581200 0.89511900<br>H 3.37968500 -3.53278200 -0.00004100<br>H -0.74846400 3.54313600 0.89516500<br>H -0.74877100 3.54329300 -0.89467600<br>H 0.34860200 4.62617800 0.00014900<br>H -5.33636800 -0.70164500 0.87728100	• Electronic Energy = -652.519011<br>• Zero Point Energy Coorection = 0.220157<br>• Thermal Correction to Energy = 0.234335<br>• Thermal Correction to Enthalpy = 0.235279<br>• Thermal Correction to Free Energy = 0.178404

H -5.33586100 -0.70255600 -0.87825500<br>H -4.68593600 -2.10156100 0.00040000	

Name	HPns 3’-CH radical
Cartesian Coordinate:	Thermochemical Values:
O 2.48793300 -1.71611500 -0.00004300<br>O 0.89905000 2.63490700 0.00015900<br>O 2.88167200 0.90862500 0.00009700<br>C -1.02576700 -0.52705500 -0.00007200<br>C 1.37971400 -0.93947100 -0.00003400<br>C 0.52487200 1.32559500 0.00006200<br>C -0.77897800 0.86305800 0.00000200<br>C 0.07409400 -1.41281700 -0.00008200<br>C 1.61310600 0.44168500 0.00003800<br>C -2.34764000 -1.08064200 -0.00012600<br>C -3.56136100 -0.36298000 -0.00021100<br>C 2.29886700 -3.12142400 -0.00008800<br>C -0.12815900 3.61619700 0.00039300<br>C -4.78747000 -0.95546500 -0.00021600<br>H -1.59948800 1.56555700 0.00002200<br>H -0.12175100 -2.47708000 -0.00013300<br>H -2.41559600 -2.16546800 -0.00010000<br>H -3.52534500 0.72320700 -0.00028500<br>H 2.83740900 1.87428200 0.00016400<br>H 1.76007600 -3.44764600 -0.89463300<br>H 1.76004000 -3.44769600 0.89441800<br>H 3.29588400 -3.55741500 -0.00008000<br>H -0.74939900 3.52793500 0.89565100<br>H -0.74947600 3.52829500 -0.89484800<br>H 0.37573600 4.58034000 0.00056200<br>H -5.69687300 -0.36769100 -0.00029400<br>H -4.88814200 -2.03603400 -0.00014000	• Electronic Energy = -652.511563<br>• Zero Point Energy Coorection = 0.219345<br>• Thermal Correction to Energy = 0.233597<br>• Thermal Correction to Enthalpy = 0.234541<br>• Thermal Correction to Free Energy = 0.177689

Name	HPns HT 1-OH···•OOH Transition State
Cartesian Coordinate:	Thermochemical Values:
O -1.33307900 2.61443900 -0.25070400<br>O -1.23881700 -2.03063400 -0.63720400<br>C 1.53957500 0.31852300 -0.07103000<br>C -0.56256700 1.51796200 -0.24538300<br>C -0.49465500 -0.90878100 -0.47195200<br>C 0.87504800 -0.89997000 -0.28155800<br>C 0.81308100 1.51768300 -0.05523700<br>C -1.25123100 0.29676000 -0.48066400<br>C 2.98931000 0.39617000 0.14577100<br>C 3.83563700 -0.63263300 0.26474300<br>C -0.70158700 3.87013800 -0.04170400<br>C -0.55178600 -3.26871100 -0.80836400<br>C 5.30603500 -0.48541200 0.48572100<br>H 1.43164000 -1.82608800 -0.29626200<br>H 1.34516200 2.44457500 0.11383400<br>H 3.38829000 1.40562900 0.22333200<br>H 3.46197600 -1.65238200 0.20597000<br>H 0.03122000 4.07523000 -0.82694600<br>H -0.21794700 3.90662900 0.93812200<br>H -1.49599900 4.61196100 -0.08283700<br>H 0.00634100 -3.53586800 0.09308200<br>H 0.12384500 -3.21741900 -1.66527600<br>H -1.32414800 -4.01191400 -0.99312000<br>H 5.60805000 -0.98110600 1.41343400<br>H 5.86660900 -0.96421000 -0.32316100<br>H 5.60016300 0.56403700 0.53929900<br>O -2.53299100 0.30599600 -0.76514800	• Electronic Energy = -804.043097<br>• Zero Point Energy Coorection = 0.245032<br>• Thermal Correction to Energy = 0.262310<br>• Thermal Correction to Enthalpy = 0.263255<br>• Thermal Correction to Free Energy = 0.199042

H -3.10440900 -0.28208400 -0.06253300<br>O -3.59400700 -0.86191000 1.01953400<br>O -2.48337700 -1.15456400 1.74062300<br>H -2.04380100 -1.85397600 1.22420200	

Name	HAs HT 3'-CH···•OOH Transition State
Cartesian Coordinate:	Thermochemical Values:
O 3.05885500 -1.86133100 0.27032000<br>O 1.78883600 2.58317600 0.01076700<br>O 3.61404000 0.73108400 0.37883500<br>C -0.28611300 -0.43972700 -0.42474900<br>C 2.02928300 -1.01141200 0.05503600<br>C 1.34017900 1.30305700 -0.08900600<br>C 0.03409200 0.92498200 -0.35602800<br>C 0.71732000 -1.39609800 -0.21275500<br>C 2.34547000 0.34858600 0.11562400<br>C -1.63751200 -0.91272100 -0.70440800<br>C -2.70689100 -0.16181200 -1.06910700<br>C 2.77888000 -3.25106200 0.22668200<br>C 0.85105500 3.63143300 -0.19229200<br>H -0.72932700 1.67740000 -0.49775500<br>H 0.45672400 -2.44525900 -0.26571700<br>H -1.78194200 -1.98857300 -0.61813200<br>H -2.59743500 0.91242000 -1.18738100<br>H 3.63407000 1.69766700 0.38396500<br>H 2.41038500 -3.54962700 -0.75911200<br>H 2.05105000 -3.53177800 0.99360500<br>H 3.72387600 -3.75252200 0.42544700<br>H 0.04917300 3.58425800 0.54911000<br>H 0.43060000 3.58581100 -1.20045700<br>H 1.40568600 4.55929600 -0.07053300<br>H -4.56353200 -0.56863900 -0.10658600<br>O -4.81790500 -0.38854900 1.16948400<br>O -3.79604600 0.43306700 1.56866600<br>H -3.07084200 -0.16931300 1.79820800<br>C -4.03492100 -0.71085200 -1.23953400<br>H -4.70345000 -0.14874500 -1.88935500<br>H -4.08233100 -1.78819400 -1.39665400	• Electronic Energy<br>= -804.031832<br>• Zero Point Energy Coorection<br>= 0.244659<br>• Thermal Correction to Energy<br>= 0.262020<br>• Thermal Correction to Enthalpy<br>= 0.262965<br>• Thermal Correction to Free Energy<br>= 0.197705

Name	HPns RAF 2'-C···•OOH Transition State
Cartesian Coordinate:	Thermochemical Values:
O -2.92049800 -1.91246800 0.14839500<br>O -1.75208500 2.56766800 0.03639900<br>O -3.54239900 0.66338500 0.29774700<br>C 0.41349400 -0.39453500 -0.39320500<br>C -1.90415800 -1.03336700 -0.00809300<br>C -1.26784800 1.30105400 -0.07493500<br>C 0.05449400 0.96154500 -0.30716600<br>C -0.57586600 -1.38006800 -0.23597100<br>C -2.25589900 0.31779900 0.07340900<br>C 1.77718100 -0.82412600 -0.63236300<br>C -2.60615700 -3.29317100 0.07042300<br>C -0.83363700 3.64217100 -0.11064900<br>C 4.19273300 -0.57668500 -1.24842400<br>H 0.80352100 1.73456200 -0.40606500<br>H -0.28687100 -2.42097200 -0.30152300<br>H 1.94687100 -1.89852200 -0.59422000<br>H -3.58614100 1.62889200 0.32295300<br>H -1.90219700 -3.58321100 0.85597900<br>H -2.19209200 -3.54986100 -0.90914700<br>H -3.54585000 -3.82237000 0.21490900<br>H -0.37881000 3.62993000 -1.10479100<br>H -0.05602600 3.59592600 0.65614900<br>H -1.41424200 4.55373600 0.01322700<br>H 4.25365300 -0.61169900 -2.34097200<br>H 5.01064100 0.04758900 -0.88660400<br>H 4.32910900 -1.58976800 -0.86637200<br>C 2.86910000 -0.01604200 -0.82941400<br>O 3.31226400 0.49708500 1.08926400<br>H 2.71608400 1.04106700 -1.02109300<br>O 3.89655800 -0.56565700 1.72035900<br>H 3.17101100 -0.98285500 2.20859800	• Electronic Energy<br>= -804.041040<br>• Zero Point Energy Coorection<br>= 0.249026<br>• Thermal Correction to Energy<br>= 0.266485<br>• Thermal Correction to Enthalpy<br>= 0.267429<br>• Thermal Correction to Free Energy<br>= 0.202340

Name	HPns HT 1-OH···•OOH Reactant Complex
Cartesian Coordinate:	Thermochemical Values:

O -0.38430700 2.99392100 -0.02768000<br>O -1.58782100 -1.54751900 -0.32882300<br>C 1.74586600 -0.03542400 -0.03640200<br>C 0.04569500 1.71326100 -0.06002800<br>C -0.58302500 -0.59732500 -0.20848300<br>C 0.74399800 -0.99688000 -0.14837600<br>C 1.37830100 1.31940300 -0.00753500<br>C -0.96489800 0.73978600 -0.15622800<br>C 3.17762200 -0.37629200 0.05199800<br>C 3.69369800 -1.58304100 0.29537900<br>C 0.59701300 4.01034400 0.09472000<br>C -1.92662200 -1.86187300 -1.69149600<br>C 5.16022400 -1.87031500 0.37406000<br>H 0.96734000 -2.05587700 -0.20204400<br>H 2.15840700 2.06620700 0.07228700<br>H 3.85989500 0.46184100 -0.08101500<br>H 3.02967500 -2.42942000 0.45962200<br>H 1.27981700 4.00591900 -0.75999100<br>H 1.16629500 3.89916100 1.02233900<br>H 0.05120800 4.95139300 0.11569900<br>H -1.06367500 -2.30634800 -2.19035300<br>H -2.24319600 -0.95984800 -2.22016800<br>H -2.74539300 -2.57969000 -1.65653700<br>H 5.42327000 -2.28460800 1.35209500<br>H 5.45111300 -2.61565800 -0.37265400<br>H 5.75233500 -0.96815100 0.20938600<br>O -2.25298200 1.13955500 -0.21802600<br>H -2.84712400 0.39653600 -0.01940800<br>O -4.15187800 -0.76293400 0.86203300<br>O -3.61207100 -1.87767100 1.26662300<br>H -2.73472000 -1.91414500 0.79206800	• Electronic Energy<br>= -804.066095<br>• Zero Point Energy Coorection<br>= 0.249949<br>• Thermal Correction to Energy<br>= 0.268018<br>• Thermal Correction to Enthalpy<br>= 0.268963<br>• Thermal Correction to Free Energy<br>= 0.202143

Name	HPns HT 3’-CH⋯•OOH Reactant Complex
Cartesian Coordinate:	Thermochemical Values:
O 2.05960800 -2.45503300 -0.19819500<br>O 2.50553700 2.11842200 0.37923300<br>O 3.51137500 -0.31171900 0.39345700<br>C -0.49533400 0.19000400 -0.56511100<br>C 1.42293600 -1.26211900 -0.23372800<br>C 1.62747400 1.12577300 0.06720000<br>C 0.28880800 1.30629900 -0.25530100<br>C 0.07772200 -1.08503600 -0.55969600<br>C 2.20341800 -0.14890800 0.08408700<br>C -1.92960900 0.30409300 -0.88906800<br>C -2.73383900 1.33628700 -0.60125500<br>C 1.29788900 -3.61062800 -0.50912600<br>C 2.01488300 3.45090800 0.40771500<br>H -0.13563800 2.30063500 -0.28898600<br>H -0.54153800 -1.93860100 -0.80457200<br>H -2.36234600 -0.56084800 -1.38975100<br>H -2.32882100 2.20306700 -0.08117800<br>H 3.87920400 0.56289100 0.57667100<br>H 0.90913300 -3.56469200 -1.53066700<br>H 0.47083600 -3.74018200 0.19515100<br>H 1.98237400 -4.45192000 -0.42126200<br>H 1.21997400 3.55600100 1.15105200<br>H 1.64716600 3.75189100 -0.57695100<br>H 2.85922200 4.07752600 0.68693300<br>H -4.80419000 1.55839800 -0.07132100<br>O -3.55995800 -1.72145800 1.18512300<br>O -3.09971600 -0.67589500 1.81597400<br>H -2.76094600 -0.07176100 1.11361900<br>C -4.18677600 1.39145300 -0.95935400<br>H -4.38225400 2.22612800 -1.63954300<br>H -4.51237100 0.46816700 -1.44210700	• Electronic Energy<br>= -804.062955<br>• Zero Point Energy Coorection<br>= 0.249232<br>• Thermal Correction to Energy<br>= 0.267812<br>• Thermal Correction to Enthalpy<br>= 0.268756<br>• Thermal Correction to Free Energy<br>= 0.200081

Name	HPns RAF 2’-C⋯•OOH Reactant Complex
Cartesian Coordinate:	Thermochemical Values:
O -3.21204200 -1.41663400 0.28858300<br>O -1.09600500 2.65098600 -0.35824300<br>O -3.26742000 1.22808400 0.06373300<br>C 0.37250200 -0.73368200 -0.29753800<br>C -2.02802300 -0.80250900 0.06087900<br>C -0.90206700 1.30605400 -0.28385600<br>C 0.32182100 0.66063500 -0.40152500<br>C -0.79959400 -1.45491200 -0.05355500<br>C -2.08047600 0.58767800 -0.05694000<br>C 1.64137500 -1.47645900 -0.42302400<br>C -3.19927100 -2.82731400 0.43103800<br>C 0.04764300 3.47549500 -0.53392100<br>C 4.04648000 -1.81164400 -1.04714200	• Electronic Energy<br>= -804.063226<br>• Zero Point Energy Coorection<br>= 0.249278<br>• Thermal Correction to Energy<br>= 0.267827<br>• Thermal Correction to Enthalpy

H 1.22745200 1.23139600 -0.55876600<br>H -0.74382600 -2.53213300 0.03568400<br>H 1.61717700 -2.50527900 -0.06554900<br>H -3.09773100 2.17521500 -0.02618600<br>H -2.58524800 -3.13311000 1.28332200<br>H -2.83583000 -3.31349000 -0.47907700<br>H -4.23253100 -3.11927800 0.60780500<br>H 0.55075000 3.24673600 -1.47714500<br>H 0.74500200 3.35484200 0.29958900<br>H -0.31936900 4.49931600 -0.55664400<br>H 4.35802500 -1.91431200 -2.09086400<br>H 4.86408600 -1.30573900 -0.52389000<br>H 3.92361000 -2.80877500 -0.62039600<br>C 2.78240200 -1.01633000 -0.95392400<br>O 3.37896100 1.23480300 1.28411400<br>H 2.81784900 -0.00729400 -1.35937600<br>O 2.93108600 0.19312700 1.92863200<br>H 2.61718600 -0.43309500 1.23399500	= 0.268771<br>• Thermal Correction to Free Energy = 0.200742

Name	HPns HT 1-OH···•OOH Product Complex
Cartesian Coordinate:	Thermochemical Values:
O -0.49568900 2.97090100 -0.01624800<br>O -1.50837900 -1.60191500 -0.10599900<br>C 1.76850300 0.07411100 0.01055900<br>C 0.00365800 1.73641300 -0.01832100<br>C -0.52365900 -0.68576400 -0.06741500<br>C 0.81399700 -0.97403300 -0.03179600<br>C 1.34388300 1.41792800 0.02028800<br>C -1.01775400 0.69191400 -0.07533800<br>C 3.20052300 -0.18113700 0.04830200<br>C 3.80286100 -1.38136700 0.03219700<br>C 0.41896000 4.05973800 0.04076000<br>C -1.14276100 -2.97611700 -0.21999100<br>C 5.28234400 -1.56973200 0.07243000<br>H 1.14853700 -2.00162100 -0.03072700<br>H 2.09530200 2.19574500 0.05633300<br>H 3.82787000 0.70666500 0.09129200<br>H 3.20751300 -2.28967500 -0.01283500<br>H 1.07943100 4.05673400 -0.83036700<br>H 1.01131100 4.01979000 0.95871700<br>H -0.19043300 4.96015100 0.03615800<br>H -0.58830100 -3.30355300 0.66273000<br>H -0.54895200 -3.13783700 -1.12242800<br>H -2.07760500 -3.52815100 -0.29332400<br>H 5.56838600 -2.16869700 0.94272800<br>H 5.62279500 -2.11888100 -0.81096800<br>H 5.80830400 -0.61492000 0.11609900<br>O -2.22529400 0.96321600 -0.13017900<br>H -3.76780400 0.03351700 -0.48848000<br>O -4.43287500 -0.67846400 -0.44876800<br>O -4.16971000 -1.22638400 0.83968600<br>H -3.24411600 -1.49983300 0.74707600	• Electronic Energy = -804.080550<br>• Zero Point Energy Coorection = 0.250460<br>• Thermal Correction to Energy = 0.268630<br>• Thermal Correction to Enthalpy = 0.269574<br>• Thermal Correction to Free Energy = 0.202643

Name	HPns HT 3'-CH···•OOH Product Complex
Cartesian Coordinate:	Thermochemical Values:
O 2.84689500 -1.81578700 0.17449500<br>O 1.48447600 2.60052600 -0.07110300<br>O 3.33546000 0.78414200 0.33549400<br>C -0.51770200 -0.46674200 -0.60644300<br>C 1.80261400 -0.99146100 -0.06197200<br>C 1.06504900 1.31370800 -0.19689200<br>C -0.22217700 0.91312300 -0.50988700<br>C 0.51429700 -1.40373800 -0.37342400<br>C 2.08589100 0.37838300 0.02852100<br>C -1.82979700 -0.95796800 -0.90160500<br>C -2.98296000 -0.17880400 -1.14586400<br>C 2.60468900 -3.21214100 0.10823600<br>C 0.52573300 3.63237100 -0.26142400<br>H -0.99130800 1.65346400 -0.67998600<br>H 0.27907900 -2.45763500 -0.44392700<br>H -1.95224700 -2.03797500 -0.90899800<br>H -2.88676200 0.90288600 -1.17692200<br>H 3.33435800 1.75092700 0.35667400<br>H 2.27447100 -3.50852800 -0.89159300<br>H 1.86092900 -3.51880400 0.84937600<br>H 3.55599000 -3.69099600 0.33100600<br>H -0.28458100 3.54711600 0.46749000<br>H 0.12092600 3.60341800 -1.27646600<br>H 1.05716700 4.56898500 -0.10783700	• Electronic Energy = -804.067168<br>• Zero Point Energy Coorection = 0.248814<br>• Thermal Correction to Energy = 0.267372<br>• Thermal Correction to Enthalpy = 0.268316<br>• Thermal Correction to Free Energy = 0.200502

H -3.70633900 -0.63915800 1.13773500<br>O -3.26425100 -0.64465800 2.00187700<br>O -2.46467500 0.52704500 1.93385000<br>H -1.62349600 0.19280100 1.58645400<br>C -4.22455100 -0.71163000 -1.31915200<br>H -5.08635400 -0.08136000 -1.49997500<br>H -4.38159300 -1.78589900 -1.30050700	

Name	HPns RAF 1-C···•OOH Product
Cartesian Coordinate:	Thermochemical Values:
O 2.02714600 -2.04928500 -0.27243300<br>O 0.89754800 2.49697700 -0.27266600<br>O 2.34673000 0.43838700 -1.32813200<br>C -1.31557900 -0.46730500 -0.08901700<br>C 1.01359800 -1.17341900 -0.21958800<br>C 0.39024400 1.24564300 -0.20152400<br>C -0.91693800 0.90848000 -0.13448500<br>C -0.30992000 -1.47621800 -0.14423100<br>C -2.69454800 -0.86793700 -0.00414700<br>C -3.78060700 -0.06631100 0.06267100<br>C 1.70013200 -3.42921600 -0.23631200<br>C -0.02508700 3.57567900 -0.34271500<br>C -5.18383900 -0.57462700 0.14482000<br>H -1.67365100 1.68151800 -0.13088600<br>H -0.62918100 -2.51095500 -0.12295600<br>H -2.86379600 -1.94374000 0.00658100<br>H -3.66006400 1.01398300 0.05924800<br>H 2.55532100 1.38184800 -1.36329700<br>H 1.08013400 -3.70489500 -1.09432200<br>H 1.17967200 -3.68070100 0.69233900<br>H 2.64586700 -3.96468700 -0.28196800<br>H -0.63941900 3.61719300 0.56101100<br>H -0.66649100 3.47682600 -1.22276500<br>H 0.57210100 4.48137600 -0.42099900<br>H -5.67680200 -0.22047800 1.05619000<br>H -5.78270800 -0.21019300 -0.69632200<br>H -5.21275000 -1.66594200 0.14001500<br>C 1.51741100 0.24320800 -0.23093300<br>O 2.41171700 0.47640000 0.88873200<br>O 1.68937800 0.32572900 2.09626400<br>H 1.42241500 1.23531600 2.29678400	• Electronic Energy = -804.060997<br>• Zero Point Energy Coorection = 0.250529<br>• Thermal Correction to Energy = 0.267830<br>• Thermal Correction to Enthalpy = 0.268774<br>• Thermal Correction to Free Energy = 0.205173

Name	HPns RAF 2-C···•OOH Product
Cartesian Coordinate:	Thermochemical Values:
O -2.05967500 2.34584700 -0.10158500<br>O -1.28268100 -2.05078400 -0.96876100<br>O -2.93270300 -0.16226800 -0.05997700<br>C 1.16196300 0.46402500 -0.28463900<br>C -1.12218200 1.36666600 -0.13792500<br>C 0.71609200 -0.81537800 -0.25360800<br>C 0.24664900 1.57487500 -0.22666200<br>C -1.61368500 0.06473300 -0.10279000<br>C 2.60117500 0.78946200 -0.37336900<br>C 3.58247300 0.05840100 0.15765900<br>C -1.60112000 3.68462800 -0.19519900<br>C -0.53177200 -3.23486900 -1.23217300<br>C 5.03702800 0.39207400 0.05536600<br>H 1.40571800 -1.64904600 -0.30866300<br>H 0.65146000 2.57718600 -0.26109200<br>H 2.84985600 1.71577900 -0.88911900<br>H 3.32538800 -0.83872000 0.71823100<br>H -3.07656200 -1.11317700 -0.18132200<br>H -1.06968300 3.85488400 -1.13623400<br>H -0.95087400 3.94020700 0.64670600<br>H -2.49208300 4.30874100 -0.16523500<br>H -0.18827200 -3.70136500 -0.30719300<br>H 0.31778900 -3.01563100 -1.88290700<br>H -1.21105900 -3.91226100 -1.74708000<br>H 5.47973100 0.50674000 1.04922200<br>H 5.58382200 -0.41454500 -0.44266900<br>H 5.19619900 1.31472800 -0.50579400<br>C -0.72169600 -1.13750300 -0.06644100<br>O -0.94472800 -1.84311000 1.20863600<br>O -0.42423900 -1.07072400 2.27238900<br>H -1.19876300 -0.56787800 2.56621000	• Electronic Energy = -804.043612<br>• Zero Point Energy Coorection = 0.249797<br>• Thermal Correction to Energy = 0.267280<br>• Thermal Correction to Enthalpy = 0.268224<br>• Thermal Correction to Free Energy = 0.204268

Name	HPns RAF 3-C···•OOH Product
Cartesian Coordinate:	Thermochemical Values:
O 2.70501500 -1.81137300 -0.02947900<br>O 1.08071300 2.53881700 -0.17927800<br>O 3.09911900 0.78814700 -0.13270600<br>C -0.81597200 -0.72352600 -0.22617100<br>C 1.59099800 -1.06646400 -0.10285900<br>C 0.77046600 1.20997000 -0.21315000<br>C 0.29147700 -1.56129400 -0.15253500<br>C 1.81484100 0.35229000 -0.15172800<br>C -2.14145900 -1.28595600 -0.31055700<br>C -3.30693300 -0.60935800 -0.37202300<br>C 2.55491600 -3.22262500 0.03344200<br>C 0.20836600 3.42749100 -0.87290600<br>C -4.64506900 -1.26756700 -0.48299800<br>H 0.12285600 -2.63133900 -0.15492800<br>H -2.18202600 -2.37463900 -0.33173800<br>H -3.30184600 0.47653100 -0.33477500<br>H 3.07087500 1.75429500 -0.14143100<br>H 2.06910800 -3.60512800 -0.86842800<br>H 1.98092900 -3.51406900 0.91732500<br>H 3.56232800 -3.62688400 0.10310100<br>H -0.76845300 3.47387100 -0.38711600<br>H 0.09788300 3.12486700 -1.91828400<br>H 0.67902200 4.40774800 -0.82841300<br>H -5.29017800 -0.98716400 0.35597800<br>H -5.16246100 -0.95019700 -1.39422900<br>H -4.55500800 -2.35553800 -0.50052800<br>C -0.65414300 0.77058400 -0.17558400<br>O -1.32914100 1.35289200 0.97403900<br>H -1.23089200 1.23816800 -0.98566300<br>O -0.78182300 0.81120500 2.16278700<br>H -1.28812000 -0.00930800 2.26863400	• Electronic Energy<br>= -804.051492<br>• Zero Point Energy Coorection<br>= 0.250852<br>• Thermal Correction to Energy<br>= 0.268167<br>• Thermal Correction to Enthalpy<br>= 0.269111<br>• Thermal Correction to Free Energy<br>= 0.205800

Name	HPns RAF 4-C···•OOH Product
Cartesian Coordinate:	Thermochemical Values:
O 2.37832300 -1.74541600 -0.68128800<br>O 1.03695800 2.67957200 -0.36347400<br>O 2.82106900 0.84903200 -0.90768600<br>C 1.35882000 -0.94495500 -0.30060300<br>C 0.61339400 1.40687100 -0.13762000<br>C -0.60363500 1.03477700 0.32132800<br>C 0.15449900 -1.36089200 0.17559800<br>C 1.62517900 0.44902300 -0.45292900<br>C -2.24320400 -0.85038200 -0.01776200<br>C -2.93937400 -0.19240900 -0.93968500<br>C 2.17784500 -3.14404700 -0.55877100<br>C 0.13301500 3.73189900 -0.05510300<br>C -4.21263900 -0.69235800 -1.54817200<br>H -1.36500600 1.75958300 0.57734100<br>H -0.08193800 -2.41319800 0.27155200<br>H -2.58744800 -1.82021500 0.33814900<br>H -2.57050700 0.76570500 -1.29995100<br>H 2.81287100 1.81678500 -0.94757900<br>H 1.34532600 -3.47744200 -1.18514500<br>H 1.98910200 -3.42334200 0.48217800<br>H 3.09962600 -3.61103400 -0.89933200<br>H -0.13521400 3.71052200 1.00423800<br>H -0.76846600 3.65778300 -0.66930200<br>H 0.65657000 4.65764600 -0.28380900<br>H -5.02424100 0.02313100 -1.38723500<br>H -4.10278500 -0.80959900 -2.63045000<br>H -4.50821600 -1.65333100 -1.12337200<br>C -0.92708900 -0.40044100 0.58230200<br>O -1.20180000 -0.58093100 2.01970600<br>O -0.04915300 -0.25186700 2.77363900<br>H 0.53627700 -1.00863100 2.61404100	• Electronic Energy<br>= -804.044263<br>• Zero Point Energy Coorection<br>= 0.250169<br>• Thermal Correction to Energy<br>= 0.267484<br>• Thermal Correction to Enthalpy<br>= 0.268428<br>• Thermal Correction to Free Energy<br>= 0.205020

Name	HPns RAF 1'-C···•OOH Product
Cartesian Coordinate:	Thermochemical Values:
O 2.85719700 -1.82291200 -0.01615300<br>O 1.51889500 2.61252400 -0.10441500<br>O 3.40334100 0.77734500 -0.04255700<br>C -0.56186900 -0.43087800 -0.10158300<br>C 1.79791900 -0.97962300 -0.04571400<br>C 1.07566900 1.32395200 -0.09654000<br>C -0.25717200 0.92983500 -0.12092200<br>C 0.45970500 -1.37805800 -0.06329200<br>C 2.10705300 0.38165700 -0.06099300	• Electronic Energy<br>= -804.057902<br>• Zero Point Energy Coorection<br>= 0.249942<br>• Thermal Correction to Energy

C -2.88378000 -0.23066900 -1.08208400<br>C 2.58290100 -3.21341000 0.01767000<br>C 0.54300500 3.64387900 -0.09894200<br>C -4.32587100 -0.59061400 -1.15505700<br>H -1.05206900 1.66292400 -0.13642700<br>H 0.20384100 -2.42963700 -0.04773800<br>H -2.47655800 0.52875600 -1.73649700<br>H 3.41160600 1.74352500 -0.04446000<br>H 2.04066200 -3.53190000 -0.87776600<br>H 2.01027000 -3.48213700 0.91029300<br>H 3.55138200 -3.70881200 0.04753200<br>H -0.08854300 3.57624600 0.79090600<br>H -0.07602800 3.59890200 -0.99908300<br>H 1.09470600 4.58151700 -0.08431900<br>H -4.94726300 0.05134600 -0.51325300<br>H -4.71271400 -0.47891800 -2.17068700<br>H -4.49522900 -1.62029300 -0.82671400<br>C -1.99672300 -0.90119000 -0.09287000<br>O -2.57045200 -0.86484800 1.24142600<br>H -2.01302600 -1.98746500 -0.26124400<br>O -2.71451700 0.48703700 1.65557100<br>H -3.65176300 0.65350700 1.48044500	= 0.267658<br>• Thermal Correction to Enthalpy<br>= 0.268602<br>• Thermal Correction to Free Energy<br>= 0.203725

Name	HPns RAF 2'-C⋯•OOH Product
Cartesian Coordinate:	Thermochemical Values:
O -3.07288200 -1.68485800 0.14120200<br>O -1.42131100 2.63662200 -0.08487900<br>O -3.41350600 0.94295300 0.16117400<br>C 0.44114000 -0.56207000 -0.28375600<br>C -1.95604600 -0.93419400 0.00368700<br>C -1.06781200 1.32310000 -0.11852400<br>C 0.21818100 0.84017900 -0.26431900<br>C -0.67340400 -1.43272900 -0.14340300<br>C -2.16346300 0.45563200 0.01621200<br>C 1.73280000 -1.10914000 -0.43867700<br>C -2.91439400 -3.09390700 0.12348300<br>C -0.38463600 3.60185600 -0.20234100<br>C 4.20421100 -1.14498300 -0.86555200<br>H 1.05346100 1.52035600 -0.35111300<br>H -0.49714000 -2.50027800 -0.15727200<br>H 1.83517400 -2.18934500 -0.44017100<br>H -3.35514000 1.90815600 0.14917500<br>H -2.27917200 -3.43047200 0.94815700<br>H -2.49283800 -3.43152400 -0.82792100<br>H -3.91296400 -3.50907100 0.24391000<br>H 0.13298500 3.50027400 -1.15978900<br>H 0.33025600 3.50491400 0.61897000<br>H -0.87056600 4.57377500 -0.15140800<br>H 4.06666000 -1.70002300 -1.79634400<br>H 5.08981500 -0.51497000 -0.96168800<br>H 4.36447300 -1.85908000 -0.05583400<br>C 2.97892300 -0.29360200 -0.58859300<br>O 3.19587900 0.57528500 0.54639800<br>H 2.85411800 0.45074100 -1.38571500<br>O 3.31162100 -0.21203200 1.72198100<br>H 2.38946300 -0.28806300 2.01073000	• Electronic Energy<br>= -804.078791<br>• Zero Point Energy Coorection<br>= 0.251263<br>• Thermal Correction to Energy<br>= 0.268276<br>• Thermal Correction to Enthalpy<br>= 0.269220<br>• Thermal Correction to Free Energy<br>= 0.205965

Name	HPs (4-propylsyringol)
Cartesian Coordinate:	Thermochemical Values:
O -2.12549500 -1.93054900 0.17688900<br>O -1.17919600 2.60481400 0.08908700<br>O -2.85918100 0.58303600 0.48196000<br>C 2.49178200 -0.54992500 -1.00676400<br>C 1.05667300 -0.24390400 -0.64775000<br>C 3.30679000 -1.07616400 0.18487500<br>C 0.63263200 1.07310300 -0.47890300<br>C 0.15849600 -1.29098800 -0.43448200<br>C -1.15020600 -1.00660200 -0.05620400<br>C -0.68202200 1.35827900 -0.09782600<br>C -1.57946000 0.31140500 0.11562000<br>C 3.39390200 -0.07721800 1.33458800<br>C -1.78310200 -3.30068400 0.03442500<br>C -0.29612800 3.69551600 -0.10775100<br>H 2.51552800 -1.29140500 -1.81109600<br>H 2.96788200 0.35710800 -1.39181800<br>H 2.85916800 -2.00977900 0.54144500<br>H 4.31343300 -1.32381200 -0.16579500<br>H 1.33860500 1.87708500 -0.64356500<br>H 0.48533900 -2.31524700 -0.56730200<br>H 3.82361900 0.87067500 0.99557500<br>H 2.40576800 0.13442200 1.75105500	• Electronic Energy<br>= -654.373880<br>• Zero Point Energy Coorection<br>= 0.256607<br>• Thermal Correction to Energy<br>= 0.271424<br>• Thermal Correction to Enthalpy<br>= 0.272368<br>• Thermal Correction to Free Energy<br>= 0.214524

H 4.02288000 -0.46000000 2.14175900<br>H -3.31743300 -0.26185200 0.57728800<br>H -0.98994600 -3.57899900 0.73369900<br>H -1.46892800 -3.51938200 -0.98977900<br>H -2.68507400 -3.86365600 0.26543800<br>H 0.07168200 3.72740800 -1.13768000<br>H 0.55057800 3.64958400 0.58374500<br>H -0.87826400 4.59254000 0.09457300	

Name	HPs 1-OH radical
Cartesian Coordinate:	Thermochemical Values:
O -2.00517500 -2.08862000 0.19267100<br>O -1.33467900 2.54796600 0.10242800<br>O -2.86869400 0.41169400 0.50288900<br>C 2.45217500 -0.40330300 -1.01894800<br>C 1.01313100 -0.17096700 -0.64658300<br>C 3.29142100 -0.90692600 0.16891100<br>C 0.52980400 1.13520400 -0.47982100<br>C 0.17986200 -1.28266400 -0.42228600<br>C -1.13209700 -1.10505500 -0.03638700<br>C -0.77910900 1.35042600 -0.08878000<br>C -1.69466900 0.23455200 0.15667000<br>C 3.32924800 0.08087000 1.33054300<br>C -1.55474800 -3.42636400 0.02813500<br>C -0.52528300 3.69827700 -0.10344800<br>H 2.49974900 -1.14047000 -1.82634400<br>H 2.88609700 0.52672400 -1.39621700<br>H 2.89133600 -1.86650000 0.51128300<br>H 4.30691600 -1.09808200 -0.18955200<br>H 1.20176000 1.96499500 -0.65889800<br>H 0.58844400 -2.27571300 -0.56287300<br>H 3.70636600 1.05446800 1.00254100<br>H 2.33368000 0.23353400 1.75549900<br>H 3.98166100 -0.27908400 2.12923800<br>H -0.73350200 -3.64795000 0.71511600<br>H -1.23575600 -3.60567800 -1.00227600<br>H -2.40763000 -4.05947000 0.26197000<br>H -0.17474700 3.74736100 -1.13781800<br>H 0.32895200 3.69982400 0.57902900<br>H -1.16334600 4.55343100 0.10737400	• Electronic Energy = -653.738697<br>• Zero Point Energy Cooroection = 0.244141<br>• Thermal Correction to Energy = 0.258527<br>• Thermal Correction to Enthalpy = 0.259471<br>• Thermal Correction to Free Energy = 0.202026

Name	HPs 1'-CH radical
Cartesian Coordinate:	Thermochemical Values:
O -2.92176000 -0.94827100 -0.11348300<br>O -0.07729700 2.70617900 0.00607400<br>O -2.50087900 1.64754200 -0.18512400<br>C 1.86924700 -1.77899700 0.39802100<br>C 0.77431300 -0.89940000 0.25069300<br>C 3.29833300 -1.34763900 0.47651800<br>C 0.94342700 0.51012400 0.20661400<br>C -0.54117500 -1.42555000 0.14243800<br>C -1.61664100 -0.57193800 -0.00009100<br>C -0.14679500 1.35487900 0.06188100<br>C -1.43960500 0.81982100 -0.04206500<br>C 3.90346700 -1.06830300 -0.90998300<br>C -3.20900700 -2.33873800 -0.10807200<br>C 1.20756100 3.29739900 0.09518300<br>H 1.65627900 -2.84331000 0.39487100<br>H 3.39407600 -0.44973900 1.09587900<br>H 3.88322800 -2.12935500 0.96766200<br>H 1.93803700 0.92606600 0.28337000<br>H -0.68254700 -2.49814400 0.17476100<br>H 3.86545600 -1.96458400 -1.53379500<br>H 3.34898400 -0.27949900 -1.42414700<br>H 4.94741400 -0.75608600 -0.82387400<br>H -3.29288200 1.09657900 -0.24334600<br>H -2.89970300 -2.79634800 0.83549900<br>H -2.71464700 -2.84063500 -0.94422600<br>H -4.28805500 -2.42538300 -0.21783300<br>H 1.85067300 2.97299400 -0.72853600<br>H 1.68636600 3.06217500 1.05033100<br>H 1.04804300 4.37172300 0.02682200	• Electronic Energy = -653.725499<br>• Zero Point Energy Cooroection = 0.242869<br>• Thermal Correction to Energy = 0.257675<br>• Thermal Correction to Enthalpy = 0.258620<br>• Thermal Correction to Free Energy = 0.200691

Name	HPs HT 1-OH···•OOH Transition State
Cartesian Coordinate:	Thermochemical Values:

O -0.55065400 2.68670100 -0.43256100<br>O -1.55651100 -1.86491100 -0.51502500<br>C 2.86006000 -0.46226700 1.21651800<br>C 1.49341700 -0.17069000 0.65271800<br>C 3.97623200 -0.27177500 0.17538500<br>C 0.61896000 -1.21480500 0.34567500<br>C 1.12595700 1.15738300 0.40470700<br>C -0.10843100 1.45192800 -0.15838900<br>C -0.61811000 -0.93340600 -0.22082200<br>C 3.83342200 -1.19932800 -1.02719800<br>C 0.30193400 3.77940300 -0.12007100<br>C -1.18706100 -3.23911500 -0.41410500<br>H 3.04708300 0.20044600 2.06660800<br>H 2.88771600 -1.48991000 1.58955800<br>H 3.98085900 0.77030300 -0.15963800<br>H 4.93647600 -0.44907500 0.66826200<br>H 0.91593200 -2.23422800 0.55529400<br>H 1.81679800 1.94949000 0.66465100<br>H 3.80636300 -2.24679200 -0.71124500<br>H 2.91421900 -0.99299400 -1.58168100<br>H 4.67265100 -1.07928400 -1.71597600<br>H 1.23687700 3.71999800 -0.68411500<br>H 0.51524200 3.81482500 0.95169400<br>H -0.24300100 4.67429900 -0.41205400<br>H -0.96259400 -3.51005000 0.62080900<br>H -0.32586800 -3.45222000 -1.05144600<br>H -2.05012000 -3.80298300 -0.76073100<br>C -1.00499100 0.40206700 -0.51042700<br>O -2.12691000 0.67512300 -1.13671400<br>H -2.99145100 0.35068000 -0.56672500<br>O -3.85171300 0.05431700 0.37666900<br>O -3.06110400 -0.39690200 1.38316200<br>H -2.73037200 -1.25352100 1.05854300	• Electronic Energy<br>= -805.264639<br>• Zero Point Energy Coorection<br>= 0.268851<br>• Thermal Correction to Energy<br>= 0.286394<br>• Thermal Correction to Enthalpy<br>= 0.287339<br>• Thermal Correction to Free Energy<br>= 0.221924

Name	HPs HT 1'-CH···•OOH Transition State
Cartesian Coordinate:	Thermochemical Values:
O -1.09189100 2.72641700 0.19566700<br>O -2.78059400 -1.54536600 -0.39113000<br>O -3.11402000 1.06002200 -0.02800300<br>C 2.10690900 -1.01257200 -0.29093500<br>C 0.74958600 -0.44405900 -0.27605000<br>C 3.25137900 -0.19597000 -0.86117900<br>C -0.35855500 -1.29826200 -0.37681500<br>C 0.54472600 0.93013400 -0.06862000<br>C -0.75064800 1.42258300 0.01099000<br>C -1.65655500 -0.80052800 -0.29627900<br>C -1.85646600 0.57087400 -0.10234700<br>C 3.09544700 0.03499900 -2.36707300<br>C -0.04018100 3.67552700 0.30855300<br>C -2.62297500 -2.94463300 -0.56581900<br>H 2.11684500 -2.05342700 -0.62174900<br>H 3.32983900 0.76525900 -0.34524200<br>H 4.18714700 -0.72675800 -0.66686500<br>H -0.18645500 -2.35760600 -0.51621300<br>H 1.38986000 1.59906200 0.02364200<br>H 3.03571200 -0.91732200 -2.90062100<br>H 2.18431800 0.59834200 -2.58357000<br>H 3.94573900 0.59356500 -2.76542600<br>H -3.05037200 2.01623400 0.09897200<br>H 0.57180800 3.68593800 -0.59723600<br>H 0.58558000 3.45991500 1.17882100<br>H -0.51993900 4.64343400 0.43640500<br>H -2.08561600 -3.38916100 0.27690700<br>H -2.09789900 -3.16953400 -1.49879900<br>H -3.62966900 -3.35520500 -0.60999100<br>H 2.36541400 -1.10628700 0.94946700<br>O 1.11472700 -1.19002800 2.61799600<br>O 2.42022200 -0.99977700 2.22664100<br>H 0.69413300 -0.32515300 2.48556700	• Electronic Energy<br>= -805.253460<br>• Zero Point Energy Coorection<br>= 0.267822<br>• Thermal Correction to Energy<br>= 0.285901<br>• Thermal Correction to Enthalpy<br>= 0.286845<br>• Thermal Correction to Free Energy<br>= 0.220077

Name	HPs HT 1-OH···•OOH Reactant Complex
Cartesian Coordinate:	Thermochemical Values:
O -0.26107700 2.85356800 0.33882400<br>O 1.83295200 -1.35047000 0.02343300<br>C -2.94016400 -1.01976700 -1.18719500<br>C -1.63254300 -0.40817900 -0.74582100<br>C -3.94196100 -1.17465700 -0.03233600<br>C -0.48880100 -1.17768700 -0.59874400<br>C -1.57567600 0.95693800 -0.42809800<br>C -0.40025600 1.54702700 0.02136400<br>C 0.68487600 -0.58627500 -0.13773200	• Electronic Energy<br>= -805.288417<br>• Zero Point Energy Coorection<br>= 0.273704<br>• Thermal Correction to Energy

C -3.43498500 -2.09278500 1.07554400<br>C -1.40361900 3.68284500 0.20712200<br>C 1.91220200 -2.01362000 1.29692500<br>H -3.38710800 -0.39793500 -1.96871300<br>H -2.74658000 -2.00237100 -1.62799800<br>H -4.16797000 -0.18663000 0.38227500<br>H -4.87938300 -1.56758400 -0.43727800<br>H -0.48771400 -2.23738300 -0.83142000<br>H -2.46972800 1.55714000 -0.54692600<br>H -3.18532900 -3.08161400 0.67815500<br>H -2.53701700 -1.68589500 1.54794900<br>H -4.19080200 -2.22555700 1.85310700<br>H -2.21317700 3.34696000 0.86201000<br>H -1.75490700 3.71020000 -0.82857900<br>H -1.08603200 4.67942400 0.50716600<br>H 1.08096400 -2.71360800 1.39991500<br>H 1.88757100 -1.27987100 2.10596600<br>H 2.85708600 -2.55561800 1.31342500<br>C 0.76157600 0.76367700 0.17351800<br>O 1.89028600 1.35503700 0.62413900<br>H 2.65802000 0.78210700 0.46272900<br>O 4.36807500 0.10418700 -0.23196200<br>O 4.18564200 -0.93502400 -0.99675700<br>H 3.25119000 -1.22523800 -0.80048200	= 0.292089<br>• Thermal Correction to Enthalpy<br>= 0.293033<br>• Thermal Correction to Free Energy<br>= 0.224487

Name	HPs HT 1'-CH⋯•OOH Reactant Complex
Cartesian Coordinate:	Thermochemical Values:
O -2.24427300 1.17128900 -1.24703800<br>O -0.43125200 -2.94233300 -0.13669600<br>O -2.39629000 -1.45711600 -1.12402100<br>C 2.31439800 1.10575900 0.71339800<br>C 1.04978500 0.42165600 0.25010100<br>C 3.18298400 1.59744900 -0.45448900<br>C 0.92795300 -0.96864500 0.30766400<br>C -0.00034600 1.18499100 -0.27028500<br>C -1.15161900 0.54892400 -0.73174200<br>C -0.22768500 -1.60707100 -0.15660400<br>C -1.27294600 -0.84289700 -0.68225700<br>C 3.65478500 0.46559500 -1.36183900<br>C -2.23169400 2.59187200 -1.30627400<br>C 0.59755400 -3.75290700 0.40929800<br>H 2.89568900 0.41000700 1.32585100<br>H 2.61619300 2.32889900 -1.03987500<br>H 4.04763400 2.12618000 -0.04245300<br>H 1.74357800 -1.55022200 0.71762700<br>H 0.08511400 2.26405700 -0.30265400<br>H 4.21048400 -0.28531100 -0.79152900<br>H 2.81086400 -0.03663700 -1.84194900<br>H 4.31204400 0.84144400 -2.14932000<br>H -3.00693900 -0.77040400 -1.42296500<br>H -1.42017500 2.94638100 -1.94714300<br>H -2.13285200 3.02079200 -0.30576000<br>H -3.18812600 2.88321600 -1.73487800<br>H 0.78094000 -3.50328900 1.45842400<br>H 1.52480800 -3.65272700 -0.16240300<br>H 0.23877600 -4.77769200 0.33920700<br>H 2.05352000 1.95673600 1.35020300<br>O -0.97843000 0.41523500 2.69795500<br>O -1.21620000 1.68767100 2.86021400<br>H -0.49900500 0.33224300 1.84448300	• Electronic Energy<br>= -805.285908<br>• Zero Point Energy Coorection<br>= 0.272901<br>• Thermal Correction to Energy<br>= 0.291978<br>• Thermal Correction to Enthalpy<br>= 0.292922<br>• Thermal Correction to Free Energy<br>= 0.223076

Name	HPs HT 1-OH⋯•OOH Product Complex
Cartesian Coordinate:	Thermochemical Values:
O -0.17990500 2.86856200 0.13666000<br>O 1.77735900 -1.38138500 -0.06186400<br>C -3.05527500 -0.96525900 -0.95244100<br>C -1.70803000 -0.37469500 -0.64275500<br>C -3.96711600 -1.01405200 0.28737100<br>C -0.58265200 -1.20977600 -0.52891400<br>C -1.60479600 1.00925700 -0.42453200<br>C -0.39102400 1.57485900 -0.09049900<br>C 0.64344300 -0.67599200 -0.19759500<br>C -3.39893100 -1.87570200 1.41036900<br>C -1.28889600 3.75425100 0.02784800<br>C 1.70056200 -2.80410300 -0.15706600<br>H -3.53992200 -0.36500500 -1.72807900<br>H -2.92856500 -1.97708500 -1.34656600<br>H -4.13495800 0.00499300 0.64976200<br>H -4.94031000 -1.40342100 -0.02417100<br>H -0.70074800 -2.27112100 -0.70536100<br>H -2.49326600 1.62003500 -0.52495800	• Electronic Energy<br>= -805.300094<br>• Zero Point Energy Coorection<br>= 0.274113<br>• Thermal Correction to Energy<br>= 0.292584<br>• Thermal Correction to Enthalpy<br>= 0.293528<br>• Thermal Correction to Free Energy<br>= 0.225515

H -3.20395100 -2.89408600 1.06056400<br>H -2.45951300 -1.46628500 1.79124200<br>H -4.09857200 -1.93763900 2.24675400<br>H -2.06590200 3.49190500 0.75055100<br>H -1.69945700 3.73784700 -0.98510500<br>H -0.90023500 4.74508100 0.25011700<br>H 1.38814800 -3.10727400 -1.15898600<br>H 1.00879400 -3.19754300 0.59102400<br>H 2.70538900 -3.17012500 0.04347700<br>C 0.81045600 0.75212700 0.05093700<br>O 1.90451500 1.24816100 0.36805100<br>H 3.53410900 0.58130700 0.85532100<br>O 4.34455100 0.03803600 0.84870400<br>O 4.40465700 -0.34628900 -0.52153200<br>H 3.57310000 -0.83376300 -0.62207300	

Name	HPs HT 1’-CH⋯•OOH Product Complex
Cartesian Coordinate:	Thermochemical Values:
O -0.42067000 2.72719300 -0.32187400<br>O -3.02722900 -1.10134000 -0.33614800<br>O -2.76552400 1.53545700 -0.32134200<br>C 1.82225300 -1.64448800 -0.30991800<br>C 0.67629200 -0.81748500 -0.33965000<br>C 3.23058700 -1.14378100 -0.37929200<br>C -0.61713500 -1.40607600 -0.34523800<br>C 0.77548300 0.60023400 -0.33229200<br>C -0.37654400 1.36785300 -0.32587700<br>C -1.76174900 -0.62643800 -0.33605900<br>C -1.64995500 0.77489100 -0.32591800<br>C 3.66152800 -0.82555800 -1.82167000<br>C 0.81527500 3.42783500 -0.26717300<br>C -3.19352000 -2.50999500 -0.32708500<br>H 1.65980200 -2.71749800 -0.34312900<br>H 3.35500300 -0.24537600 0.23480300<br>H 3.90221700 -1.90070000 0.03358000<br>H -0.68930100 -2.48588600 -0.34370800<br>H 1.74636300 1.07544500 -0.32963800<br>H 3.59331900 -1.71720800 -2.44917100<br>H 3.01766100 -0.05799900 -2.25785400<br>H 4.69332100 -0.46663400 -1.84759800<br>H -2.48976900 2.46220200 -0.31033400<br>H 1.42311300 3.21135300 -1.14955100<br>H 1.36693900 3.16811100 0.64044700<br>H 0.56119400 4.48543100 -0.25035900<br>H -2.74981100 -2.95307500 0.56921300<br>H -2.75448400 -2.96471400 -1.21991800<br>H -4.26754300 -2.68428300 -0.32351900<br>H 1.79106200 -1.03012100 2.08050800<br>O 0.24634600 -0.21675600 2.78800500<br>O 1.66027400 -0.27881000 2.68061800<br>H 0.00024100 0.31304000 2.01320100	• Electronic Energy<br>= -805.281104<br>• Zero Point Energy Coorection<br>= 0.271873<br>• Thermal Correction to Energy<br>= 0.291208<br>• Thermal Correction to Enthalpy<br>= 0.292152<br>• Thermal Correction to Free Energy<br>= 0.222502

Name	HPs RAF 1-C⋯•OOH Product
Cartesian Coordinate:	Thermochemical Values:
O 1.42129700 -2.26762100 -0.46560500<br>O 1.18217900 2.41147500 -0.38865400<br>O 1.90074900 0.11778500 -1.66476300<br>C -2.75342600 -0.17290800 1.15789300<br>C -1.33796400 -0.08161200 0.66166400<br>C -3.74220500 -0.62371900 0.06715300<br>C -0.74129200 1.17187800 0.39555600<br>C -0.61791200 -1.27207400 0.37821600<br>C 0.64837200 -1.21966200 -0.11558500<br>C 0.52599700 1.27015900 -0.11075000<br>C -3.79294400 0.33499700 -1.11826100<br>C 0.87820600 -3.56947200 -0.30136300<br>C 0.47712800 3.62966200 -0.21042000<br>H -2.80306100 -0.88042900 1.99265100<br>H -3.06812800 0.80245100 1.54170200<br>H -3.46290200 -1.62420800 -0.27913600<br>H -4.73593700 -0.71227700 0.51674700<br>H -1.31775500 2.06805800 0.59186600<br>H -1.09973800 -2.22714600 0.55372600<br>H -4.05501300 1.34611200 -0.79108400<br>H -2.82628600 0.38827700 -1.62607200<br>H -4.53873300 0.01663000 -1.85039500<br>H 2.32960400 -0.73293100 -1.82998100<br>H -0.02556900 -3.69054100 -0.90528700<br>H 0.65075000 -3.76073100 0.75075000<br>H 1.64367400 -4.26321100 -0.64205700<br>H 0.19316600 3.76818200 0.83716100	• Electronic Energy<br>= -805.276647<br>• Zero Point Energy Coorection<br>= 0.274166<br>• Thermal Correction to Energy<br>= 0.291808<br>• Thermal Correction to Enthalpy<br>= 0.292752<br>• Thermal Correction to Free Energy<br>= 0.227907

H -0.41666400 3.65706200 -0.84050900<br>H 1.16069100 4.42099700 -0.51035800<br>C 1.38518600 0.06698600 -0.37423000<br>O 2.59289300 0.12054300 0.44194100<br>O 2.23252300 0.02565400 1.80735100<br>H 2.15988000 0.95561300 2.06888000	

Name	HPs RAF 2-C···•OOH Product
Cartesian Coordinate:	Thermochemical Values:
O 1.89812700 -1.10398300 -1.22206200<br>O -0.08995500 2.88658600 -0.35713700<br>O 2.14812700 1.54944200 -0.87691600<br>C -2.31161700 -1.30627800 1.12189400<br>C -1.07721300 -0.57443900 0.64663000<br>C -3.11139500 -1.93403100 -0.03057600<br>C -1.16956000 0.84181500 0.41337700<br>C 0.07362600 -1.24637900 0.41658300<br>C -0.08824500 1.55643000 -0.08784600<br>C 1.09958400 0.88922200 -0.36514100<br>C -3.61852000 -0.91585500 -1.04780900<br>C 2.05475300 -2.52143400 -1.25388000<br>C -1.29747000 3.59235200 -0.12418700<br>H -2.01536400 -2.09320000 1.82007000<br>H -2.95556500 -0.61152800 1.67042300<br>H -2.48031000 -2.67352800 -0.53430200<br>H -3.95871700 -2.47903000 0.39652200<br>H -2.10644900 1.33876200 0.62568500<br>H 0.15428300 -2.30537800 0.63715800<br>H -4.23464400 -0.15173500 -0.56348500<br>H -2.79130600 -0.41010700 -1.55257400<br>H -4.22914400 -1.40061300 -1.81318800<br>H 2.82306200 0.89811500 -1.12026600<br>H 1.09529700 -3.01205300 -1.43408900<br>H 2.49972900 -2.89307000 -0.32907000<br>H 2.72505500 -2.73068100 -2.08601400<br>H -1.58060500 3.55536700 0.93202200<br>H -2.11207700 3.19576800 -0.73767400<br>H -1.10071400 4.62426500 -0.40835300<br>C 1.30081100 -0.56551400 -0.07258800<br>O 2.40454700 -0.69600400 0.89208400<br>O 1.99448000 -0.19504000 2.14910300<br>H 2.29634200 0.72531000 2.11782700	• Electronic Energy<br>= -805.266642<br>• Zero Point Energy Coorection<br>= 0.273512<br>• Thermal Correction to Energy<br>= 0.291374<br>• Thermal Correction to Enthalpy<br>= 0.292318<br>• Thermal Correction to Free Energy<br>= 0.226975

Name	HPs RAF 3-C···•OOH Product
Cartesian Coordinate:	Thermochemical Values:
O 0.36319100 -2.64267500 -0.38431400<br>O 2.91679100 1.25084500 -0.12928300<br>O 2.70650900 -1.35096300 -0.56406600<br>C -1.95811500 1.68125000 0.67444300<br>C -0.72801800 0.89721700 0.31249300<br>C -3.11855300 1.52232500 -0.31967100<br>C 0.50875700 1.47851100 0.23995600<br>C 0.35983000 -1.27845900 -0.24037700<br>C 1.67477800 0.74051100 -0.04979200<br>C 1.56962500 -0.66300300 -0.29082400<br>C -2.72916800 1.89932200 -1.74578100<br>C -0.71250300 -3.19994500 -1.13884300<br>C 3.08028800 2.63876100 0.12453200<br>H -2.30142700 1.36506700 1.66665700<br>H -1.68847100 2.73902300 0.74954100<br>H -3.49108400 0.49341200 -0.29103700<br>H -3.94522800 2.15373200 0.01766000<br>H 0.58776600 2.54461300 0.41760800<br>H -2.34607100 2.92361500 -1.78534700<br>H -1.94483900 1.24184600 -2.13259200<br>H -3.58507500 1.83403300 -2.42152500<br>H 2.45475500 -2.27661200 -0.68336300<br>H -0.78660800 -2.71755700 -2.11783100<br>H -1.65756100 -3.10396800 -0.59959100<br>H -0.47846000 -4.25485800 -1.27005400<br>H 2.75397100 2.89240700 1.13683700<br>H 2.52894700 3.23851700 -0.60491300	• Electronic Energy<br>= -805.267123<br>• Zero Point Energy Coorection<br>= 0.274512<br>• Thermal Correction to Energy<br>= 0.292293<br>• Thermal Correction to Enthalpy<br>= 0.293238<br>• Thermal Correction to Free Energy<br>= 0.228001

H 4.14548300 2.83656000 0.02579600<br>C -0.90880500 -0.57471100 0.09562500<br>O -1.56410900 -1.21440700 1.22888100<br>O -0.75309200 -1.06521500 2.38303400<br>H -0.15315800 -1.82377400 2.32016900<br>H -1.67595900 -0.75739200 -0.67259000	

Name	HPs RAF 4-C···•OOH Product
Cartesian Coordinate:	Thermochemical Values:
O 2.40064800 1.49809700 -0.35559200<br>O -2.22757700 1.77979000 -0.24084000<br>O 0.16782700 2.83590800 -0.61344300<br>C -0.15734900 -2.58133500 -0.07410200<br>C 0.19718900 -2.61329700 -1.56284600<br>C -1.26518100 -0.35232400 0.35684900<br>C 1.24170100 -0.52368600 0.31082600<br>C 1.28358900 0.77683800 -0.07095200<br>C -1.18174300 0.94988900 -0.01589600<br>C 0.09359800 1.55027200 -0.23611100<br>C -0.65593400 -1.72024700 -2.46159600<br>C 3.65098200 0.83141000 -0.25176900<br>C -3.52327900 1.23982700 -0.04287600<br>H 0.52109400 -3.25323800 0.46161500<br>H -1.17331700 -2.95915100 0.07920500<br>H 1.25178400 -2.34958500 -1.69006800<br>H 0.09921100 -3.65205400 -1.89268800<br>H -2.22220300 -0.82931700 0.52398000<br>H 2.14205200 -1.11740400 0.41402600<br>H -1.72338900 -1.89152500 -2.29228100<br>H -0.45632200 -0.66117600 -2.28490500<br>H -0.44518400 -1.92879400 -3.51359900<br>H 1.10330500 3.05791800 -0.72742500<br>H 3.69668700 -0.01127600 -0.94684200<br>H 3.81692400 0.47937600 0.76991700<br>H 4.40941300 1.56561100 -0.51421600<br>H -3.65620800 0.90630600 0.99050200<br>H -3.70841800 0.40404800 -0.72480800<br>H -4.22189500 2.04582300 -0.25845400<br>C -0.05968000 -1.20239300 0.61087700<br>O -0.05644300 -1.61282200 2.02598100<br>O -0.04102100 -0.47227500 2.86399900<br>H 0.87564000 -0.16487500 2.79076700	• Electronic Energy = -805.270811<br>• Zero Point Energy Coorection = 0.274454<br>• Thermal Correction to Energy = 0.291877<br>• Thermal Correction to Enthalpy = 0.292821<br>• Thermal Correction to Free Energy = 0.229352

Name	•OOH
Cartesian Coordinate:	Thermochemical Values:
O 0.05508500 0.70765600 0.00000000<br>O 0.05508500 -0.59795000 0.00000000<br>H -0.88136700 -0.87764600 0.00000000	• Electronic Energy = -150.901030<br>• Zero Point Energy Coorection = 0.014495<br>• Thermal Correction to Energy = 0.017347<br>• Thermal Correction to Enthalpy = 0.018291<br>• Thermal Correction to Free Energy = -0.007646

Name	HOOH
Cartesian Coordinate:	Thermochemical Values:
O 0.70198200 0.11359800 -0.05967700<br>H 1.02308700 -0.62355700 0.47741100<br>O -0.70198300 -0.11359900 -0.05967500<br>H -1.02307700 0.62356600 0.47740500	• Electronic Energy = -151.545569<br>• Zero Point Energy Coorection = 0.027019<br>• Thermal Correction to Energy = 0.030217

	• Thermal Correction to Enthalpy = 0.031161<br>• Thermal Correction to Free Energy = 0.004771

5. **Figure S5: SOMO and HOMO density surfaces of the HT/PCET transition states, in aqueous solution, computed with isodensity value of 0.02 au**

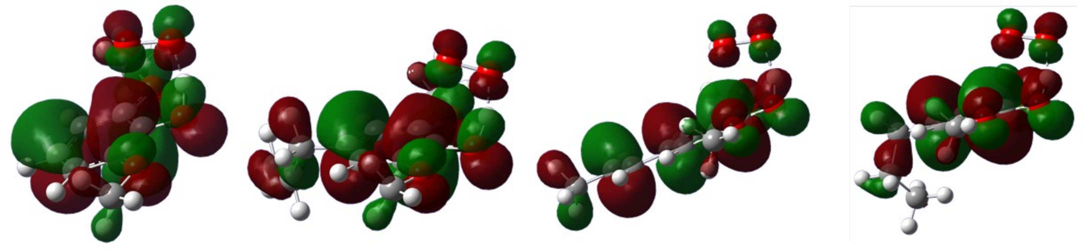

SOMO Hs HT 1-OH | SOMO HAs HT 1-OH | SOMO HPns HT 1-OH | SOMO HPs HT 1-OH

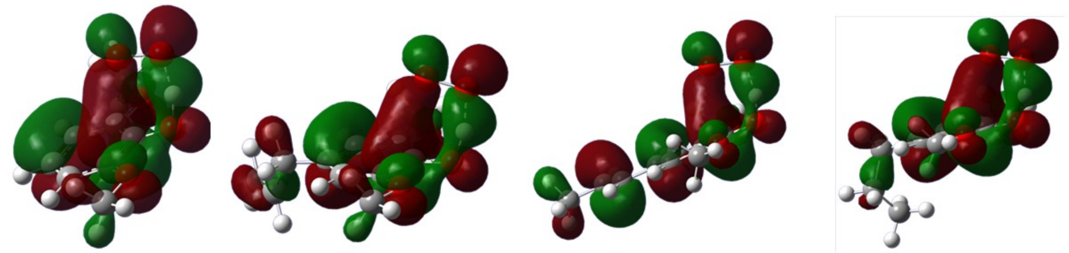

HOMO Hs HT 1-OH | HOMO HAs HT 1-OH | HOMO HPns HT 1-OH | HOMO HPs HT 1-OH

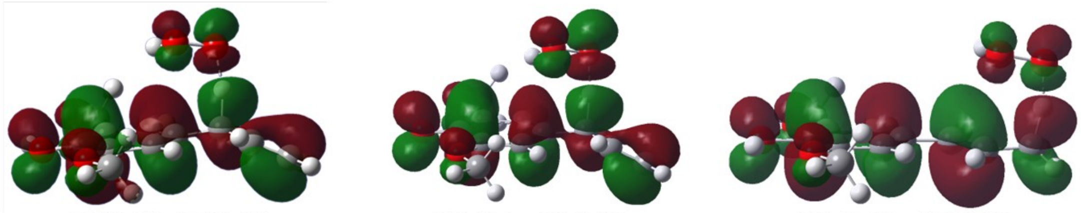

SOMO HAs HT 1′-CH | SOMO As⁻ HT 1′-CH | SOMO HPns HT 3′-CH

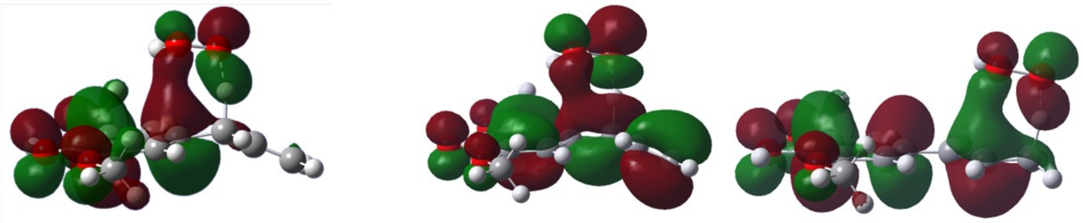

HOMO HAs HT 1′-CH | HOMO As⁻ HT 1′-CH | HOMO HPns HT 3′-CH

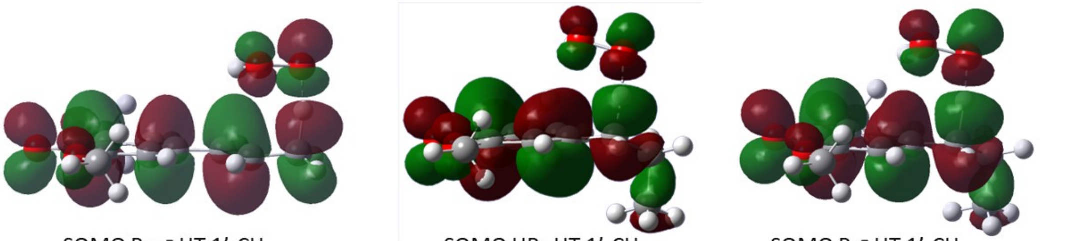

SOMO Pns⁻ HT 1′-CH | SOMO HPs HT 1′-CH | SOMO Ps⁻ HT 1′-CH

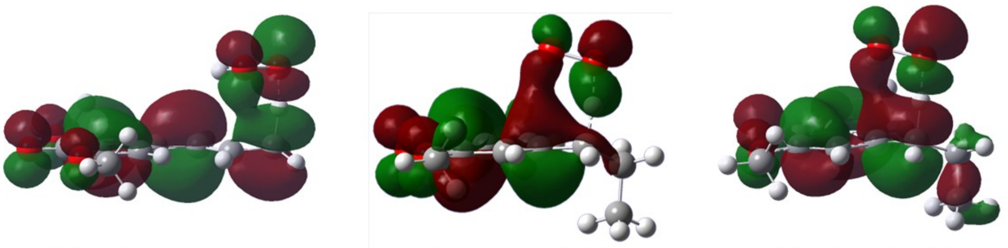

HOMO Pns⁻ HT 1′-CH | HOMO HPs HT 1′-CH | HOMO Ps⁻ HT 1′-CH

6. Figure S6. Boiled-Egg model and LightBBB prediction of studied syringol derivatives.

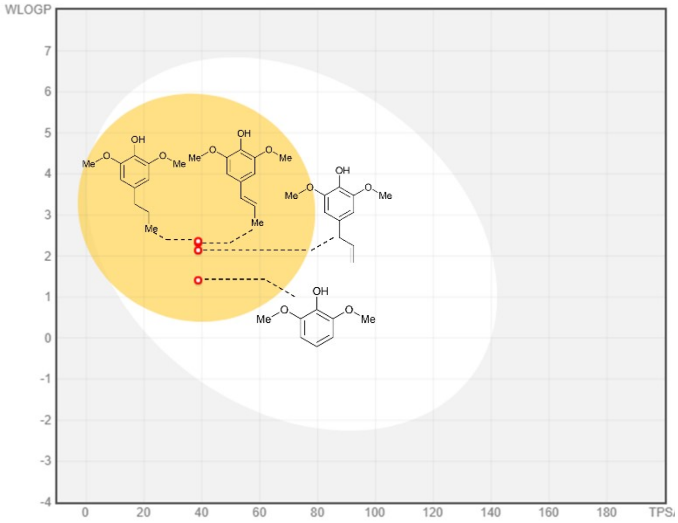

LightBBB Prediction	
Syringol	Permeable
4-Allylsyringol	Permeable
4-Propenylsyringol	Permeable
4-Propylsyringol	Permeable
